# Weekly sales forecasting using time series decomposition with irregular seasonality: A case study of Sony TV sales in the United States

**DOI:** 10.1371/journal.pone.0344222

**Published:** 2026-11-03

**Authors:** Arthur Matsuo Yamashita Rios de Sousa, Daisuke Ando, Junichi Ozaki, Yohei Shida, Qianyun Wu, Yixuan Y. Zheng, Zhihua Zhong, Rie Maskawa, Jiwei J. Jiang, Kay Nagayama, Hideki Takayasu, Misako Takayasu

**Affiliations:** 1 School of Computing, Institute of Science Tokyo, Yokohama, Japan; 2 Department of Data Science, Yokohama City University, Yokohama, Japan; 3 Institute of Systems and Information Engineering, University of Tsukuba, Tsukuba, Japan; 4 School of Engineering, Institute of Science Tokyo, Yokohama, Japan; Mercer University, UNITED STATES OF AMERICA

## Abstract

In the practice of sales forecasting, alongside high accuracy, simplicity and interpretability are desirable qualities in a predictive model. Here, we use time series decomposition to construct a simple and interpretable forecasting method for weekly sales, taking as a case study the total weekly sales of Sony TV units in the United States. The main challenge is to properly address irregular seasonality arising from the way a year is divided into weeks and from floating holidays and events. As a solution, we propose the concept of ‘analogous weeks’, a general framework for grouping weeks that are similar according to some criteria, which encodes seasonality and already accounts for calendar effects. By incorporating this notion into the time series decomposition and forecasting, we achieved yearly average percentage errors of less than 10% for the 1-week-ahead forecast and less than 15% for forecast horizons of up to 26 weeks, accuracy levels comparable to or better than those of other established seasonal models, namely Prophet and SARIMAX, particularly at longer forecast horizons.

## Introduction

Demand forecasting is an important and challenging task in practical time series forecasting: accurately predicting the future sales of products enables a business to optimize the supply chain by managing its inventory more efficiently (preventing overstocking and minimizing waste), designing more effective distribution and marketing strategies and planning better workforce allocation [1,2]. It therefore plays a critical role in the long-term growth/stability of a business in the market.

Although qualitative methods based on human judgment (from expert opinions and information gathered in the field) are commonly used in a range of demand forecasting tasks, there has been a shift toward data-based quantitative methods, either with the intention to replace or to be integrated with judgmental forecasts [3–6]. In this context, the adoption of machine learning has gained substantial traction – see, for instance, the M5 Competition [7]. One of the main drawbacks of those methods, however, is the usual lack of interpretability/explainability (despite the efforts on explainable artificial intelligence [8–10]). Decision-makers need to know how a forecast is constructed so risks can be properly assessed and possible sources of errors in the model can be swiftly identified and adjusted [11,12]: “we do not use black box machine learning models for high-stakes decisions unless no interpretable model can be constructed that achieves the same level of accuracy” [13,14]. Besides interpretability, decision-makers also seek simplicity. Not only because of (often unconscious) aversion to complex algorithms [15], but simple forecasting models encourage “engagement and criticism by facilitating understanding” and aid “in detecting mistakes, important omissions, ludicrous variables, unsupported conclusions, and fraud” [16]. Additionally, sophisticated methods not always can outperform simple ones [17–20]. The class of statistical methods generally combines the requested simplicity and interpretability and it is then an alternative with solid theoretical foundations for demand forecasting.

The qualifier ‘statistical’ for a forecasting model – in opposition to ‘machine learning’ – refers to how structured its underlying mechanisms are: while machine learning models allow relationships within the data to be discovered (‘unstructured’), a statistical method “prescribes the data generating process”, “making assumptions about the structural components of the series and the way historical observations are correlated” (‘structured’) [12,21]. Under the label ‘statistical’ fall the classical time series methods such as exponential smoothing [22–24] and ARIMA (Autoregressive Integrated Moving Average) [25], together with their numerous extensions. Other relevant examples are methods based on the concept of time series decomposition, which assume that the time series can be separated into components that can be individually forecasted and then recombined. The idea of decomposing a time series into interpretable components dates back to the work of Pearsons in 1919, considered the classical decomposition: “each series is a composite consisting of four types of fluctuations (...): A long-time tendency or secular trend (...); A wave-like or cyclical movement superimposed upon the secular trend (...); A seasonal movement within the year (...); Residual variations (...)” [26]. Those approaches are standard in several national statistical agencies and central banks, notably the United States Census Bureau, which maintains the seasonal adjustment method/software X-13ARIMA-SEATS [27] (“an expansion of the Census Bureau’s earlier X-12-ARIMA program, which itself was an expansion of the initial X-11 software from the Census Bureau and X-11-ARIMA from Statistics Canada under Estela Dagum” [28]) and the Bank of Spain, which developed the TRAMO-SEATS (TRAMO: Time series Regression with ARIMA noise, Missing values and Outliers; SEATS: Signal Extraction in ARIMA Time Series) [29]. They are also recommended in official guidelines, e.g., from the European Statistical System [30] and the International Monetary Fund [31]. Finally, still on time series decomposition, it is worth mentioning STL (Seasonal and Trend decomposition using LOESS) [32,33], an iterative method to estimate time series components, popular among researchers and practitioners for its versatility.

In this work, we use time series decomposition in the task of building a simple and interpretable forecasting method for weekly sales, studying the case of total weekly sales of Sony TV units in the United States. Data with weekly granularity are not uncommon in business context, as planning and reporting activities often follow a weekly cycle. Forecasting weekly sales data can, nonetheless, be challenging. Following a strict definition, a seasonal pattern is always regular, with a known fixed period, e.g., monthly data typically presents seasonality with period of 12 months [19]. But for weekly data the seasonal period is not fixed (although known), since a year can have either 52 or 53 weeks. We refer to it as ‘irregular seasonality’, a frequently omitted issue that needs to be properly addressed. Another source of irregularity that disrupts expected regular patterns is the presence of floating holidays and other special events (such as promotions in retail) – this one, in its turn, is largely recognized as essential and it should be incorporated in forecasting models if the information is available [7,34–36]. Examples of statistical models that handle seasonal data in the presence of special days are the Prophet forecasting procedure developed by Facebook/Meta, based on time series decomposition as well and including both seasonal and holidays components [37,38], and the ARIMA extension SARIMAX (Seasonal ARIMA with Exogenous Regressors), which has additional autoregressive and moving average terms with lags corresponding to the fixed seasonality frequency and a term to incorporate external variables (e.g., presence of a holiday as dummy variable) [39].

The key concept we propose to deal with irregular seasonality is the one of ‘analogous weeks’ (straightforwardly extendable to other units of time: ‘analogous days’, ‘analogous months’, and so on): weeks that are similar to a given week of interest according to some criteria. The idea of using historical analogs is not new but it is usually applied solely to improve forecasts of special events: “There is evidence that forecasters often use information from analogous events from the past to help to estimate the effects of an anticipated special event” [40,41]. More than judgmental adjustments, we suggest here a unified framework by defining a set of analogous weeks for every week in the calendar, not only for weeks containing special events, which addresses the issue of irregular seasonality and already accounts for the effect of holidays and events. Although related concepts may exist in industry practice, the proposed framework provides a systematic and publicly documented approach to define and apply analogous weeks.

On the paper organization, we first describe the weekly TV sales data in the Data description section to then explain how to incorporate the idea of analogous weeks to time series decomposition in the Time series decomposition section and to a simple forecasting method in the Time series forecasting section. We apply our proposed method to the TV sales data in the TV sales forecasting section and compare its performance with other established methods for different forecast horizons. We conclude by pointing out possible uses and extensions of ‘analogous weeks’ in the research/practice of demand forecasting as well as in time series forecasting in general.

## Data description

We forecast the total weekly Sony TV sales in the United States based on historical sales time series data from week 1 of 2014 to week 52 of 2024. The sales refer to the total Sony TV units, across all TV models, sold from multiple dealers (e.g., Best Buy and Amazon) to end customers in the United States during the period of 1 week. Weeks are defined according to the ISO-8601 date and time standard, with exactly 7 days, starting from Monday and ending on Sunday; a year begins with the week containing the first Thursday of January and it can have either 52 or 53 weeks [42]. Fig 1 displays the sales for the 574 considered weeks. The data was provided by Sony Group Corporation under an agreement permitting its use for research purposes. In compliance with the terms and conditions established by the data provider, note that the actual sales numbers were masked by dividing them by a constant factor *q*; we use the masked data throughout this study (see S1 Appendix for details on the data masking).

**Fig 1:**
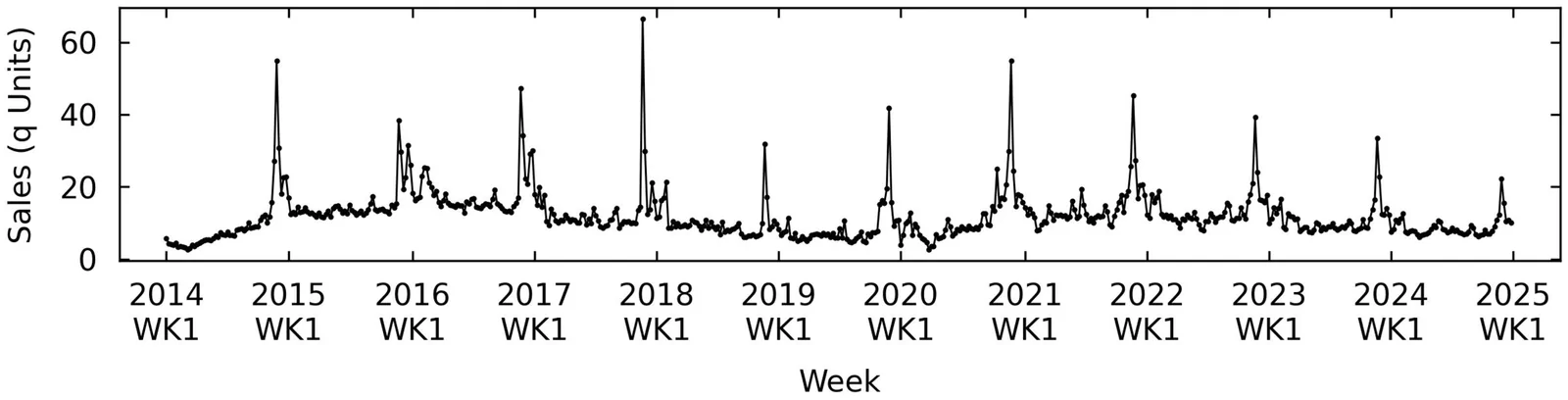
Time series of weekly Sony TV sales in the United States from week 1 of 2014 to week 52 of 2024.

The aggregation of sales across all Sony TV models and all dealers into a single total sales time series enables the analysis of the company’s performance in this specific product category. This is particularly relevant for a multi-sector company such as Sony, which has several other segments (cameras, video game consoles, audio equipment, etc): forecasting total sales of different categories supports decisions on strategic financial planning and resource allocation among divisions. From a tactical perspective, forecasting at the product category level also informs aggregate planning, which includes aspects that are not necessarily specific to individual TV models, such as workforce (e.g., need for hiring or subcontracting) and logistics (e.g., storage and transportation) [43,44]. Moreover, and of great relevance, an accurate forecast of the total TV sales is essential for more detailed, disaggregated forecasts – e.g., by individual TV model or by dealer – using top-down approaches based on historical or forecasted proportions [45–47].

For the forecasting task, it is important to identify exogenous factors that influence sales. There are several holidays and events throughout a year that affect TV sales in the United States, as it can be observed in Fig 2. The main of such holidays are: Easter (variable date), Memorial Day (last Monday of May), Independence Day (July 4), Labor Day (first Monday of September) and Christmas (December 25). There are usually promotions in those dates, which increase sales; Easter is an exception, with a possible decrease in sales because some retailers close their stores on Easter Sunday [48].

**Fig 2:**
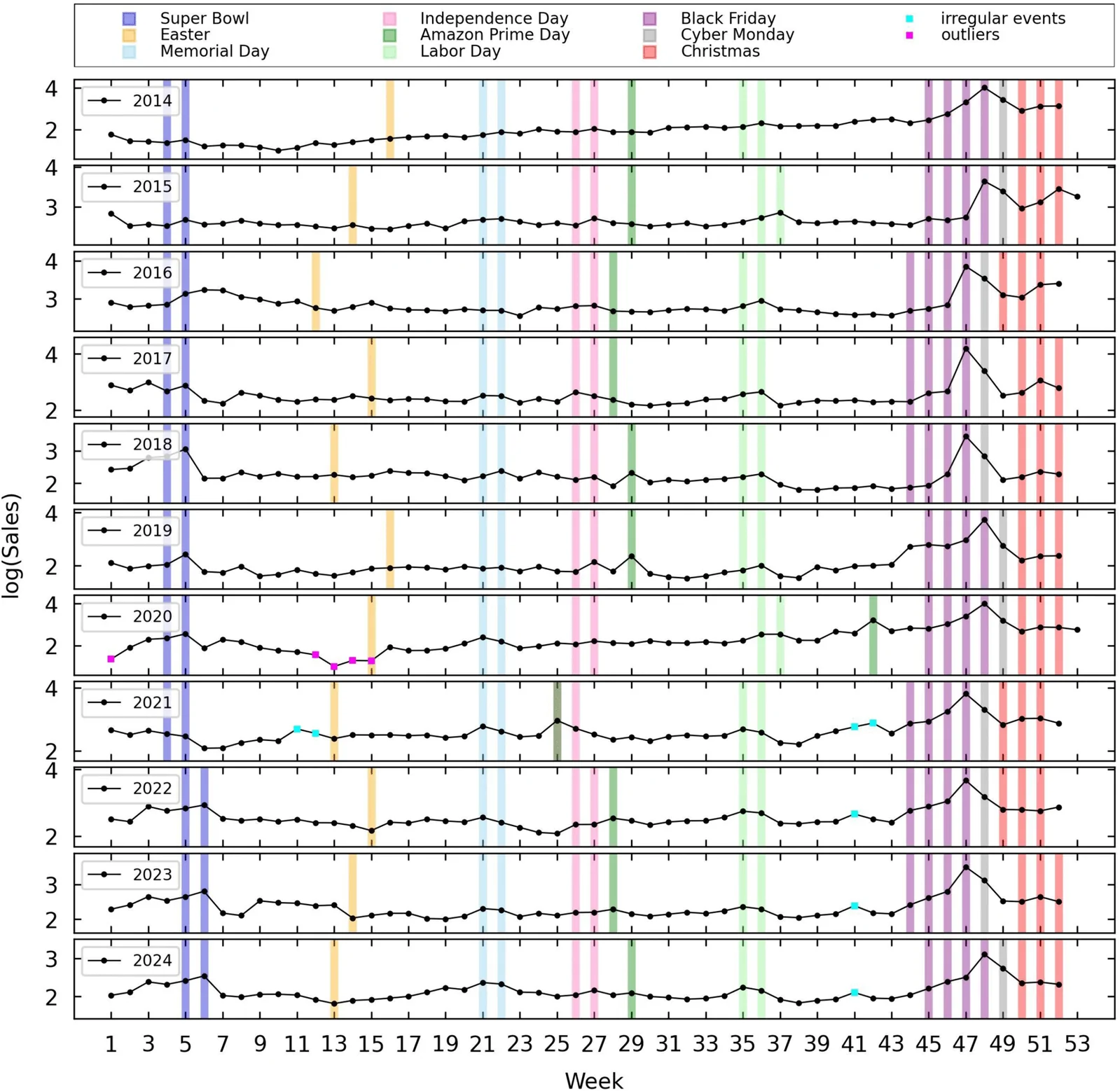
Time series of weekly Sony TV (log-)sales in the United States for each year from 2014 to 2024, highlighting weeks with holidays, regular and irregular events and outliers: Super Bowl and the week before it (blue shade), Easter (yellow shade), Memorial Day and the week before it (light blue shade), Independence Day and the week before it (pink shade), Amazon Prime Day (green shade – note that it coincides with the week before Independence Day week in 2021), Labor Day and the week before it (light green shade), Black Friday and the three weeks before it (violet shade), Cyber Monday (gray shade), Christmas and the two weeks before it (red shade), irregular events (cyan symbols) and outliers (magenta symbols).

Regarding events, we categorize them in regular and irregular: regular events occur every year, usually in the same period of the year, while irregular events do not. The important regular events for TV sales in the United States are:

- Super Bowl: it is the annual championship game of the National Football League of the United States, taking place on the first Sunday of February from 2004 to 2021 and on the second Sunday of February since 2022;
- Amazon Prime Day: it is an annual shopping event promoted by Amazon since 2015, usually held during Summer (although it happened in October in 2020 because of the COVID-19 pandemic) – for our analysis, when necessary, we artificially assign week 29 (Amazon Prime Day week in 2015) as Amazon Prime Day week in 2014, when the event did not exist yet;
- Black Friday: happening on the Friday after Thanksgiving (fourth Thursday in November), it is the most important shopping event in the United States: the Black Friday week alone accounts for 5% to 10% of the total annual Sony TV sales;
- Cyber Monday: online shopping event on the Monday following the Black Friday.

And the relevant irregular events in the considered years are:

- COVID-19 Economic Impact Payments: the United Sates federal government made direct payments to individuals to alleviate the financial impacts due to the COVID-19 pandemic; there were three of such payments: the first one in April of 2020 (a period we consider as outlier, as we explain later), the second one in December of 2020 (coinciding with the Christmas holiday) and the third one in March of 2021, which is the one we take as an irregular event (weeks 11 and 12) [49];
- Amazon Prime Day 2: since 2021, Amazon has an additional shopping event in October: Black Friday-Worthy Deals in 2021 (week 41) [50], Amazon Early Access Sale in 2022 (week 41) [51] and Prime Big Deals Days in 2023 and 2024 (week 41) [52,53] – because it is recent, it is still unknown if it will become a regular event;
- Best Buy Early Black Friday Deals: shopping event promoted by Best Buy in October of 2021 (week 42), before the Black Friday period [54].

In Fig 2, note that we additionally highlight weeks right before most holidays and events since promotions may begin prior to them, as is the case of the Black Friday, with discounts and deals during the whole month of November.

As mentioned, we also consider outlier weeks. The difference between outliers and irregular events is that irregular events are announced in advance, knowledge that can be used to improve forecast in those dates, while outliers are only identified after they happened. We opt here for only classifying a week as outlier if the cause is known, in particular weeks with some disruption in production or supply (directly informed by Sony prior to the analysis and without subsequent modifications): the first week of 2020 with a stock-out event and 4 weeks in March and April of 2020 related to the COVID-19 pandemic outbreak. Such a choice on how to handle outliers avoids the unintended removal of informative data (as may occur with automatic procedures) and increases transparency by relying only on documented causes (see S2 Appendix for tests on COVID-19 outliers).

In this study, we restrict our analysis to Sony’s internal data and consider only the described calendar information as exogenous factors influencing sales. Including external data could allow for additional explanatory variables, such as relevant macroeconomic indicators (unemployment rate, savings rate, consumer confidence index, etc). In this case, it is important to note that “explanatory variables also need forecasts which results in two statistical estimates: one for the sales prediction model and one for the prediction of the explanatory variables” and, thus, the final forecasts may be inaccurate not because of a poor forecasting model, but due to inaccurate predictions of the explanatory variables [55].

## Time series decomposition

In order to forecast weekly TV sales, we propose a time series decomposition method to be applied to the historical sales time series data, with the implicit assumptions that it can in fact be decomposed and that the extracted components can be independently forecasted.

### Analogous weeks

As presented in the Introduction section, analogous weeks are weeks that are similar to a given week according to some criteria or rules. Building on the general idea of rule-based approaches [56,57], with adaptations and extensions to the weekly sales setting considered here and its specific characteristics, such as years with either 52 or 53 weeks and the presence of irregular events as described in the Data Description section, we construct the following rules to define the set of analogous weeks of a given week:

(a) if the week has no holiday or event (ordinary week), the analogous weeks are the ones with the same week number, provided that they do not have a holiday or event either – if they do, then take the nearest week without event, giving priority to weeks that happened before;
(b) if the week has a holiday or regular event, the analogous weeks are the ones with same holiday or regular event;
(c) if the week has an irregular event, the analogous weeks are the ones with same irregular event or with some holiday or event assumed similar; for the irregular events in our data, we assume:
  - COVID-19 Economic Impact Payments: similar to Memorial Day week and the week before it;
  - Amazon Prime Day 2: similar to Amazon Prime Day week;
  - Best Buy Early Black Friday Deals: similar to Amazon Prime Day week.

Rules for ordinary weeks, holidays and regular events are straightforward due to their yearly recurrence. For irregular events, the assignment of analogous weeks requires forecaster judgment. Similarity in event characteristics should be preferred when available (e.g., Amazon Prime Day 2 similar to Amazon Prime Day). When such information is not available or is ambiguous, temporal proximity should be used as a secondary criterion (e.g., COVID-19 Economic Impact Payments assumed similar to Memorial Day week because it is the nearest regular holiday with increase in sales and Best Buy Early Black Friday Deals assumed similar to Amazon Prime Day because it occurs in the week after Amazon Prime Day 2) (see S3 Appendix for alternative judgments).

There are two additional categories of weeks which follow rules similar to weeks with irregular events (judgment guided by the temporal proximity criterion):

(d) gap week: week in between Cyber Monday week and second week before Christmas week (in years 2017, 2018 and 2023), assumed similar to the second week before Christmas week.
(e) last week: last week of the year not containing Christmas (in years 2015, 2016, 2021 and 2022), assumed similar to Christmas week.

An additional judgmental adjustment is needed in the case of holiday/event overlap, as it occurs for the week before Independence Day week and Amazon Prime Day week in 2021. In this case, we judge the event Amazon Prime Day as more important, setting this week as such and not including it in the set of analogous weeks of the week before Independence Day week (see S4 Appendix for the opposite judgment). It is important that such judgments be clearly documented so they can be reviewed if necessary.

Examples of analogous weeks for each rule in the considered years (see Fig 2):

(a) analogous weeks of week 12 of 2024 (2024-WK12): 2014-WK12, 2015-WK12, 2016-WK11, 2017-WK12, 2018-WK12, 2019-WK12, 2020-WK11, 2021-WK10, 2022-WK12, 2023-WK12, 2024-WK12;
(b) analogous weeks of Amazon Prime Day week of 2024 (2024-WK29): 2014-WK29, 2015-WK29, 2016-WK28, 2017-WK28, 2018-WK29, 2019-WK29, 2020-WK42, 2021-WK25, 2022-WK28, 2023-WK28, 2024-WK29;
(c) analogous weeks of Amazon Prime Day 2 week of 2024 (2024-WK41): 2014-WK29, 2015-WK29, 2016-WK28, 2017-WK28, 2018-WK29, 2019-WK29, 2020-WK42, 2021-WK41, 2022-WK41, 2023-WK41, 2024-WK41;
(d) analogous weeks of gap week of 2023 (2023-WK49): 2014-WK50, 2015-WK50, 2016-WK49, 2017-WK49, 2018-WK49, 2019-WK50, 2020-WK50, 2021-WK49, 2022-WK49, 2023-WK49, 2024-WK50;
(e) analogous weeks of last week of 2022 (2022-WK52): 2014-WK52, 2015-WK53, 2016-WK52, 2017-WK52, 2018-WK52, 2019-WK52, 2020-WK53, 2021-WK52, 2022-WK52, 2023-WK52, 2024-WK52.

### Proposed decomposition

Marginally deviating from the classical decomposition, we propose a decomposition into two components: the trend-cycle component and the seasonal-stochastic component (a combination of the traditional seasonal and residual components, allowing each set of analogous weeks to have an associated fluctuation to it, not necessarily all in the same scale). In a similar spirit of the theta model [58], those components are associated to different time scales, the trend-cycle one referring to the long-term and the seasonal-stochastic one, to the short-term.

Since we are modeling sales (non-negative quantities), we select the multiplicative model:


$$\begin{array}{c} X_{t}=C_{t}S_{t}, \end{array}$$ (1)

where $X_{t}$ represents the sales in week *t*, 1≤t≤n, $C_{t}$ is the trend-cycle component and $S_{t}$ is the seasonal-stochastic component.

We use a log transformation to work with additive decomposition:


$$\begin{array}{c} x_{t}=c_{t}+s_{t}, \end{array}$$ (2)

with $x_{t}=\log X_{t}$, $c_{t}=\log C_{t}$ and $s_{t}=\log S_{t}$. The task is to obtain the components $c_{t}$ and $s_{t}$ from the observed $x_{t}$.

For a given tuning parameter ℓ, $1\leq \ell \leq \lfloor \frac{n-1}{2}\rfloor$, define the following sets:


$$\begin{array}{cc} & U=[1,n] \end{array}$$ (3)

$$\begin{array}{cc} & U_{\ell }=[1+\ell ,n-\ell ] \end{array}$$ (4)

$$\begin{array}{cc} & L_{t}=[t-\ell ,t+\ell ]\cap U=[\max(1,t-\ell ),\min(n,t+\ell )] \end{array}$$ (5)

$$\begin{array}{cc} & A_{t}\text{: set of analogous weeks of week }t \end{array}$$ (6)

$$\begin{array}{cc} & O\text{: set of outlier weeks} \end{array}$$ (7)

$$\begin{array}{cc} & V=U⧵O\text{ (set of valid weeks in U)} \end{array}$$ (8)

As in standard methods such as STL and X-11 derived procedures, we iteratively determine both components simultaneously. This iterative strategy is commonly applied because it is difficult to isolate the components in a single step, as each would be biased by the presence of the other; iterations refine the estimates by alternating updates until convergence [59]. Define the initial iteration of the seasonally-adjusted time series $y_{t}^{\left(0\right)}=x_{t}$. Iteration *i* of the trend-cycle component $c_{t}^{\left(i\right)}$ is given by the value at week *t* of the local linear regression of $y_{t}^{\left(i\right)}$ on the set of valid weeks in window $L_{t}$:


$$\begin{array}{c} c_{t}^{\left(i\right)}=\alpha_{t}^{\left(i\right)}+\beta_{t}^{\left(i\right)}t. \end{array}$$ (9)

Moving averages or higher order polynomials could be used as well, but linear fits provide “a good balance between computational ease and the need for flexibility to reproduce patterns in the data” [32]. More importantly, the slopes of the local linear trends will be incorporated into the time series forecasting. We apply the ordinary least squares method to obtain the coefficients $\alpha_{t}^{\left(i\right)}$ and $\beta_{t}^{\left(i\right)}$. For notation, we use the Iverson bracket, defined for a proposition *P* as:


$$\begin{array}{c} ⟦P⟧=\left\{ \begin{array}{c} 1,\text{ if }P\text{ is true}\\ 0,\text{ otherwise} \end{array}\right.; \end{array}$$ (10)

it improves readability and is particularly useful for manipulating sums [60]. We have (observing that |.| denotes the number of elements in a set):


$$\begin{array}{c} \alpha_{t}^{\left(i\right)}=\sum_{j=1}^{n}y_{j}^{\left(i\right)}\frac{⟦j\in V\cap L_{t}⟧}{\left|V\cap L_{t}\right|}-\beta_{t}^{\left(i\right)}\mu_{t}, \end{array}$$ (11)

$$\begin{array}{cc} \beta_{t}^{\left(i\right)} & =\frac{1}{\nu_{t}}\sum_{j=1}^{n}(j-\mu_{t})\left(y_{j}^{\left(i\right)}-\sum_{k=1}^{n}y_{k}^{\left(i\right)}\frac{⟦k\in V\cap L_{t}⟧}{\left|V\cap L_{t}\right|}\right)\frac{⟦j\in V\cap L_{t}⟧}{\left|V\cap L_{t}\right|}\\ & =\sum_{j=1}^{n}\frac{j-\mu_{t}}{\nu_{t}}y_{j}^{\left(i\right)}\frac{⟦j\in V\cap L_{t}⟧}{\left|V\cap L_{t}\right|}, \end{array}$$ (12)

where we define:


$$\begin{array}{c} \mu_{t}=\sum_{j=1}^{n}j\frac{⟦j\in V\cap L_{t}⟧}{\left|V\cap L_{t}\right|}, \end{array}$$ (13)

$$\begin{array}{c} \nu_{t}=\sum_{j=1}^{n}(j-\mu_{t}{)}^{2}\frac{⟦j\in V\cap L_{t}⟧}{\left|V\cap L_{t}\right|}. \end{array}$$ (14)

Note that the set $V\cap L_{t}$ indicates the valid weeks in $L_{t}$, i.e., outliers are removed for the linear regressions. Also, parameter ℓ in the definition of set $L_{t}$ controls the size of the window in which the regressions are performed: windows have size $|L_{t}|=2\ell +1$ (provided that they are not in the extremities [1,ℓ] and [n−ℓ+1,n]). Similarly to the trend smoothing parameter in STL [33], ℓ should not be too large, to avoid overly smoothing $c_{t}$, nor too small, to prevent short-term fluctuations intended for $s_{t}$ from being absorbed into $c_{t}$. For the weekly sales data in this work, we heuristically select ℓ=26, so that the windows have size 53 weeks, approximately matching the (irregular) periodicity of 1 year, each window approximately having the same types of weeks (see S5 Appendix for alternative values of parameter ℓ).

Then, substituting Eqs (11) and (12) in Eq (9), we obtain:


$$\begin{array}{c} c_{t}^{\left(i\right)}=\sum_{j=1}^{n}\left(1+\frac{\left(t-\mu_{t})(j-\mu_{t}\right)}{\nu_{t}}\right)\frac{⟦j\in V\cap L_{t}⟧}{\left|V\cap L_{t}\right|}y_{j}^{\left(i\right)}. \end{array}$$ (15)

From the additive model definition, iteration i of the seasonal-stochastic component $s_{t}^{\left(i\right)}$ is:


$$\begin{array}{c} s_{t}^{\left(i\right)}=x_{t}-c_{t}^{\left(i\right)}. \end{array}$$ (16)

The next iteration of the seasonally-adjusted time series $y_{t}^{\left(i+1\right)}$ is obtained by removing from the original time series $x_{t}$ the average ${\bar{s}}_{t}^{\left(i\right)}$ of the previous iteration of the seasonal-stochastic component $s_{t}^{\left(i\right)}$ over the corresponding valid analogous weeks in set $A_{t}$ (adjusted by the general level ${\bar{s}}^{\left(i\right)}$ of the seasonal-stochastic component):


$$\begin{array}{cc} y_{t}^{\left(i+1\right)} & =x_{t}-\left(\sum_{j=1}^{n}s_{j}^{\left(i\right)}\frac{⟦j\in V\cap U_{\ell }\cap A_{t}⟧}{\left|V\cap U_{\ell }\cap A_{t}\right|}-\sum_{j=1}^{n}s_{j}^{\left(i\right)}\frac{⟦j\in V\cap U_{\ell }⟧}{\left|V\cap U_{\ell }\right|}\right)\\ & =x_{t}-\sum_{j=1}^{n}s_{j}^{\left(i\right)}\left(\frac{⟦j\in V\cap U_{\ell }\cap A_{t}⟧}{\left|V\cap U_{\ell }\cap A_{t}\right|}-\frac{⟦j\in V\cap U_{\ell }⟧}{\left|V\cap U_{\ell }\right|}\right), \end{array}$$ (17)

and then next iterations $c_{t}^{\left(i+1\right)}$ and $s_{t}^{\left(i+1\right)}$ are calculated from it. This iterative procedure continues until the convergence of the components. Note that the averages are performed over weeks in set $U_{\ell }$, meaning that weeks in the extremities are ignored. This is done because linear regressions in those regions are not computed in symmetric windows, being essentially different from regions not in the extremities (techniques for seasonal adjustment in asymmetric windows exist [61], but they rely on forecasting assumptions regarding the future evolution of the trend-cycle component in order to obtain endpoint estimates [62] – we choose to keep the decomposition and forecasting tasks separate). The proposed algorithm is illustrated in Fig 3.

**Fig 3:**
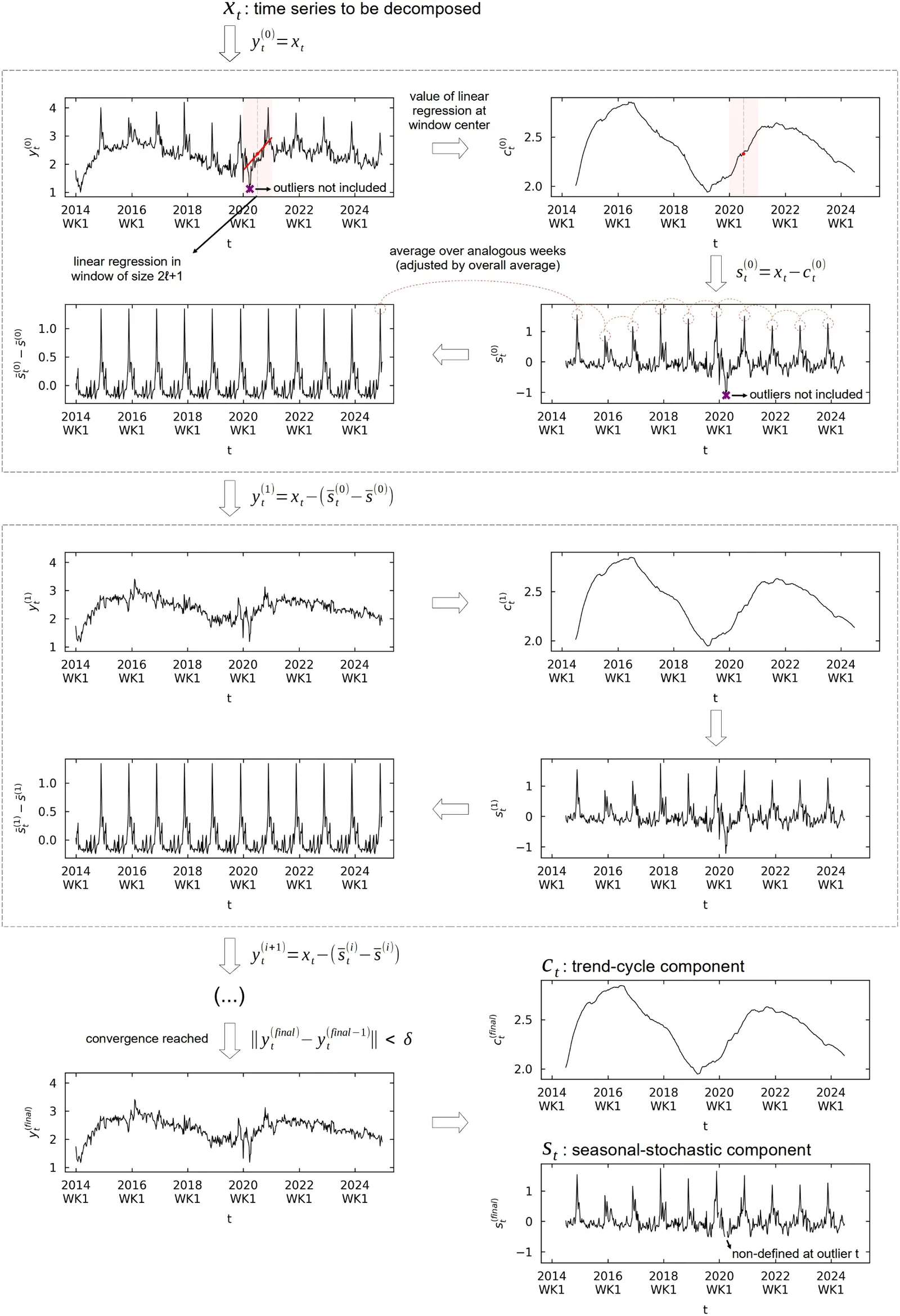
Diagram of the proposed time series decomposition method.

Alternatively, we can use matrix notation for a more systematic study of the proposed decomposition:


$$\begin{array}{c} c^{\left(i\right)}=W_{L}y^{\left(i\right)}, \end{array}$$ (18)

$$\begin{array}{c} s^{\left(i\right)}=x-c^{\left(i\right)}, \end{array}$$ (19)

$$\begin{array}{c} y^{\left(i+1\right)}=x-W_{A}s^{\left(i\right)}, \end{array}$$ (20)

where the matrices elements read as $[x{]}_{t}=x_{t}$, $[c^{\left(i\right)}{]}_{t}=c_{t}^{\left(i\right)}$, $[s^{\left(i\right)}{]}_{t}=s_{t}^{\left(i\right)}$, $[y^{\left(i\right)}{]}_{t}=y_{t}^{\left(i\right)}$, $[W_{L}{]}_{tj}=\left(1+\frac{\left(t-\mu_{t})(j-\mu_{t}\right)}{\nu_{t}}\right)\frac{⟦j\in V\cap L_{t}⟧}{\left|V\cap L_{t}\right|}$ and $[W_{A}{]}_{tj}=\left(\frac{⟦j\in V\cap U_{\ell }\cap A_{t}⟧}{\left|V\cap U_{\ell }\cap A_{t}\right|}-\frac{⟦j\in V\cap U_{\ell }⟧}{\left|V\cap U_{\ell }\right|}\right)$.

We then have:


$$\begin{array}{c} y^{\left(i+1\right)}=(I-W_{A})x+W_{A}W_{L}y^{\left(i\right)}. \end{array}$$ (21)

Iterating from $y^{\left(0\right)}=x$, we arrive at:


$$\begin{array}{c} y^{\left(i\right)}=\left(\left(\sum_{r=0}^{i-1}(W_{A}W_{L}{)}^{r}\right)(I-W_{A})+(W_{A}W_{L}{)}^{i}\right)x. \end{array}$$ (22)

The condition for convergence is the spectral radius of $W_{A}W_{L}$ being strictly less than 1. Assuming such condition (cases for which the convergence condition does not hold remain to be studied):


$$\begin{array}{c} y^{\left(\infty \right)}=(I-W_{A}W_{L}{)}^{-1}(I-W_{A})x. \end{array}$$ (23)

Therefore:


$$\begin{array}{c} c^{\left(\infty \right)}=Wx \end{array}$$ (24)

and


$$\begin{array}{c} s^{\left(\infty \right)}=(I-W)x, \end{array}$$ (25)

with:


$$\begin{array}{c} W=W_{L}(I-W_{A}W_{L}{)}^{-1}(I-W_{A}). \end{array}$$ (26)

The matrix formulation highlights the simplicity of the proposed decomposition, which can be summarized by the linear transformations in Eqs (24) and (25) involving the matrix W in Eq (26). Observe that such matrix does not carry information about the time series $x_{t}$ itself, but only about the calendar (in sets $A_{t}$) and outliers (in set *V*): if multiple time series are affected by the same calendar, then the same matrix can be applied. An additional benefit of this matrix notation is that it provides a condition for convergence that can be verified beforehand for the intended analysis, which is not available, for example, in STL. As an illustration of the structure of matrix W, Fig 4 depicts the 574×574 matrix constructed using the analogous weeks explained in the Analogous weeks subsection (the spectral radius of the corresponding $W_{A}W_{L}$ was 0.019; the largest spectral radius across all decompositions performed in this study was 0.027).

**Fig 4:**
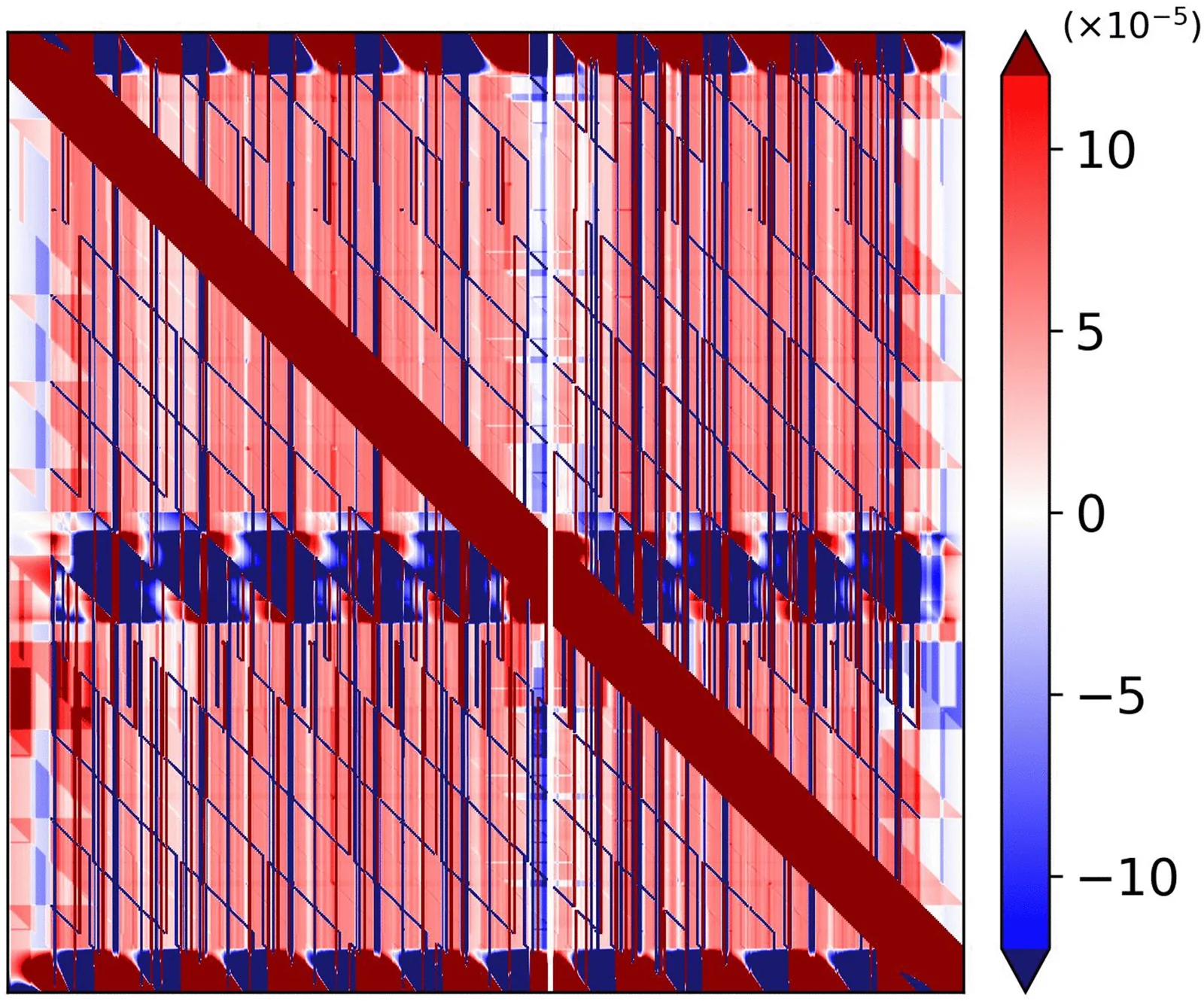
Matrix W to obtain the logarithm of the trend-cycle component of the time series of weekly TV sales from 2014 to 2024 (574 weeks). The matrix has dimension 574×574 and colors represent the values of its coefficients.

We finally define $c_{t}=c_{t}^{\left(\infty \right)}$, $s_{t}=s_{t}^{\left(\infty \right)}$ and $\beta_{t}=\beta_{t}^{\left(\infty \right)}$ computed for $y_{t}^{\left(\infty \right)}$ using Eq (12). Fig 5 shows the decomposition of the weekly TV log-sales time series. Although computed, values of $s_{t}$ at outlier weeks are deemed as non-defined and are not used for forecasting; likewise for values of $c_{t}$ and $s_{t}$ in the extremities.

**Fig 5:**
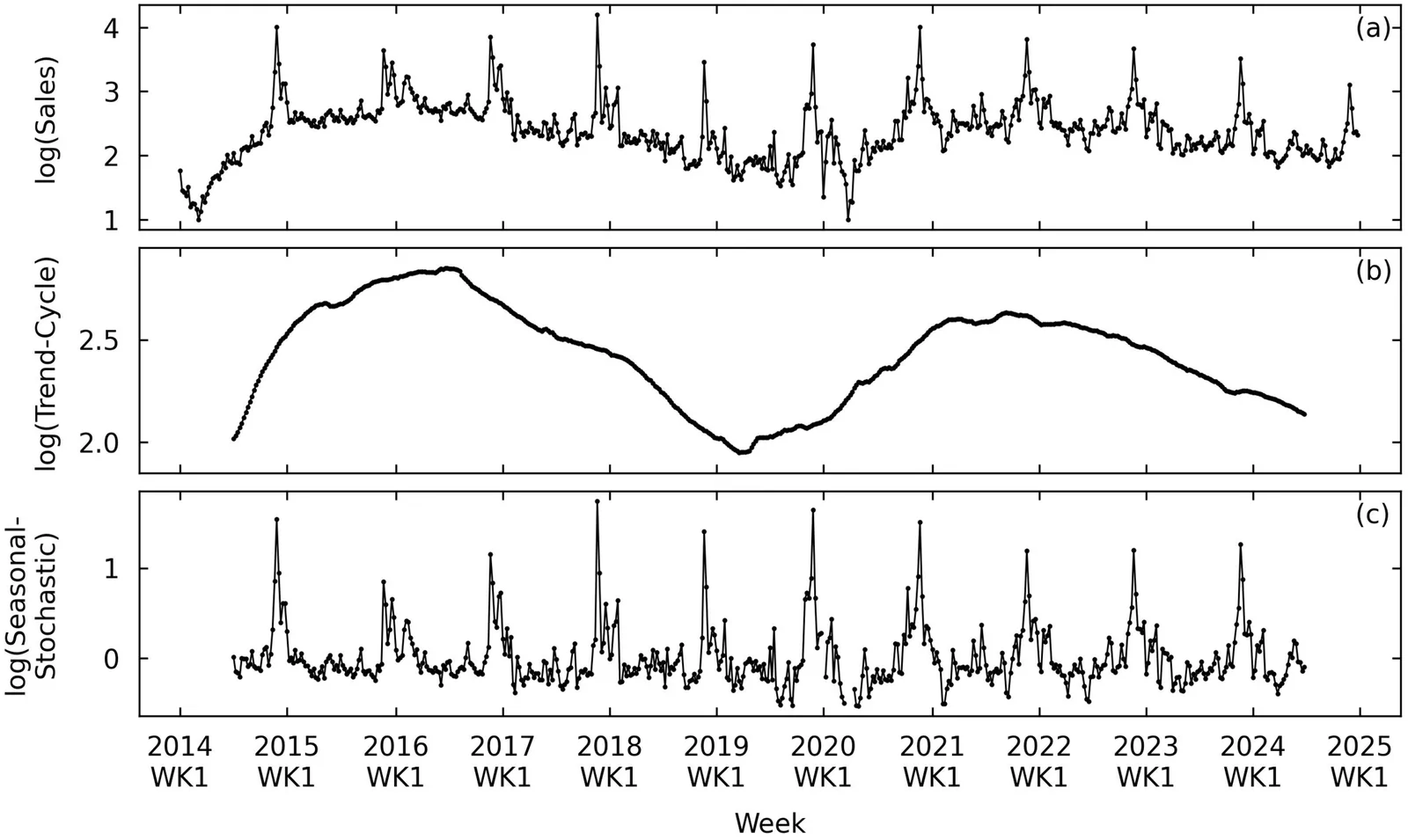
(a) Time series of weekly TV (log-)sales from 2014 to 2024; (b) logarithm of its trend-cycle component; and (c) logarithm of its seasonal-stochastic component.

## Time series forecasting

We forecast the (logarithm of) trend-cycle component $c_{t}$ and the (logarithm of) seasonal-stochastic component $s_{t}$ independently and combine them to obtain a forecast for the time series $x_{t}$ at a desired forecast horizon τ.

### Trend-cycle component $c_{t}$

We consider the following simple linear regression model to forecast the trend-cycle component $c_{t}$:


$$\begin{array}{c} c_{t+\tau }=b_{0}^{\left(\tau \right)}+b_{1}^{\left(\tau \right)}c_{t-\ell }+b_{2}^{\left(\tau \right)}\beta_{t-\ell }+\rho^{\left(\tau \right)}\xi_{t+\tau }, \end{array}$$ (27)

with independent and identically distributed errors ξ~N(0,1). That is, future trend-cycle component value $c_{t+\tau }$ is assumed to be linearly explained by the last available trend-cycle component value $c_{t-\ell }$ and the last available linear tendency represented by the slope $\beta_{t-\ell }$ of the local linear regression in the last available window (see Eq (9)). While linear regression is a standard model, the regressors are constructed from the proposed decomposition.

The model coefficients $b_{0}^{\left(\tau \right)}$, $b_{1}^{\left(\tau \right)}$ and $b_{2}^{\left(\tau \right)}$ are estimated by the ordinary least squares method in set $U_{\ell }$:


$$\begin{array}{c} \hat{b}=(M^{T}M{)}^{-1}M^{T}\gamma , \end{array}$$ (28)

where:


$$\begin{array}{c} {\hat{b}}^{T}=[\begin{array}{ccc} {\hat{b}}_{0}^{\left(\tau \right)} & {\hat{b}}_{1}^{\left(\tau \right)} & {\hat{b}}_{2}^{\left(\tau \right)} \end{array}], \end{array}$$ (29)

$$\begin{array}{c} M=[\begin{array}{ccc} 1 & c_{1+\ell } & \beta_{1+\ell }\\ 1 & c_{1+\ell +1} & \beta_{1+\ell +1}\\ \vdots & \vdots & \vdots \\ 1 & c_{n-\ell -(\ell +\tau )} & \beta_{n-\ell -(\ell +\tau )} \end{array}], \end{array}$$ (30)

$$\begin{array}{c} \gamma^{T}=[\begin{array}{cccc} c_{1+\ell +(\ell +\tau )} & c_{1+\ell +(\ell +\tau )+1} & \ldots & c_{n-\ell } \end{array}]. \end{array}$$ (31)

The forecast trend-cycle component value is then:


$$\begin{array}{c} {\tilde{c}}_{n+\tau }={\hat{b}}_{0}^{\left(\tau \right)}+{\hat{b}}_{1}^{\left(\tau \right)}c_{n-\ell }+{\hat{b}}_{2}^{\left(\tau \right)}\beta_{n-\ell } \end{array}$$ (32)

and its standard deviation is estimated as the standard deviation of the residuals:


$$\begin{array}{cc} \varepsilon_{n+\tau }^{\left(c\right)} & ={\hat{\rho }}^{\left(\tau \right)}\\ & =\sqrt{\frac{\sum_{j=1}^{n}(c_{j+(\ell +\tau )}-({\hat{b}}_{0}^{\left(\tau \right)}+{\hat{b}}_{1}^{\left(\tau \right)}c_{j}+{\hat{b}}_{2}^{\left(\tau \right)}\beta_{j}){)}^{2}⟦j\in U_{\ell }⟧⟦(j+(\ell +\tau ))\in U_{\ell }⟧}{(|U_{\ell }|-(\ell +\tau ))-3}}. \end{array}$$ (33)

### Seasonal-stochastic component $s_{t}$

Consider the average of analogous weeks:


$$\begin{array}{c} {\bar{s}}_{t}=\sum_{j=1}^{n}s_{j}\frac{⟦j\in V\cap U_{\ell }\cap A_{t}⟧}{\left|V\cap U_{\ell }\cap A_{t}\right|} \end{array}$$ (34)

and the associated standard deviation:


$$\begin{array}{c} \sigma_{t}=\sqrt{\sum_{j=1}^{n}(s_{j}-{\bar{s}}_{t}{)}^{2}\frac{⟦j\in V\cap U_{\ell }\cap A_{t}⟧}{|V\cap U_{\ell }\cap A_{t}|-1}} \end{array}$$ (35)

An initial forecast for the seasonal-stochastic component $s_{t}$ would simply be ${\bar{s}}_{t}$. But we can improve it by taking possible serial correlation into account. Since different weeks may have different baselines and fluctuations, we take z-scores:


$$\begin{array}{c} z_{t}=\frac{s_{t}-{\bar{s}}_{t}}{\sigma_{t}}. \end{array}$$ (36)

As one of the simplest processes for modeling serial correlation, we consider an autoregressive model of order 1 for the z-scores (with ξ~N(0,1), i.i.d.):


$$\begin{array}{c} z_{t}=\phi z_{t-1}+\theta \xi_{t}. \end{array}$$ (37)

By recursion, we have:


$$\begin{array}{c} z_{t+\tau }=\phi^{\tau }z_{t}+\theta \sum_{r=0}^{\tau -1}\phi^{r}\xi_{t-r}. \end{array}$$ (38)

Defining the set:


$$\begin{array}{c} U_{\ell }^{\left(\lambda \right)}=[n-\ell -\lambda +1,n-\ell ], \end{array}$$ (39)

with 2≤λ≤n−2ℓ, we can estimate the model parameters by the ordinary least squares methods on valid weeks and interval limited by parameter λ:


$$\begin{array}{c} \hat{\phi }=\frac{\sum_{j=1}^{n}z_{j}z_{j+1}⟦j\in V\cap U_{\ell }^{\left(\lambda \right)}⟧⟦(j+1)\in V\cap U_{\ell }^{\left(\lambda \right)}⟧}{\sum_{j=1}^{n}z_{j}^{2}⟦j\in V\cap U_{\ell }^{\left(\lambda \right)}⟧⟦(j+1)\in V\cap U_{\ell }^{\left(\lambda \right)}⟧}, \end{array}$$ (40)

$$\begin{array}{c} \hat{\theta }=\sqrt{\frac{\sum_{j=1}^{n}(z_{j+1}-\hat{\phi }z_{j}{)}^{2}⟦j\in V\cap U_{\ell }^{\left(\lambda \right)}⟧⟦(j+1)\in V\cap U_{\ell }^{\left(\lambda \right)}⟧}{(|V\cap U_{\ell }^{\left(\lambda \right)}|-1)-1}}. \end{array}$$ (41)

Parameter λ controls how far in the past we look to estimate the serial correlation in the data. We heuristically suggest λ=ℓ, which we use for the TV sales forecasts in this work: λ=ℓ=26 (naturally, it can be treated as a parameter to be optimized).

The forecast z-score is then:


z~n+τ=E(ϕ^τz~n+θ^∑r=0τ−1ϕ^rξt−r|11ϕ^,θ^)=ϕ^τz~n, (42)

where ${\tilde{z}}_{n}$ is given by:


$$\begin{array}{c} {\tilde{z}}_{n}=\frac{(x_{n}-{\tilde{c}}_{n})-{\bar{s}}_{n}}{\sigma_{n}}, \end{array}$$ (43)

with ${\tilde{c}}_{n}$ calculated from Eq (32) for τ=0.

Given the parameter estimates, the standard deviation of the forecast z-score is:


εn+τ(z)=Var(ϕ^τz~n+θ^∑r=0τ−1ϕ^rξt−r|11ϕ^,θ^)=ϕ^2τεn(c)2σn2+θ^21−ϕ^2τ1−ϕ^2, (44)

where we use $Var({\tilde{z}}_{n})=\frac{{\varepsilon_{n}^{\left(c\right)}}^{2}}{\sigma_{n}^{2}}$.

Finally, using Eq (36) to return to the original variable, the forecast seasonal-stochastic component value is:


$$\begin{array}{c} {\tilde{s}}_{n+\tau }={\bar{s}}_{n+\tau }+{\hat{\phi }}^{\tau }{\tilde{z}}_{n}\sigma_{n+\tau }, \end{array}$$ (45)

with standard deviation:


$$\begin{array}{c} \varepsilon_{n+\tau }^{\left(s\right)}=\sigma_{n+\tau }\varepsilon_{n+\tau }^{\left(z\right)}. \end{array}$$ (46)

Note that the autoregressive correction is relevant only for short forecast horizon τ, decaying exponentially with it. We further discuss the autoregressive correction in S6 Appendix.

### Time series $x_{t}$

The models used for the forecasts of $c_{n+\tau }$ and $s_{n+\tau }$ are normal, thus the forecast of $x_{n+\tau }$ is also modeled as normally distributed and it is given by:


$$\begin{array}{c} {\tilde{x}}_{n+\tau }={\tilde{c}}_{n+\tau }+{\tilde{s}}_{n+\tau }. \end{array}$$ (47)

Assuming independence, the standard deviation is:


$$\begin{array}{c} \varepsilon_{n+\tau }^{\left(x\right)}=\sqrt{{\varepsilon_{n+\tau }^{\left(c\right)}}^{2}+{\varepsilon_{n+\tau }^{\left(s\right)}}^{2}}. \end{array}$$ (48)

Returning to the original variable $X_{t}$, we take the point forecast to be:


$$\begin{array}{c} {\tilde{X}}_{n+\tau }=\exp({\tilde{x}}_{n+\tau }) \end{array}$$ (49)

and the prediction interval corresponding to *h* standard deviations of ${\tilde{x}}_{n+\tau }$:


$$\begin{array}{c} \left[\exp({\tilde{x}}_{n+\tau }-h\varepsilon_{n+\tau }^{\left(x\right)}),\exp({\tilde{x}}_{n+\tau }+h\varepsilon_{n+\tau }^{\left(x\right)})\right] \end{array}$$ (50)

Note that while ${\tilde{x}}_{n+\tau }$ is the mean of the normal model, ${\tilde{X}}_{n+\tau }$ is the median (and also the geometric mean) of the corresponding log-normal model. We choose the median as the point forecast because it is obtained directly by back-transforming the forecast on the log scale without requiring an additional retransformation adjustment, in line with our objective of developing a simple method. In contrast, obtaining the arithmetic mean on the original scale requires a bias adjustment when back-transforming the forecast [19,63].

## TV sales forecasting

We now test the performance of the proposed forecasting method applied to the weekly TV sales by using time series cross-validation, also known as rolling origin evaluation (with expanding window) [19,20]. Fig 6 illustrates the procedure: for a given forecast horizon τ, we slide the forecasting origin, i.e., the last past data point used for the forecasting, in order to produce multiple forecasts. Each forecasting origin defines a training set, which contains all available past data up to the origin (black symbols in Fig 6), and a test set, which in our case consists of the single data point τ steps ahead of the origin (red symbols) used to assess the forecast accuracy. Each time the forecasting origin is rolled forward, we obtain a new (expanded) training set, for which the time series components are re-estimated and the forecasting model parameters are refitted; only data in the current training set are used for forecasting, that is, no data beyond the considered origin are included. For each forecast computed in this rolling origin scheme, we have an associated error $E_{i}=A_{i}-F_{i}$, with $A_{i}$ representing the actual value in the corresponding test set and $F_{i}$, the forecast value. Such errors can then be summarized using forecast accuracy metrics.

**Fig 6:**
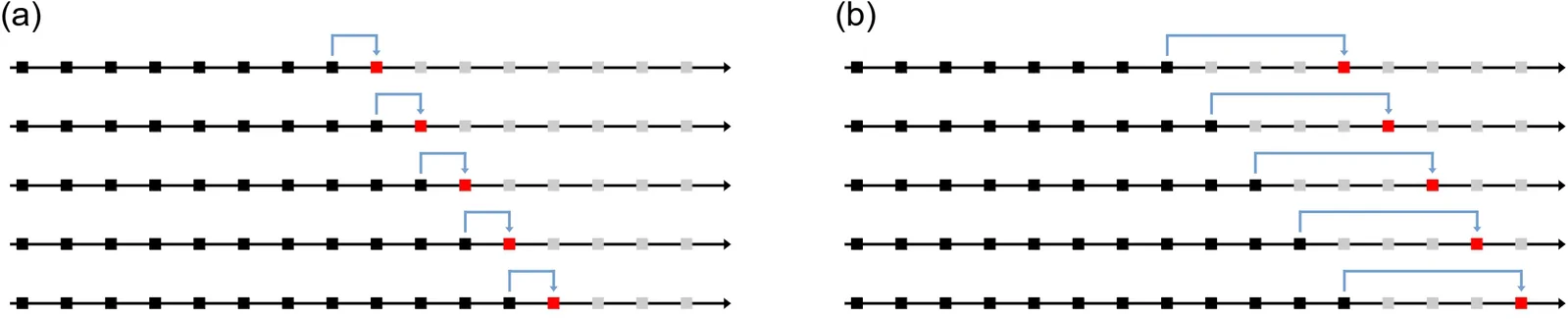
Illustration of rolling origin evaluation for forecast horizon τ of (a) 1 time step and (b) 4 time steps. Black symbols represent past data used for the forecasting (training set) and red symbols represent future data to be compared with the forecasts (test set).

The debate on which point forecast accuracy metric is the most adequate is an ongoing one [64,65]. Avoiding entering the discussion, we select here 5 accuracy metrics frequently used: Mean Absolute Error (MAE), Root Mean Square Error (RMSE), Mean Absolute Percentage Error (MAPE), Weighted Mean Absolute Percentage Error (WMAPE) and Symmetric Mean Absolute Percentage Error (SMAPE).

MAE and RMSE are scale-dependent metrics, having same units as the data:


$$\begin{array}{c} \text{MAE}=\frac{1}{m}\sum_{i=1}^{m}\left|A_{i}-F_{i}\right|, \end{array}$$ (51)

$$\begin{array}{c} \text{RMSE}=\sqrt{\frac{1}{m}\sum_{i=1}^{m}{\left(A_{i}-F_{i}\right)}^{2}}, \end{array}$$ (52)

where *m* is the size of the window for which we are summarizing the errors. RMSE is always larger or equal to MAE and it gives more weight to larger errors. Since the data was masked by dividing it by a factor *q*, the errors are scaled by the same factor. Although the actual sales numbers are unknown, those metrics remain useful for comparing different methods, as the common scaling does not affect the relative magnitude of the errors.

The remaining ones are percentage metrics:


$$\begin{array}{c} \text{MAPE}=\frac{1}{m}\sum_{i=1}^{m}\left|\frac{A_{i}-F_{i}}{A_{i}}\right|, \end{array}$$ (53)

$$\begin{array}{c} \text{WMAPE}=\frac{\sum_{i=1}^{m}\left|A_{i}-F_{i}\right|}{\sum_{i=1}^{m}\left|A_{i}\right|}, \end{array}$$ (54)

$$\begin{array}{c} \text{SMAPE}=\frac{1}{m}\sum_{i=1}^{m}\frac{\left|A_{i}-F_{i}\right|}{(\left|A_{i}\right|+\left|F_{i}\right|)/2}. \end{array}$$ (55)

MAPE is the most commonly used percentage metric, WMAPE and SMAPE being its variants. WMAPE (sometimes called WAPE and also known as MAD/Mean Ratio) weights the percentage errors by the actual values, thus giving more weight to errors of extreme values; since the division in Eq (54) is by the sum of all actual values, it does not have the possible division-by-zero issue of MAPE [66]. SMAPE is always between 0 and 1 and it is symmetric by interchanging $A_{i}$ and $F_{i}$ in Eq (55) [67,68] (although it is questionable if such symmetry represents a significant improvement [69]).

In Fig 7, we show the results of the weekly TV sales forecasting for horizon τ=1 week (using all available data since 2014). Panel (a) shows the sales point forecasts for each week from 2021 to 2024 with the associated prediction intervals of 1.440, 1.645 and 1.960 standard deviations (*h* = 1.440, 1.645 and 1.960 in Eq (50)), respectively corresponding to theoretical coverages of approximately 85%, 90% and 95% under the assumed normal model (two-sided intervals). Panel (b) shows the associated errors $E_{i}$. We observe a particularly large variability in Black Friday weeks, reflecting errors fluctuating on a different scale than in other weeks; with the available data, it is not clear yet if the overprediction is a systematic error of the proposed method or due to the small number of instances (11 Black Friday weeks in total, 4 in the test period), but uncertainty is quantified in the prediction intervals, which are wider for Black Friday weeks than for ordinary weeks. Panels (c) and (d) show the accuracy metrics, each symbol representing the metric calculated in a window containing the past 53 weeks. The scale-dependent metrics lie below 3*q* units and the percentage metrics are essentially below 12%. In S7 Appendix, we compare those results with the forecasting without using analogous weeks, demonstrating that correctly grouping similar weeks is essential for improving accuracy.

**Fig 7:**
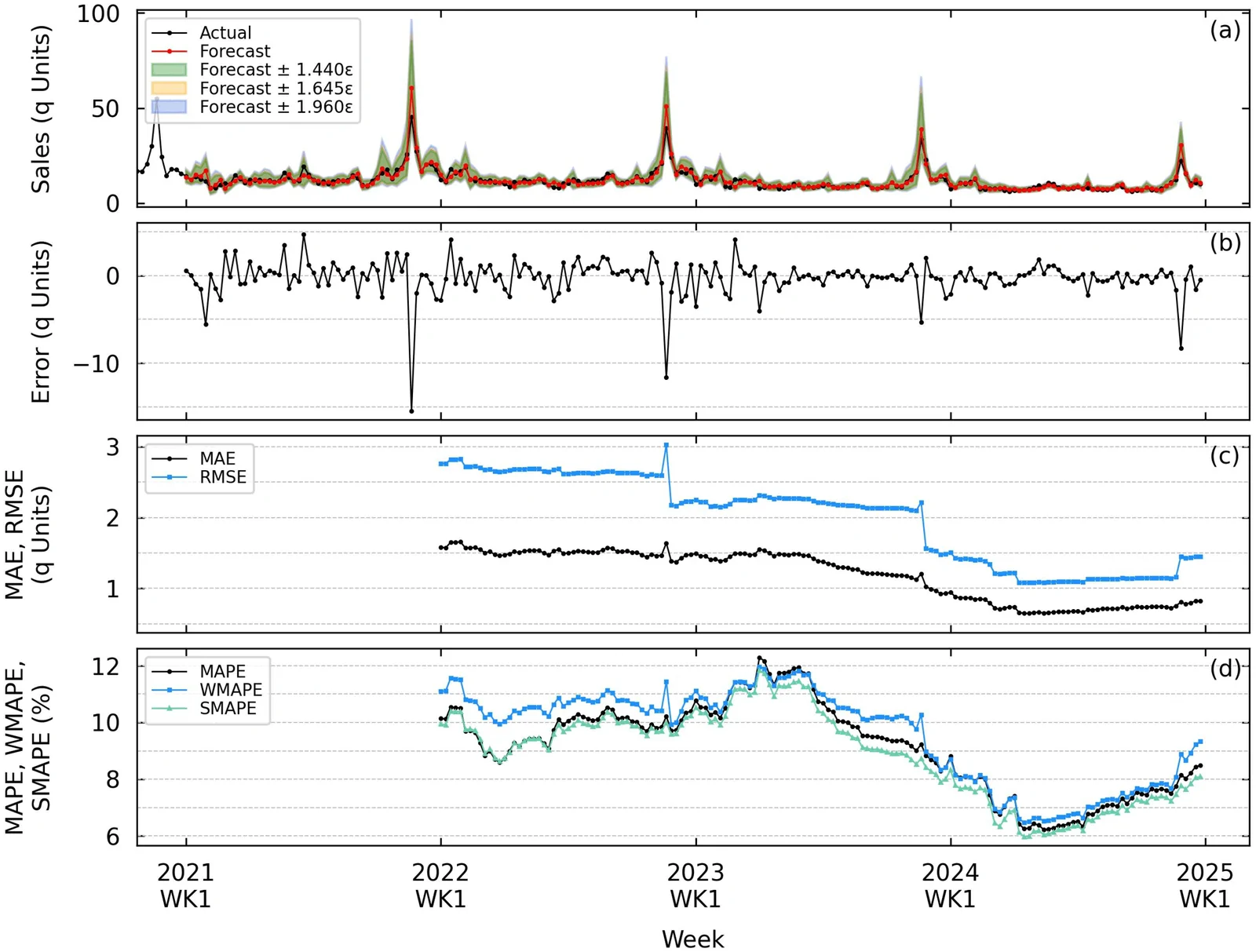
(a) Actual time series of weekly TV sales from 2021 to 2024 (black symbols) and forecasts using horizon of 1 week (red symbols) with prediction intervals of 1.440 (green shade), 1.645 (orange shade) and 1.960 (blue shade) standard deviations; (b) forecast errors; (c) MAE (black circles) and RMSE (light blue squares) computed in windows of 53 weeks; and (d) MAPE (black circles), WMAPE (light blue squares) and SMAPE (light green triangles) computed in windows of 53 weeks.

So as to put the numbers in perspective, we compare ours with other methods. We follow the recommendation to start with appropriate simple benchmarks [19,20]. In Fig 8, we compare the accuracy metrics for forecast horizon τ=1 week of the proposed method with 4 simple benchmarks: Naive Method (last available value as forecast), Analogous Naive Method (last available value from an analogous week as forecast – comparable to Seasonal Naive Method), Average Method (average of all available values as forecast) and Analogous Average Method (average of all available values from analogous weeks as forecast). In all cases, we apply the method to the logarithm of sales and then use the inverse transformation to obtain the final forecast. The proposed method shows superior performance than the simple benchmarks, the latter unable to capture trend and seasonality simultaneously.

**Fig 8:**
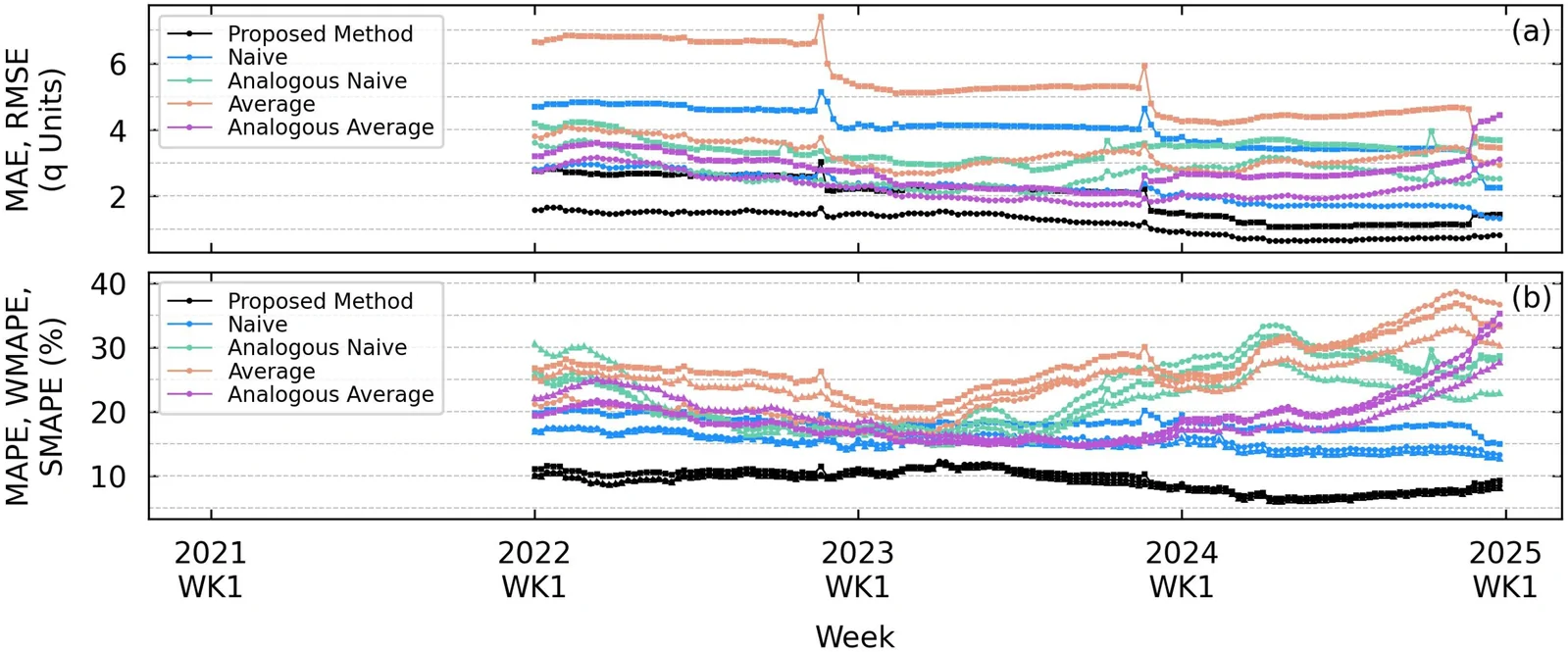
Accuracy metrics computed in windows of 53 weeks for TV sales forecasts using horizon of 1 week from 2021 to 2024: (a) MAE (circles) and RMSE (squares) and (b) MAPE (circles), WMAPE (squares) and SMAPE (triangles) for the proposed method (black symbols), naive method (light blue symbols), analogous naive method (light green symbols), average method (orange symbols) and analogous average method (violet symbols).

We proceed to test our proposed method against two statistical methods that allow the explicit inclusion of information about holidays and events: Prophet and SARIMAX (SARIMA also included for direct comparison with SARIMAX). Those models are commonly used as benchmarks, one of the reasons being the straightforward implementation due to availability of libraries: for Prophet, we use its Python library (version 1.1.4) and the ‘pmdarima’ Python library (version 2.0.4) for the ARIMA models – particularly, the method ‘auto_arima’ for automatic selection of the model orders (autoregressive, differencing and moving-average orders, as well as seasonal orders when applicable) [70,71].

In Fig 9, we compare the accuracy metrics for forecast horizon τ=1 week of the proposed method with the ones obtained with Prophet, SARIMA and SARIMAX models (also applying the log-transformation to sales). For the Prophet procedure, we can include holidays, regular and irregular events in the model specification (outliers are treated as missing values). We consider two cases of Prophet: Default, in which we use the fixed default parameters in order to obtain ‘out-of-the-box’ forecasts, and Fine-Tuned, in which we perform a grid-search for optimum parameters for each forecast using the available past data (we tune the following Prophet parameters: ‘changepoint_range’, ‘changepoint_prior_scale’, ‘seasonality_prior_scale’, ‘holiday_prior_scale’). For SARIMA and SARIMAX, we set the seasonal frequency as 52 weeks, treat irregular events and outliers as missing values and use the presence of holidays and regular events as exogenous variables in SARIMAX (other than that, we use the default parameters). Since there are no default values for the SARIMA and SARIMAX model orders, with their identification being an integral part of the modeling process, the orders are automatically selected and the models are refitted at each forecast origin. Except for Prophet with default parameters, we observe that all other methods display comparable accuracy, with the proposed method and SARIMAX performing the best for the considered forecast horizon. Prophet still allows further tweaks, which provides flexibility and potential to improve performance, although requiring specialized knowledge on the method and increasing the risk of overfitting. We also comment that the automatic selection of model orders in SARIMA and SARIMAX and the fine-tuning in Prophet greatly slow down the procedures, making them computationally expensive. The proposed method is relatively fast, with only 2 parameters to be explicitly selected: parameter ℓ in Eqs (5) and (6) and parameter λ in Eq (39). Similarly to Prophet with default parameters, we use fixed parameters ℓ=λ=26, justified by the (irregular) yearly periodicity of the analyzed data as stated in the Time series decomposition and Time series forecasting sections; sensitivity analyses performed in S5 Appendix and S6 Appendix empirically support those default parameter values and show that the method is robust to moderate variations in them.

**Fig 9:**
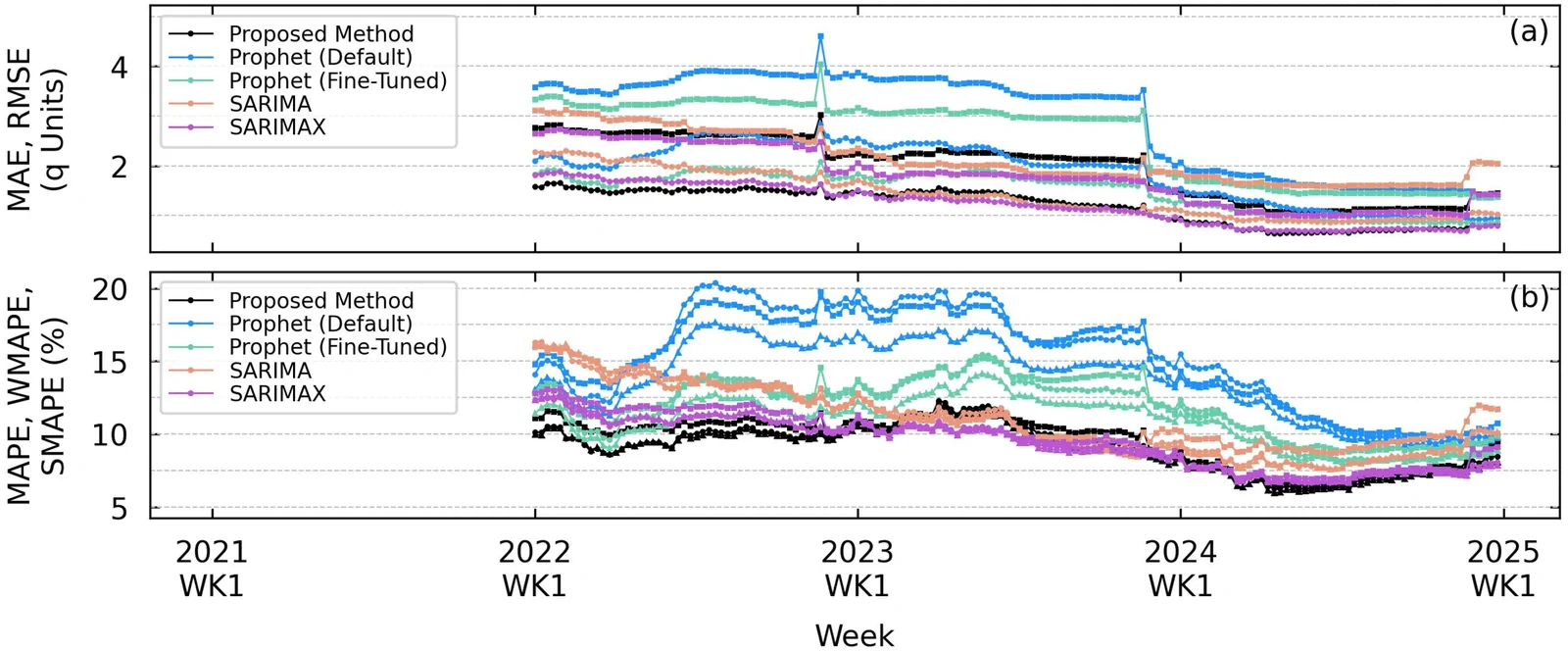
Accuracy metrics computed in windows of 53 weeks for TV sales forecasts using horizon of 1 week from 2021 to 2024: (a) MAE (circles) and RMSE (squares) and (b) MAPE (circles), WMAPE (squares) and SMAPE (triangles) for the proposed method (black symbols), Prophet with default parameters (light blue symbols), Prophet with fine-tuned parameters (light green symbols), SARIMA (orange symbols) and SARIMAX (violet symbols).

Next, we test the methods above for longer forecast horizons. In the same way as in Fig 9, we first compute the accuracy metrics windows of 53 weeks from 2021 to 2024 for horizons τ=1 week (corresponding to Fig 9), τ=4 weeks (~1 month), τ=13 weeks (~3 months) and τ=26 weeks (~6 months). Then, we calculate the averages and standard deviations for each method and horizon. Results are displayed in Table 1 and can be visualized in Fig 10. With exception of RMSE for τ=1, the proposed method shows better accuracy metrics than Prophet, SARIMA and SARIMAX in all accuracy metrics and forecast horizons. While SARIMAX is similar in accuracy to our method for τ=1, its performance deteriorates faster as we increase the horizon, reaching accuracy levels similar to SARIMA and Prophet with fine-tuned parameters for larger τ. Note also that the standard deviations of the accuracy metrics for the proposed method remain low when compared to the other methods, indicating that the accuracy is relatively stable across the evaluated windows.

**Table 1:** Averages of accuracy metrics MAE, RMSE, MAPE, WMAPE and SMAPE computed in windows of 53 weeks from 2021 to 2024 for TV sales forecasts using horizons of 1, 4, 13 and 26 weeks for the proposed method, Prophet with default parameters, Prophet with fine-tuned parameters, SARIMA and SARIMAX (standard deviations in parenthesis).

Method	Horizon (Weeks)	MAE	RMSE	MAPE	WMAPE	SMAPE
Proposed	τ=1	1.194 (0.350)	1.991 (0.614)	0.091 (0.016)	0.096 (0.017)	0.089 (0.016)
	τ=4	1.386 (0.322)	1.922 >(0.442)	0.114 (0.016)	0.113 (0.015)	0.110 (0.015)
	τ=13	1.406 (0.251)	1.850 (0.338)	0.122 (0.016)	0.117 (0.015)	0.117 (0.014)
	τ=26	1.525 (0.207)	1.951 (0.233)	0.137 (0.014)	0.131 (0.013)	0.130 (0.012)
Prophet (Default)	τ=1	1.888 (0.598)	2.941 (0.976)	0.153 (0.036)	0.151 (0.032)	0.137 (0.027)
	τ=4	2.026 (0.670)	3.056 (1.040)	0.167 (0.042)	0.163 (0.037)	0.148 (0.031)
	τ=13	2.462 (0.896)	3.530 (1.244)	0.208 (0.060)	0.199 (0.052)	0.179 (0.044)
	τ=26	3.495 (1.365)	4.762 (1.783)	0.295 (0.091)	0.288 (0.086)	0.243 (0.067)
Prophet (Fine-Tuned)	τ=1	1.492 (0.402)	2.567 (0.798)	0.117 (0.021)	0.120 (0.020)	0.109 (0.017)
	τ=4	1.646 (0.474)	2.666 (0.852)	0.131 (0.025)	0.133 (0.024)	0.121 (0.020)
	τ=13	1.808 (0.574)	2.879 (0.928)	0.147 (0.034)	0.147 (0.030)	0.132 (0.026)
	τ=26	2.693 (1.129)	4.033 (1.568)	0.221 (0.074)	0.220 (0.066)	0.189 (0.054)
SARIMA	τ=1	1.415 (0.459)	2.147 (0.499)	0.109 (0.024)	0.114 (0.021)	0.108 (0.025)
	τ=4	1.949 (0.465)	2.846 (0.501)	0.155 (0.022)	0.159 (0.016)	0.152 (0.021)
	τ=13	2.036 (0.670)	2.640 (0.672)	0.178 (0.037)	0.166 (0.033)	0.165 (0.033)
	τ=26	2.108 (0.538)	2.885 (0.716)	0.189 (0.033)	0.177 (0.023)	0.167 (0.023)
SARIMAX	τ=1	1.216 (0.396)	1.794 (0.580)	0.094 (0.017)	0.097 (0.018)	0.093 (0.017)
	τ=4	1.589 (0.344)	1.963 (0.384)	0.139 (0.014)	0.130 (0.011)	0.135 (0.014)
	τ=13	1.971 (0.455)	2.421 (0.492)	0.176 (0.018)	0.163 (0.018)	0.165 (0.018)
	τ=26	2.151 (0.341)	2.668 (0.448)	0.203 (0.023)	0.183 (0.018)	0.182 (0.019)

**Fig 10:**
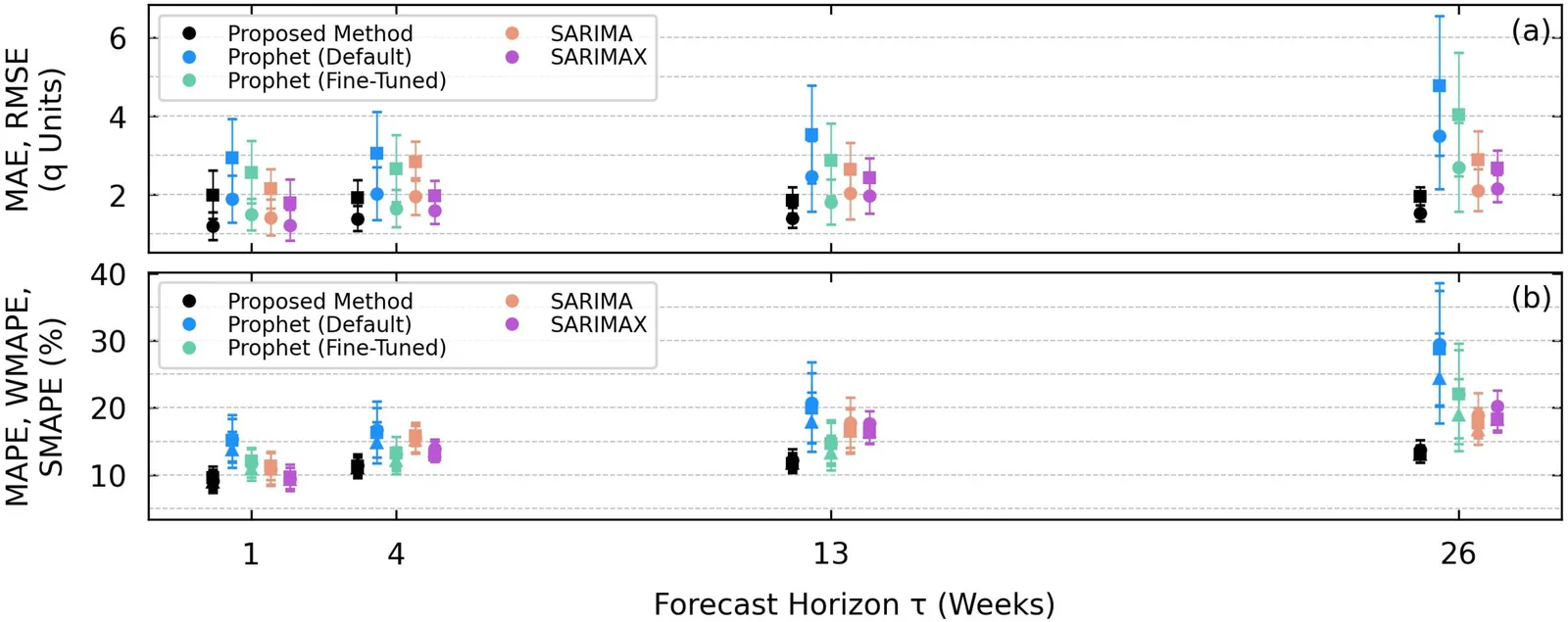
Averages and standard deviations of accuracy metrics computed in windows of 53 weeks from 2021 to 2024 for TV sales forecasts using horizons of 1, 4, 13 and 26 weeks: (a) MAE (circles) and RMSE (squares) and (b) MAPE (circles), WMAPE (squares) and SMAPE (triangles) for the proposed method (black symbols), Prophet with default parameters (light blue symbols), Prophet with fine-tuned parameters (light green symbols), SARIMA (orange symbols) and SARIMAX (violet symbols).

The point forecast accuracy metrics used above are useful to quantify the magnitude of forecast errors and provide a direct measure of practical accuracy differences between methods, but they do not assess if such differences are statistically meaningful or could arise from sampling variability. In order to determine whether the differences in observed forecast accuracy between competing methods are statistically significant, we employ the Diebold-Mariano (DM) test (with the Harvey-Leybourne-Newbold small-sample correction) [72,73]. The DM test takes the form of a hypothesis test, where the null hypothesis states that the competing forecasts have equal expected loss, i.e., there is no difference in predictive accuracy. The test is based on the difference between the losses of the two forecasts for each forecast instance. We consider here two loss functions: Absolute Error (AE), $\left|A_{i}-F_{i}\right|$, and Squared Error (SE), ${\left(A_{i}-F_{i}\right)}^{2}$; similarly to RMSE, the SE loss function places more weight on larger errors due to squaring. DM test results are shown in Table 2. As we set the proposed method as the reference method, negative values of the DM statistics indicate that its forecasts have lower average loss than the compared method, while p-values below a significance level support the rejection of the null hypothesis of no difference in predictive accuracy. We first observe that the DM statistics are negative in almost all cases, with the exception of SARIMAX for τ=1 under the SE loss function, consistent with the related metrics MAE and RMSE in Table 1. Using a significance level of 0.05, the proposed method exhibits significantly lower losses than Prophet with default parameters and SARIMA for all horizons except SARIMA at τ=1 under the SE loss function. For Prophet with fine-tuned parameters, results are mixed, with significant differences observed for τ=1 and τ=26 but not for the intermediate horizons, suggesting that the effectiveness of the fine-tuning strategy may depend on the forecast horizon. For SARIMAX, no significant differences are found for short-term forecasts (τ=1 and τ=4), while significant differences are observed for longer horizons (τ=13 and τ=26). Overall, these results show that the competitive performance of the proposed method in this case study is not only reflected in lower error metrics but is also statistically supported in most comparisons (significantly superior in 23 out of 32 comparisons at significance level of 0.05). The benchmarks Prophet and SARIMAX produce forecasts with similar accuracy to the proposed method under specific conditions: when Prophet parameters are effectively fine-tuned and when SARIMAX is applied to short forecast horizons.

**Table 2:** DM test results comparing forecast accuracy between the proposed method and Prophet with default parameters, Prophet with fine-tuned parameters, SARIMA and SARIMAX for TV sales forecasts from 2021 to 2024 using horizons of 1, 4, 13 and 26 weeks. DM statistics and corresponding p-values are computed for AE and SE loss functions.

Method	Horizon (Weeks)	DM Statistic (AE Loss)	DM Statistic (SE Loss)	p-value (AE Loss)	p-value (SE Loss)
Prophet (Default)	τ=1	−5.691	−3.034	< 10^–7^	0.003
	τ=4	−2.480	−2.080	0.014	0.039
	τ=13	−2.390	−2.287	0.018	0.023
	τ=26	−2.716	−2.214	0.007	0.028
Prophet (Fine-Tuned)	τ=1	−2.990	−2.053	0.003	0.041
	τ=4	−1.286	−1.530	0.200	0.128
	τ=13	−1.581	−1.794	0.116	0.074
	τ=26	−2.213	−2.293	0.028	0.023
SARIMA	τ=1	−3.521	−1.541	< 10^–3^	0.125
	τ=4	−3.968	−3.375	< 10^–3^	< 10^–3^
	τ=13	−2.788	−2.942	0.006	0.004
	τ=26	−2.608	−2.181	0.010	0.031
SARIMAX	τ=1	−0.707	0.720	0.480	0.473
	τ=4	−1.573	−0.155	0.117	0.877
	τ=13	−3.781	−3.445	< 10^–3^	< 10^–3^
	τ=26	−4.825	−5.270	< 10^–5^	< 10^–6^

Lastly, we comment on the prediction intervals produced by the proposed forecasting model. We focus on two metrics: empirical coverage and average width. The empirical coverage is calculated as the proportion of actual values that fall within the considered set of prediction intervals, indicating how reliable the intervals are in capturing the expected uncertainty. The average width is the average of the intervals widths (upper bound minus lower bound), reflecting the precision of the forecasts: narrower intervals imply higher precision. Results for prediction intervals with theoretical coverages of 85%, 90% and 95% (under the normal model assumption) are shown in Table 3, also from 2021 to 2024 for horizons τ=1, 4, 13 and 26 weeks. Besides showing the metrics for all weeks in the analyzed period, we also separate them into ordinary weeks and special weeks (weeks with holidays/events, gap weeks and last weeks). We first observe that empirical coverages are generally consistent with the theoretical ones, except for the longer horizon τ=26, for which the intervals tend to be conservative (overcoverage). Secondly, as appropriate, interval widths are wider for longer horizons (behavior controlled by the error of trend-cycle component in Eq (33)) and also wider for special weeks (controlled by the error of seasonal-stochastic component in Eq (46)), which is apparent in Fig 7-(a), e.g., for weeks related to Super Bowl, Black Friday or Christmas. In S8 Appendix, we provide additional discussion on the (log-)normality assumption and the calibration of the prediction intervals, including a study suggesting that the overcoverage observed for τ=26 is associated with an overestimation of the standard deviation of the trend-cycle component, highlighting that improvements in trend-cycle forecasting are needed for better estimation of the prediction intervals.

**Table 3:** Empirical coverages and average widths of prediction intervals corresponding to theoretical coverages of 85%, 90% and 95% for TV sales forecasts from 2021 to 2024 using horizons of 1, 4, 13 and 26 weeks for the proposed method (standard deviations in parenthesis). Results are presented for all weeks and separately for ordinary and special weeks.

Theoretical Coverage	Horizon (Weeks)	Empirical Coverage (All)	Empirical Coverage (Ordinary)	Empirical Coverage (Special)	Average Width (All)	Average Width (Ordinary)	Average Width (Special)
85%	τ=1	0.875	0.875	0.875	5.435 (4.893)	3.936 (1.863)	7.832 (6.906)
	τ=4	0.849	0.856	0.838	6.139 (5.434)	4.561 (2.647)	8.605 (7.427)
	τ=13	0.852	0.858	0.842	6.582 (5.428)	4.931 (1.633)	9.189 (7.818)
	τ=26	0.923	0.927	0.918	7.907 (6.392)	5.693 (1.513)	11.243 (9.002)
90%	τ=1	0.899	0.906	0.887	6.232 (5.632)	4.509 (2.146)	8.990 (7.950)
	τ=4	0.878	0.872	0.887	7.049 (6.275)	5.231 (3.068)	9.890 (8.573)
	τ=13	0.903	0.900	0.908	7.557 (6.278)	5.652 (1.881)	10.566 (9.049)
	τ=26	0.940	0.955	0.918	9.096 (7.418)	6.532 (1.747)	12.959 (10.454)
95%	τ=1	0.952	0.945	0.963	7.477 (6.802)	5.399 (2.594)	10.802 (9.602)
	τ=4	0.937	0.936	0.938	8.477 (7.622)	6.278 (3.751)	11.914 (10.408)
	τ=13	0.954	0.967	0.934	9.088 (7.648)	6.775 (2.273)	12.740 (11.036)
	τ=26	0.962	0.982	0.932	10.974 (9.092)	7.843 (2.124)	15.692 (12.829)

## Conclusion

We developed a forecasting method with the constraints of simplicity and interpretability, in the sense that practitioners without extensive mathematical/statistical training can comprehend how the forecast was built (and, if needed, adapt the method to their purposes). It is based on decomposing a time series into a trend-cycle component and a seasonal-stochastic component. Each component is forecasted independently – using linear regression for the trend-cycle and a simple average with autoregressive correction for the seasonal-stochastic component – and then recombined to obtain the final forecast. Despite its simplicity, it achieved comparable performance to or outperformed standard models in the task of forecasting weekly TV sales in our particular case study.

The main idea we introduced to improve accuracy when modeling weekly data was the concept of ‘analogous weeks’, incorporated both in the time series decomposition and forecasting. It provided a framework to organize the weeks in a year, with or without holidays/events, dealing at the same time with irregular seasonality and calendar effects. Naturally, this notion can be extended to other time units, but the rules to define the analogies need to be carefully defined in order to reflect the observed behavior in the time series. For example, if working with ‘analogous days’, weekly seasonality may play a significant role and it is then important to take the day of the week in consideration, e.g., the set of analogous days of a given Monday should only contain Mondays.

The proposed simple method achieved high accuracy for the considered sales data, which, despite the irregular seasonality, is relatively well-behaved. However, no forecasting method consistently dominates across all datasets and the most appropriate approach depends on the characteristics of the data and forecasting problem [17,18]. Therefore, we anticipate that some sophistication may be needed when handling datasets with more complex features such as intermittency, structural breaks or large deviations from the (log-)normality assumption. As the ‘analogous weeks’ concept is not specifically tied to the procedure we developed, a promising direction for future work is to integrate the ‘analogous [time unit]’ framework into already established fixed seasonality methods, either more refined decomposition techniques such as the STL or models such as ARIMA, creating, for example, a variation of SARIMA that can deal with irregular seasonality.

## Supporting information

S1 Appendix Data masking. (PDF)

S2 Appendix COVID-19 outliers. (PDF)

S3 Appendix Analogous weeks of irregular events. (PDF)

S4 Appendix Holiday/event overlap. (PDF)

S5 AppendixParameter ℓ of time series decomposition.(PDF)

S6 AppendixAutoregressive correction for $s_{t}$ forecasting.(PDF)

S7 Appendix Forecasting without analogous weeks. (PDF)

S8 Appendix (Log-)Normality assumption. (PDF)
