# Supplementary material for: Weekly sales forecasting using time series decomposition with irregular seasonality: A case study of Sony TV sales in the United States
Source: PLoS One. 2026 Nov 3;21(11):e0344222. doi: 10.1371/journal.pone.0344222 (PMC13730381; doi:10.1371/journal.pone.0344222)
Supplement: Supplementary file 1 [file pone.0344222.s001.pdf]

Weekly sales forecasting using time series decomposition with irregular seasonality: A case study of Sony TV sales in the United States

Arthur Matsuo Yamashita Rios de Sousa, Daisuke Ando, Junichi Ozaki, Yohei Shida, Qianyun Wu, Yixuan Y. Zheng, Zhihua Zhong, Rie Maskawa, Jiwei J. Jiang, Kay Nagayama, Hideki Takayasu, Misako Takayasu

S1 Appendix: Data masking

The data used in this work was masked by dividing the actual sales numbers by a constant scaling factor q, similar to a change in measurement units (e.g., from inches to centimeters). Such data masking does not affect the forecast accuracy metrics or the conclusions of the study, which can be straightforwardly verified by rescaling the publicly provided dataset by different factors. Table S1-1 shows the forecasting results for different multiplying factors r applied to the masked data, corresponding to effective scaling factors q/r; the results remain essentially unchanged.

Table S1-1. Averages of accuracy metrics MAE, RMSE, MAPE, WMAPE and SMAPE computed in windows of 53 weeks from 2021 to 2024 for TV sales forecasts using horizons of 1, 4, 13 and 26 weeks for different multiplying factors for the data masking (standard deviations in parenthesis).

Multiplying factor r	Horizon (Weeks)	MAE/r	RMSE/r	MAPE	WMAPE	SMAPE
$r = 0.169$	$\tau = 1$	1.194 (0.350)	1.991 (0.614)	0.091 (0.016)	0.096 (0.017)	0.089 (0.016)
	$\tau = 4$	1.386 (0.322)	1.922 (0.442)	0.114 (0.016)	0.113 (0.015)	0.110 (0.015)
	$\tau = 13$	1.406 (0.251)	1.850 (0.338)	0.122 (0.016)	0.117 (0.015)	0.117 (0.014)
	$\tau = 26$	1.525 (0.207)	1.951 (0.233)	0.137 (0.014)	0.131 (0.013)	0.130 (0.012)
$r = 23.18$	$\tau = 1$	1.194 (0.350)	1.991 (0.614)	0.091 (0.016)	0.096 (0.017)	0.089 (0.016)
	$\tau = 4$	1.386 (0.322)	1.922 (0.442)	0.114 (0.016)	0.113 (0.015)	0.110 (0.015)
	$\tau = 13$	1.406 (0.251)	1.850 (0.338)	0.122 (0.016)	0.117 (0.015)	0.117 (0.014)
	$\tau = 26$	1.525 (0.206)	1.951 (0.233)	0.137 (0.014)	0.131 (0.013)	0.130 (0.012)
$r = 5127$	$\tau = 1$	1.194 (0.350)	1.991 (0.614)	0.091 (0.016)	0.096 (0.017)	0.089 (0.016)
	$\tau = 4$	1.386 (0.322)	1.922 (0.442)	0.114 (0.016)	0.113 (0.015)	0.110 (0.015)
	$\tau = 13$	1.406 (0.251)	1.850 (0.338)	0.122 (0.016)	0.117 (0.015)	0.117 (0.014)
	$\tau = 26$	1.525 (0.206)	1.951 (0.233)	0.137 (0.014)	0.131 (0.013)	0.130 (0.012)
