# Supplementary material for: Weekly sales forecasting using time series decomposition with irregular seasonality: A case study of Sony TV sales in the United States
Source: PLoS One. 2026 Nov 3;21(11):e0344222. doi: 10.1371/journal.pone.0344222 (PMC13730381; doi:10.1371/journal.pone.0344222)
Supplement: Supplementary file 2 [file pone.0344222.s002.pdf]

Weekly sales forecasting using time series decomposition with irregular seasonality: A case study of Sony TV sales in the United States

Arthur Matsuo Yamashita Rios de Sousa, Daisuke Ando, Junichi Ozaki, Yohei Shida, Qianyun Wu, Yixuan Y. Zheng, Zhihua Zhong, Rie Maskawa, Jiwei J. Jiang, Kay Nagayama, Hideki Takayasu, Misako Takayasu

S2 Appendix: COVID-19 outliers

In this study, weeks identified as outliers were directly informed by Sony, each corresponding to a known disruption in production or supply. The most prominent outliers during the study period are those associated with the COVID-19 pandemic outbreak in 2020, from week 2020-WK12 to week 2020-WK15 (4 weeks). Since the effects of this extraordinary event may have persisted beyond the informed weeks, we check here the robustness of our analysis by extending the assumed period for the COVID outliers.

We perform forecasting for horizon $\tau = 1$ week assuming extended periods for the COVID outliers: 2020-WK12 to 2020-WK19 (8 weeks), 2020-WK12 to 2020-WK23 (12 weeks) and 2020-WK12 to 2020-WK27 (16 weeks). The results are shown in Table S2-1. We observe that forecast accuracy is robust to moderate variations in the COVID outliers interval. Removing an excessively long outlier period, however, seems to degrade forecasting performance, likely because the local linear regression in the time series decomposition becomes unstable when too many observations are omitted from the corresponding estimation window. Although lingering effects may exist, the results suggest that extending the outlier period beyond the initial shock of the COVID-19 outbreak does not significantly improve forecasting performance and the information on COVID outliers provided by Sony appears to be adequate for the proposed forecasting.

Table S2-1. Averages of accuracy metrics MAE, RMSE, MAPE, WMAPE and SMAPE computed in windows of 53 weeks from 2021 to 2024 for TV sales forecasts using horizon $\tau = 1$ week under different assumed periods for the COVID outliers (standard deviations in parenthesis).

COVID Outliers	MAE	RMSE	MAPE	WMAPE	SMAPE
2020WK12 ∼ 2020WK15 (4 weeks)	1.194 (0.350)	1.991 (0.614)	0.091 (0.016)	0.096 (0.017)	0.089 (0.016)
2020WK12 ∼ 2020WK19 (8 weeks)	1.193 (0.341)	1.983 (0.602)	0.091 (0.016)	0.096 (0.016)	0.088 (0.015)
2020WK12 ∼ 2020WK23 (12 weeks)	1.212 (0.349)	1.988 (0.602)	0.093 (0.016)	0.097 (0.016)	0.090 (0.016)
2020WK12 ∼ 2020WK27 (16 weeks)	1.232 (0.360)	2.005 (0.615)	0.095 (0.017)	0.099 (0.017)	0.092 (0.017)
