# Supplementary material for: Weekly sales forecasting using time series decomposition with irregular seasonality: A case study of Sony TV sales in the United States
Source: PLoS One. 2026 Nov 3;21(11):e0344222. doi: 10.1371/journal.pone.0344222 (PMC13730381; doi:10.1371/journal.pone.0344222)
Supplement: Supplementary file 3 [file pone.0344222.s003.pdf]

Weekly sales forecasting using time series decomposition with irregular seasonality: A case study of Sony TV sales in the United States

Arthur Matsuo Yamashita Rios de Sousa, Daisuke Ando, Junichi Ozaki, Yohei Shida, Qianyun Wu, Yixuan Y. Zheng, Zhihua Zhong, Rie Maskawa, Jiwei J. Jiang, Kay Nagayama, Hideki Takayasu, Misako Takayasu

S3 Appendix: Analogous weeks of irregular events

The selection of analogous weeks for irregular events requires forecaster judgment. Here, we assess the sensitivity of the forecasting results to alternative judgments. In the main text, we use an informed selection based on similarity in event characteristics or temporal proximity for the irregular events COVID-19 Economic Impact Payments (analogous to Memorial Day), Amazon Prime Day 2 (analogous to Amazon Prime Day) and Best Buy Early Black Friday Deals (analogous to Amazon Prime Day). Table S3-1 presents the forecasting results for horizon $\tau = 1$ week obtained under alternative selections in which all irregular events are treated as analogous to either Amazon Prime Day, Memorial Day, Easter or Black Friday. While errors are the lowest for the informed selection, accuracy is not substantially affected under reasonable alternative settings (e.g., excluding events with distinct sales behavior, such as Easter, and clearly unrealistic assignments, such as treating Black Friday as a similar event).

Table S3-1. Averages of accuracy metrics MAE, RMSE, MAPE, WMAPE and SMAPE computed in windows of 53 weeks from 2021 to 2024 for TV sales forecasts using horizon $\tau = 1$ week for alternative selections of analogous weeks for irregular events (standard deviations in parenthesis).

Irregular Events Analogous Weeks	MAE	RMSE	MAPE	WMAPE	SMAPE
Informed Selection	1.194<br>(0.350)	1.991<br>(0.614)	0.091<br>(0.016)	0.096<br>(0.017)	0.089<br>(0.016)
Amazon Prime Day	1.198<br>(0.353)	2.019<br>(0.644)	0.092<br>(0.016)	0.096<br>(0.017)	0.090<br>(0.016)
Memorial Day	1.265<br>(0.373)	2.072<br>(0.654)	0.097<br>(0.016)	0.102<br>(0.017)	0.094<br>(0.016)
Easter	1.321<br>(0.399)	2.139<br>(0.673)	0.101<br>(0.017)	0.106<br>(0.018)	0.098<br>(0.017)
Black Friday	2.007<br>(0.585)	5.506<br>(1.445)	0.157<br>(0.024)	0.162<br>(0.026)	0.123<br>(0.018)
