# Supplementary material for: Weekly sales forecasting using time series decomposition with irregular seasonality: A case study of Sony TV sales in the United States
Source: PLoS One. 2026 Nov 3;21(11):e0344222. doi: 10.1371/journal.pone.0344222 (PMC13730381; doi:10.1371/journal.pone.0344222)
Supplement: Supplementary file 4 [file pone.0344222.s004.pdf]

Weekly sales forecasting using time series decomposition with irregular seasonality: A case study of Sony TV sales in the United States

Arthur Matsuo Yamashita Rios de Sousa, Daisuke Ando, Junichi Ozaki, Yohei Shida, Qianyun Wu, Yixuan Y. Zheng, Zhihua Zhong, Rie Maskawa, Jiwei J. Jiang, Kay Nagayama, Hideki Takayasu, Misako Takayasu

S4 Appendix: Holiday/event overlap

When holidays or events overlap in a given week, forecaster judgment is needed to decide the dominant event, i.e., the set of analogous weeks to which the week will be assigned. In the period analyzed in this work, there is one instance of overlap: the week before Independence Day week and Amazon Prime Day week in 2021, with Amazon Prime Day judged to be the dominant event in the main text. Here, we test how sensitive are the forecasting results to the opposite judgment. Table S4-1 shows the forecasting results for horizon $\tau = 1$ week for the two possible choices of dominant event: Amazon Prime Day or the week before Independence Day. The results indicate little sensitivity to the choice of dominant event in this case. A more pronounced effect would be expected if the overlap involved events with substantially different sales volumes, such as Black Friday and a minor holiday/event.

Table S4-1. Averages of accuracy metrics MAE, RMSE, MAPE, WMAPE and SMAPE computed in windows of 53 weeks from 2021 to 2024 for TV sales forecasts using horizon $\tau = 1$ week for alternative choices of dominant event when overlap occurs (standard deviations in parenthesis).

Dominant Event	MAE	RMSE	MAPE	WMAPE	SMAPE
Amazon Prime Day	1.194<br>(0.350)	1.991<br>(0.614)	0.091<br>(0.016)	0.096<br>(0.017)	0.089<br>(0.016)
Independence Day (Week Before)	1.214<br>(0.367)	2.026<br>(0.654)	0.093<br>(0.017)	0.097<br>(0.018)	0.091<br>(0.017)
