# Supplementary material for: Weekly sales forecasting using time series decomposition with irregular seasonality: A case study of Sony TV sales in the United States
Source: PLoS One. 2026 Nov 3;21(11):e0344222. doi: 10.1371/journal.pone.0344222 (PMC13730381; doi:10.1371/journal.pone.0344222)
Supplement: Supplementary file 5 [file pone.0344222.s005.pdf]

Weekly sales forecasting using time series decomposition with irregular seasonality: A case study of Sony TV sales in the United States

Arthur Matsuo Yamashita Rios de Sousa, Daisuke Ando, Junichi Ozaki, Yohei Shida, Qianyun Wu, Yixuan Y. Zheng, Zhihua Zhong, Rie Maskawa, Jiwei J. Jiang, Kay Nagayama, Hideki Takayasu, Misako Takayasu

S5 Appendix: Parameter ℓ of time series decomposition

Parameter ℓ in the proposed time series decomposition controls the size of the window over which the local linear regressions are performed. If ℓ is too small, short-term fluctuations that should be captured by the seasonal-stochastic component s_t are instead absorbed into the trend-cycle component c_t (see Figure S5-1). Conversely, if ℓ is too large, c_t may become overly smoothed and longer segments at the beginning and end of the time series have undefined component estimates (see Figure S5-2). In the main text, we heuristically select $\ell = 26$, so that the windows have size 53 weeks, approximately matching the (irregular) periodicity of 1 year.

We perform a sensitivity analysis across a range of values of parameter ℓ. For the additional parameter λ, which is used in the autoregressive correction for s_t, we either use the suggested rule $\lambda = \ell$ or keep it fixed at $\lambda = 26$. Table S5-1 reports the forecasting results for horizon $\tau = 1$ week for the combinations of parameters ℓ and λ. The results support the heuristic choice of $\ell = 26$ as a practical default and indicate that the method is robust to moderate variations in ℓ around this value, with forecasting performance slightly deteriorating for very small or very large values of ℓ. The choice between $\lambda = \ell$ and $\lambda = 26$ generally has a limited effect on accuracy; a more detailed investigation of parameter λ is provided in S6 Appendix.

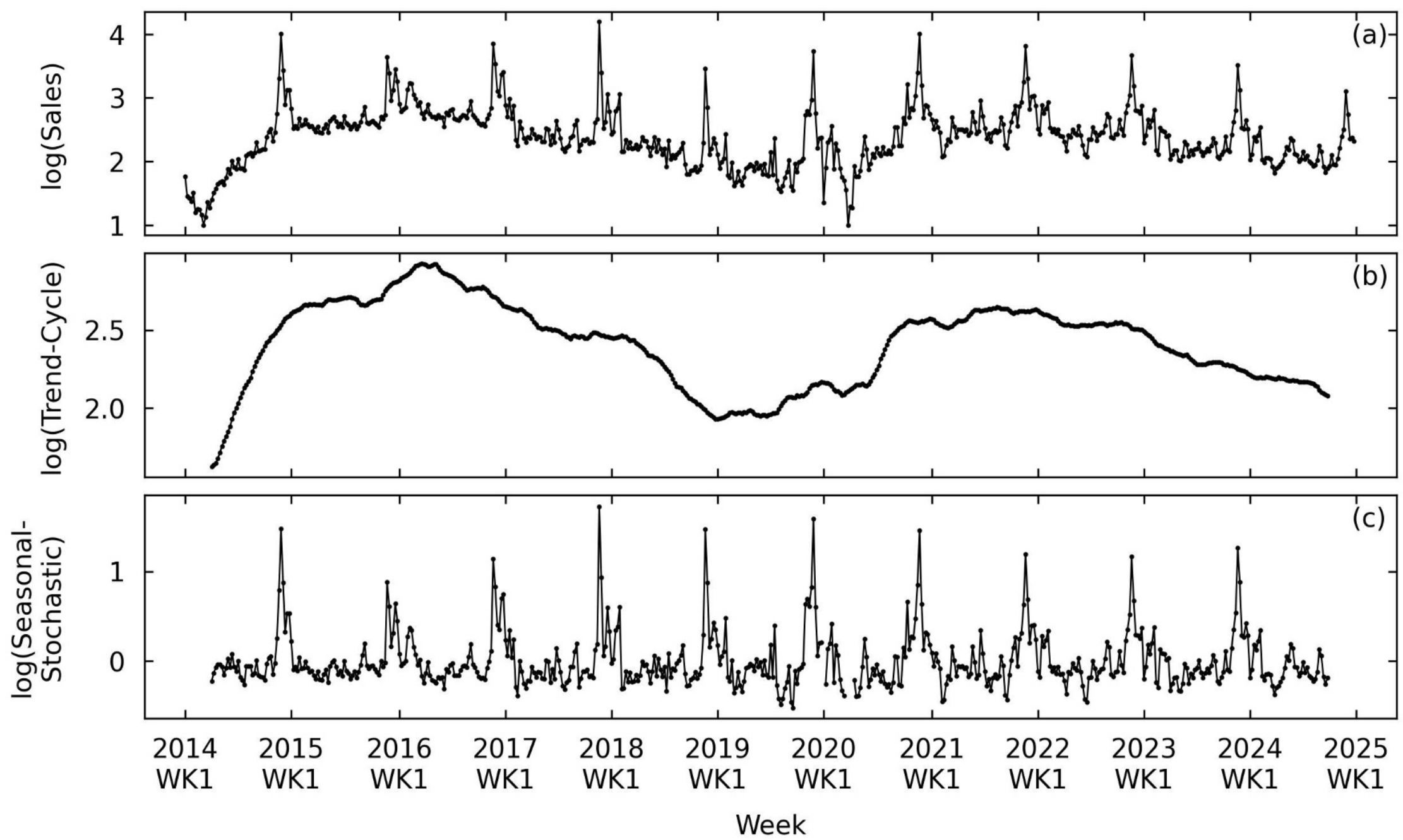

Fig S5-1. Time series decomposition using parameter $\ell = 13$: (a) time series of weekly TV (log-)sales from 2014 to 2024; (b) logarithm of its trend-cycle component; and (c) logarithm of its seasonal-stochastic component.

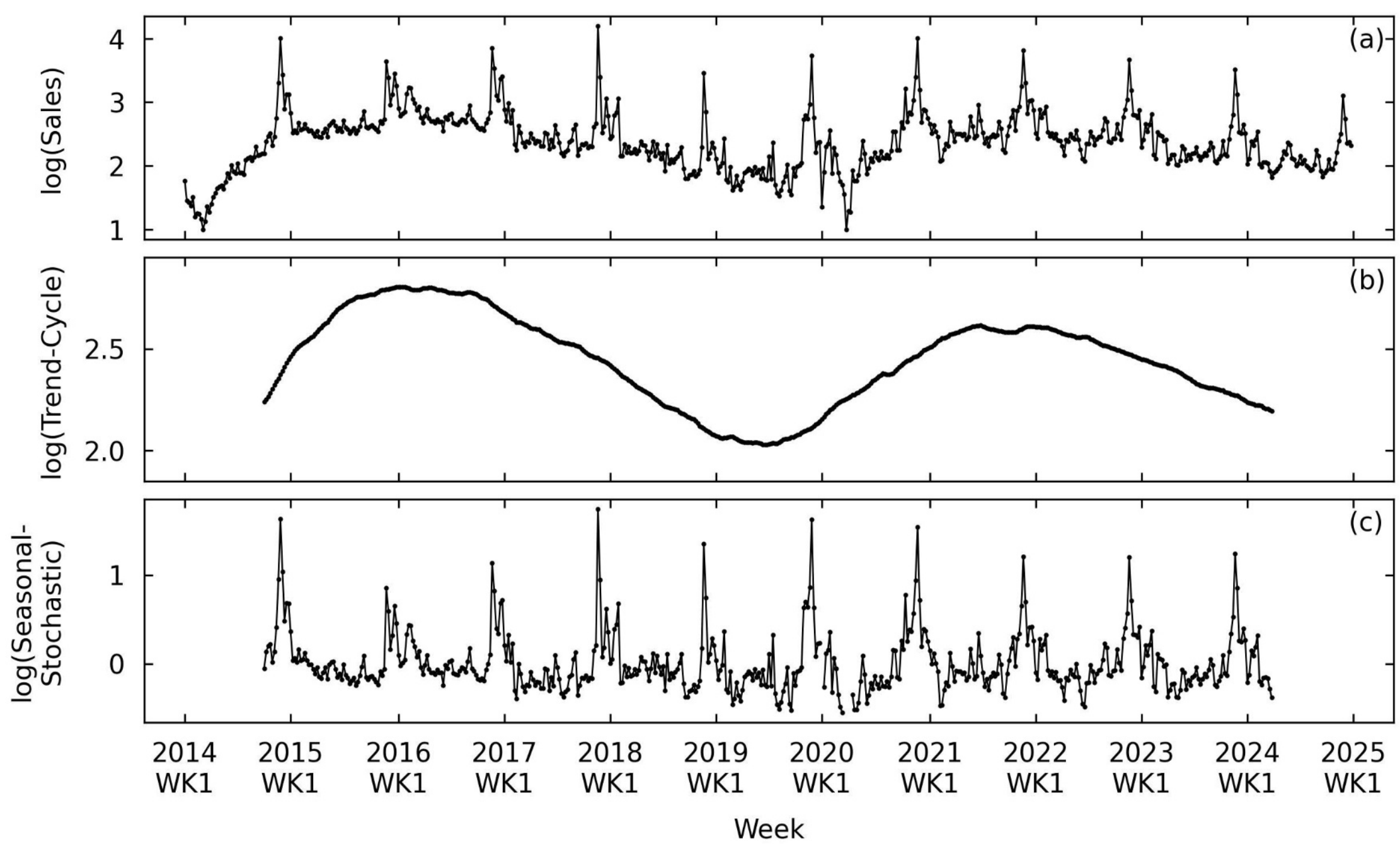

Fig S5-2. Time series decomposition using parameter $\ell = 39$: (a) time series of weekly TV (log-)sales from 2014 to 2024; (b) logarithm of its trend-cycle component; and (c) logarithm of its seasonal-stochastic component.

Table S5-1. Averages of accuracy metrics MAE, RMSE, MAPE, WMAPE and SMAPE computed in windows of 53 weeks from 2021 to 2024 for TV sales forecasts using horizon $\tau = 1$ week for different time series decomposition parameter ℓ and autoregressive correction parameter λ (standard deviations in parenthesis).

Parameter ℓ	Parameter λ	MAE	RMSE	MAPE	WMAPE	SMAPE
$\ell = 6$	$\lambda = \ell$	1.247 (0.359)	2.104 (0.670)	0.095 (0.018)	0.100 (0.018)	0.093 (0.018)
	$\lambda = 26$	1.271 (0.346)	2.108 (0.615)	0.097 (0.017)	0.103 (0.016)	0.095 (0.017)
$\ell = 13$	$\lambda = \ell$	1.349 (0.360)	2.071 (0.557)	0.105 (0.019)	0.109 (0.018)	0.103 (0.019)
	$\lambda = 26$	1.275 (0.338)	2.054 (0.587)	0.098 (0.016)	0.103 (0.016)	0.096 (0.016)
$\ell = 19$	$\lambda = \ell$	1.235 (0.328)	2.035 (0.612)	0.095 (0.015)	0.100 (0.015)	0.093 (0.015)
	$\lambda = 26$	1.214 (0.350)	2.051 (0.653)	0.093 (0.017)	0.098 (0.017)	0.090 (0.017)
$\ell = 26$	$\lambda = \ell$	1.194 (0.350)	1.991 (0.614)	0.091 (0.016)	0.096 (0.017)	0.089 (0.016)
	$\lambda = 26$	1.194 (0.350)	1.991 (0.614)	0.091 (0.016)	0.096 (0.017)	0.089 (0.016)
$\ell = 32$	$\lambda = \ell$	1.187 (0.312)	1.928 (0.561)	0.093 (0.014)	0.096 (0.014)	0.091 (0.014)
	$\lambda = 26$	1.179 (0.319)	1.930 (0.590)	0.092 (0.014)	0.095 (0.015)	0.090 (0.014)
$\ell = 39$	$\lambda = \ell$	1.230 (0.322)	2.005 (0.609)	0.096 (0.014)	0.099 (0.014)	0.094 (0.015)
	$\lambda = 26$	1.227 (0.303)	2.031 (0.613)	0.096 (0.013)	0.099 (0.012)	0.093 (0.013)
