# Supplementary material for: Weekly sales forecasting using time series decomposition with irregular seasonality: A case study of Sony TV sales in the United States
Source: PLoS One. 2026 Nov 3;21(11):e0344222. doi: 10.1371/journal.pone.0344222 (PMC13730381; doi:10.1371/journal.pone.0344222)
Supplement: Supplementary file 6 [file pone.0344222.s006.pdf]

Weekly sales forecasting using time series decomposition with irregular seasonality: A case study of Sony TV sales in the United States

Arthur Matsuo Yamashita Rios de Sousa, Daisuke Ando, Junichi Ozaki, Yohei Shida, Qianyun Wu, Yixuan Y. Zheng, Zhihua Zhong, Rie Maskawa, Jiwei J. Jiang, Kay Nagayama, Hideki Takayasu, Misako Takayasu

S6 Appendix: Autoregressive correction for s_t forecasting

For the forecasting of the seasonal-stochastic component s_t, we consider a first-order autoregressive model for the z-scores of s_t to account for possible autocorrelation. The choice of order 1 for the autoregression can be empirically justified by a partial autocorrelation analysis. For that, we compute the partial autocorrelation coefficients for lags 1, 2 and 3 in windows of $\lambda = 26$ weeks. Statistical significance is assessed using a permutation test with 10,000 random permutations of the observations within each window. For each lag, the p-value is estimated as the proportion of permuted partial autocorrelation coefficients whose absolute deviation from the mean of the permutation distribution is at least as large as that of the observed coefficient. Fig S6-1 presents the results of the partial autocorrelation analysis. The partial autocorrelation at lag 1 is consistently positive and statistically significant for most of the considered period, while the partial autocorrelations at lags 2 and 3 are generally smaller, fluctuating around zero, and only occasionally significant. Most of the serial dependence is thus explained by the first lag, supporting the choice of the order 1 for the autoregressive model.

Fig S6-2 shows the estimated autoregressive parameter $\hat{\phi}$ computed in windows of $\lambda = 26$ weeks for each forecasting origin. We observe that its values are always positive, mostly ranging between 0.25 and 0.75, indicating persistent positive serial dependence. Only on few occasions $\hat{\phi}$ is close to zero, evidencing that the effect of autocorrelation is important, and it never approaches 1, which would violate the stationarity assumption.

As explained in the main text, parameter λ controls how far in the past we look to estimate the serial correlation in the data. We suggested the heuristic rule $\lambda = \ell$, which for our case study corresponds to $\lambda = 26$. In order to evaluate the effect of this parameter, we perform a sensitivity analysis across a range of values of λ. Table S6-1 reports the forecasting results for horizon $\tau = 1$ week for different values of the parameter. The case $\lambda = 0$ refers to the absence of the autoregressive correction and the resulting higher error metrics indicate that incorporating serial correlation information is indeed beneficial for forecasting. The suggested value $\lambda = 26$ yields the best overall results, although nearby values produce similar forecasting accuracy, showing that the method is fairly robust to moderate misspecification of λ, provided that the autocorrelation structure is adequately estimated.

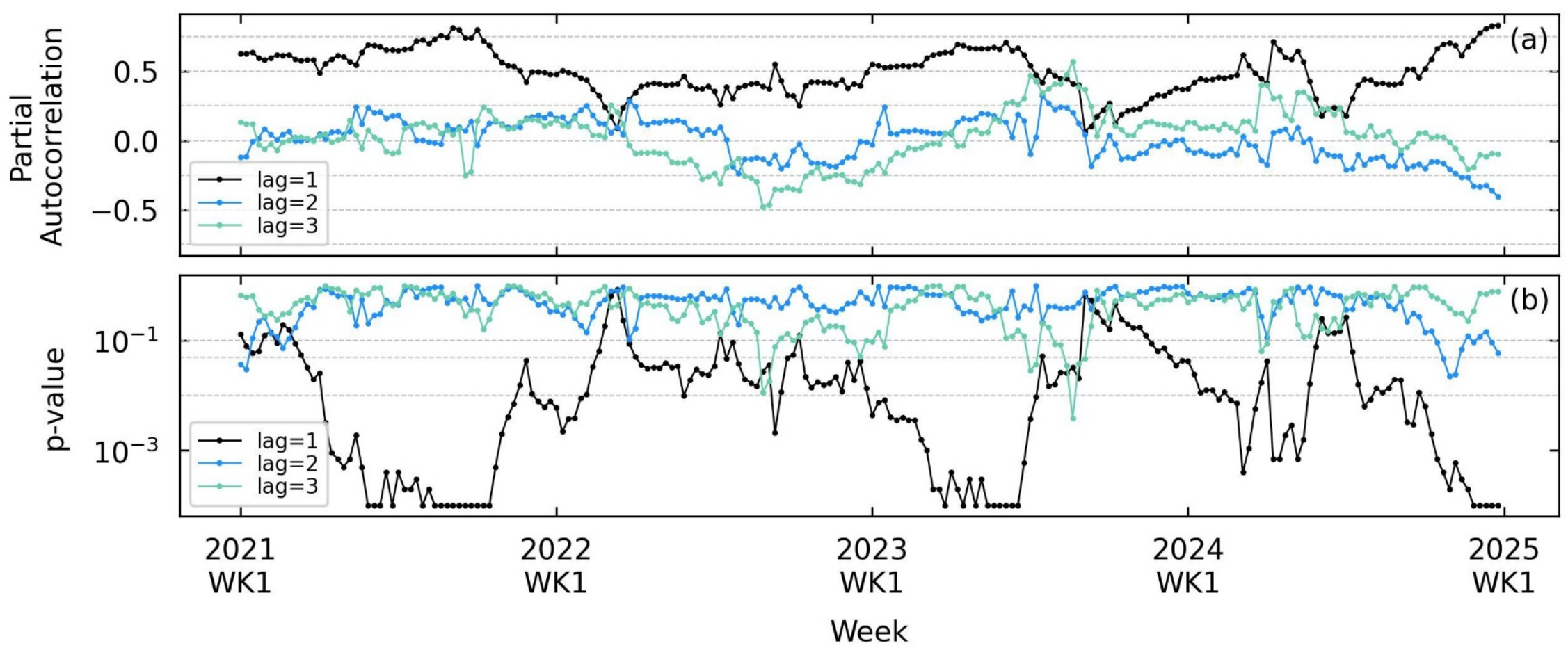

Fig S6-1. Partial autocorrelation analysis of the z-scores of the seasonal–stochastic component in windows of $\lambda = 26$ weeks from 2021 to 2024: (a) partial autocorrelation coefficients and (b) the corresponding permutation-test p-values for lags 1 (black symbols), 2 (light blue symbols) and 3 (light green symbols). Horizontal dashed lines in (b) indicate significance levels of 0.10, 0.05 and 0.01.

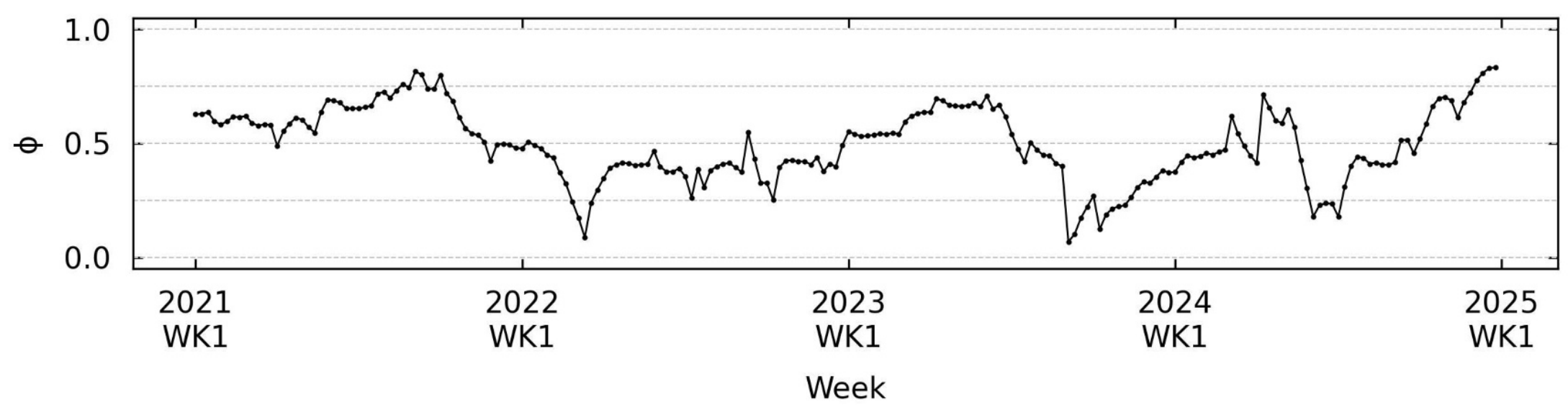

Fig S6-2. Estimates of autoregressive parameter ϕ in windows of $\lambda = 26$ weeks from 2021 to 2024.

Table S6-1. Averages of accuracy metrics MAE, RMSE, MAPE, WMAPE and SMAPE computed in windows of 53 weeks from 2021 to 2024 for TV sales forecasts using horizon $\tau = 1$ week for different autoregressive correction parameter λ (standard deviations in parenthesis).

Parameter λ	MAE	RMSE	MAPE	WMAPE	SMAPE
$\lambda = 0$	1.365 (0.331)	2.009 (0.508)	0.110 (0.015)	0.111 (0.015)	0.106 (0.014)
$\lambda = 13$	1.252 (0.314)	1.981 (0.544)	0.097 (0.012)	0.101 (0.013)	0.094 (0.012)
$\lambda = 26$	1.194 (0.350)	1.991 (0.614)	0.091 (0.016)	0.096 (0.017)	0.089 (0.016)
$\lambda = 39$	1.211 (0.339)	2.030 (0.637)	0.093 (0.015)	0.098 (0.016)	0.090 (0.015)
$\lambda = 53$	1.225 (0.342)	2.017 (0.619)	0.094 (0.015)	0.099 (0.016)	0.092 (0.015)
$\lambda = 65$	1.226 (0.351)	2.042 (0.650)	0.094 (0.016)	0.099 (0.016)	0.092 (0.017)
