# Supplementary material for: Weekly sales forecasting using time series decomposition with irregular seasonality: A case study of Sony TV sales in the United States
Source: PLoS One. 2026 Nov 3;21(11):e0344222. doi: 10.1371/journal.pone.0344222 (PMC13730381; doi:10.1371/journal.pone.0344222)
Supplement: Supplementary file 7 [file pone.0344222.s007.pdf]

Weekly sales forecasting using time series decomposition with irregular seasonality: A case study of Sony TV sales in the United States

Arthur Matsuo Yamashita Rios de Sousa, Daisuke Ando, Junichi Ozaki, Yohei Shida, Qianyun Wu, Yixuan Y. Zheng, Zhihua Zhong, Rie Maskawa, Jiwei J. Jiang, Kay Nagayama, Hideki Takayasu, Misako Takayasu

S7 Appendix: Forecasting without analogous weeks

Grouping weeks with similar sales behavior is essential for improving forecast accuracy. To demonstrate this, we perform the forecasting without using analogous weeks, i.e., grouping weeks solely based on their numbers regardless of holidays or events. We still take outlier weeks into account. Results are shown in Fig S7-1 and should be compared with Fig 7 in the main text. We observe that accuracy is worse across all periods and metrics. The effect is particularly visible for Black Friday weeks, which occur in either week 47 or week 48; incorrectly grouping them increases errors and widens prediction intervals. Table S7-1 summarizes the average of the accuracy metrics for the forecasting with and without analogous weeks.

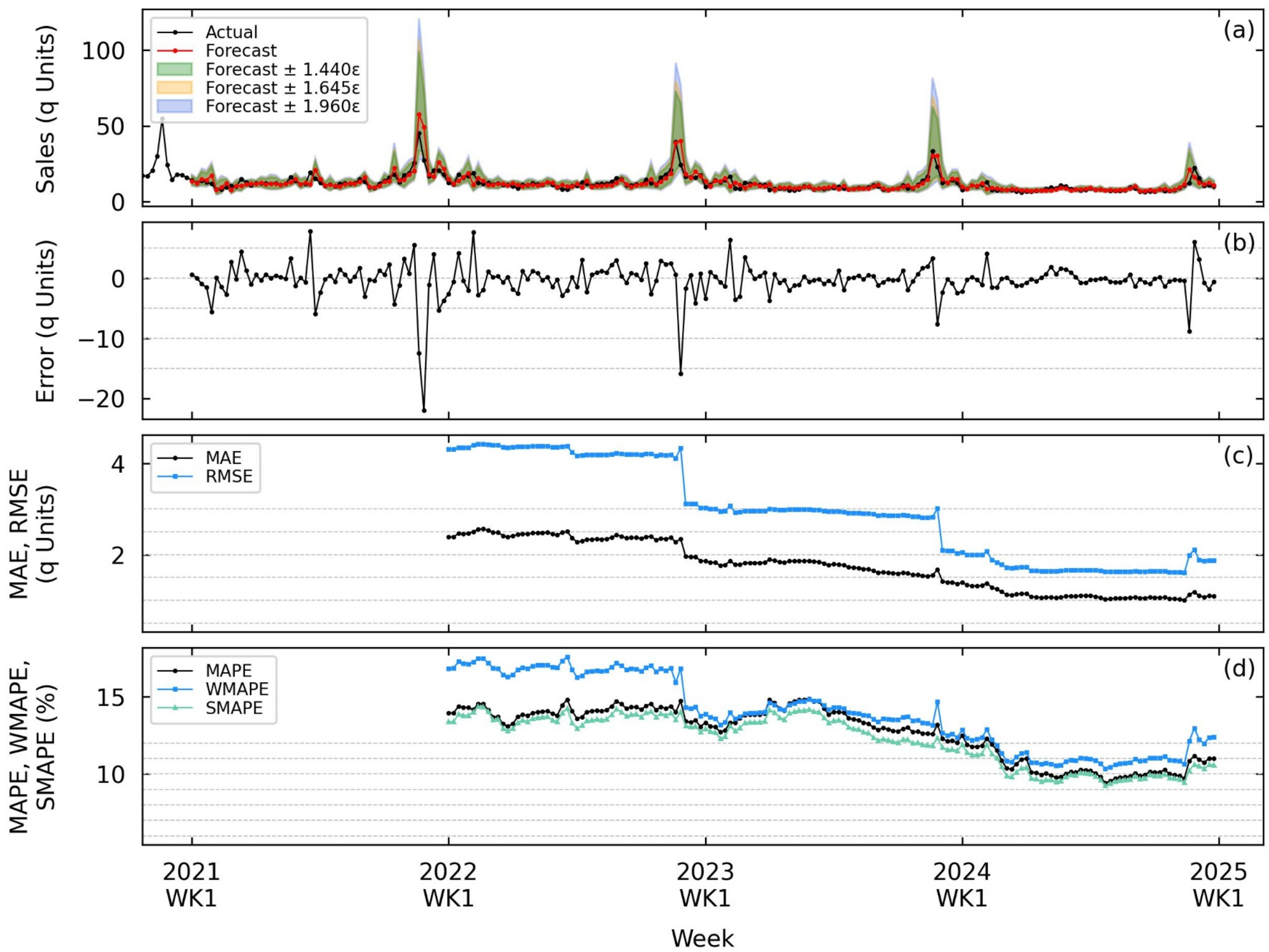

Fig S7-1. Forecasting results without using analogous weeks: (a) Actual time series of weekly TV sales from 2021 to 2024 (black symbols) and forecasts using horizon of 1 week (red symbols) with prediction intervals of 1.440 (green shade), 1.645 (orange shade) and 1.960 (blue shade) standard deviations; (b) forecast errors; (c) MAE (black circles) and RMSE (light blue squares) computed in windows of 53 weeks; and (d) MAPE (black circles), WMAPE (light blue squares) and SMAPE (light green triangles) computed in windows of 53 weeks.

Table S7-1. Averages of accuracy metrics MAE, RMSE, MAPE, WMAPE and SMAPE computed in windows of 53 weeks from 2021 to 2024 for TV sales forecasts using horizon $\tau = 1$ week with and without analogous weeks (standard deviations in parenthesis).

Analogous Weeks	MAE	RMSE	MAPE	WMAPE	SMAPE
With	1.194<br>(0.350)	1.991<br>(0.614)	0.091<br>(0.016)	0.096<br>(0.017)	0.089<br>(0.016)
Without	1.735<br>(0.532)	2.931<br>(1.038)	0.127<br>(0.017)	0.139<br>(0.023)	0.122<br>(0.016)
