# Supplementary material for: Weekly sales forecasting using time series decomposition with irregular seasonality: A case study of Sony TV sales in the United States
Source: PLoS One. 2026 Nov 3;21(11):e0344222. doi: 10.1371/journal.pone.0344222 (PMC13730381; doi:10.1371/journal.pone.0344222)
Supplement: Supplementary file 8 [file pone.0344222.s008.pdf]

Weekly sales forecasting using time series decomposition with irregular seasonality: A case study of Sony TV sales in the United States

Arthur Matsuo Yamashita Rios de Sousa, Daisuke Ando, Junichi Ozaki, Yohei Shida, Qianyun Wu, Yixuan Y. Zheng, Zhihua Zhong, Rie Maskawa, Jiwei J. Jiang, Kay Nagayama, Hideki Takayasu, Misako Takayasu

S8 Appendix: (Log-)Normality assumption

The prediction intervals are constructed under the assumption that the forecast of the log-sales $\tilde{x}_{n+\tau}$ is normally distributed (with distribution parameters that may vary across forecast weeks) and, consequently, the sales forecast $\tilde{X}_{n+\tau}$ follows a log-normal distribution. To assess the adequacy of this assumption, we show in Fig S8-1 the normal Q-Q plots (quantile–quantile plots) of the standardized forecast errors for TV log-sales forecasts from 2021 to 2024 using horizons $\tau = 1$, 4, 13 and 26 weeks. The plots compare the empirical quantiles of the standardized forecast errors with the corresponding theoretical quantiles of a standard normal distribution. If the normal approximation is appropriate, the points in the plots should lie close to the reference identity line (central red dashed line) and remain within the corresponding approximate 95% confidence bands (outer red dashed lines). Systematic departures from the reference line indicate deviations from the assumed normal distribution, providing a visual assessment of the calibration of the prediction intervals.

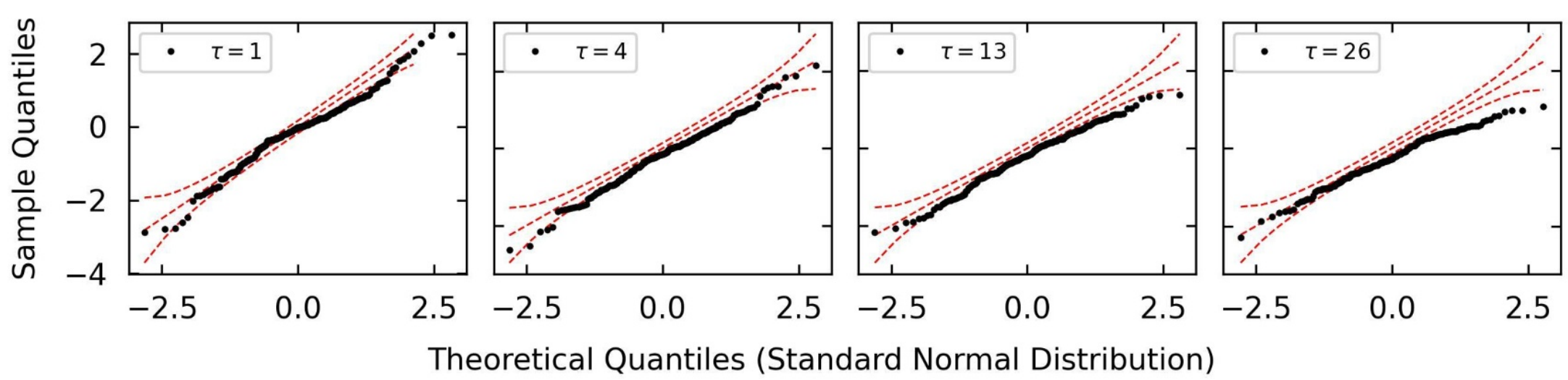

Fig S8-1. Normal Q-Q plots of the standardized forecast errors for TV log-sales forecasts from 2021 to 2024 using horizons of 1, 4, 13 and 26 weeks. Black symbols represent the sample quantiles. Red dashed lines correspond to the theoretical quantiles of the standard normal distribution and the corresponding 95% confidence bands.

The Q-Q plot for horizon $\tau = 1$ week provides support for the normal approximation, with the standardized forecast errors lying mostly within the confidence bands. For $\tau = 4$, the points are systematically shifted downward, indicating a small negative bias. Nevertheless, they remain approximately linear and within the confidence bands, suggesting only a minor departure from the assumed normal distribution. Similar behavior is observed for $\tau = 13$, with the points tending to lie close to the lower confidence band. The largest departure from normality is observed for the 26-week horizon, where the upper tail falls below the theoretical normal quantiles, indicating that the standardized forecast errors exhibit smaller dispersion than expected under the normal assumption. Consequently, the estimated forecast uncertainty for horizon $\tau = 26$ is overestimated, producing prediction intervals that are wider than necessary and therefore conservative (overcoverage), as reported in Table 3 of the main text.

One possible source of the prediction interval miscalibration is the assumption made in Eq (46) of the main text that the forecasts of the trend-cycle and seasonal-stochastic components are

independent. We evaluate the practical importance of this assumption by examining the correlation between the corresponding forecast errors. Since the estimated components are updated as additional observations become available, there is no unique ground-truth value for either component: to compute the forecast errors, we use as reference the first estimates of the components that can be obtained once sufficient future observations become available. The variance of the total forecast error, expressed in terms of the trend-cycle forecast error $E^{(c)}$ and the seasonal-stochastic forecast error $E^{(s)}$, is in general given by:

$$Var(E^{(c)} + E^{(s)}) = Var(E^{(c)}) + Var(E^{(s)}) + 2Cov(E^{(c)}, E^{(s)}). \tag{S8-1}$$

Eq (46) of the main text assumes that the covariance term is negligible and the variance of the total forecast error can be approximated by the sum of the component variances. Table S8-1 reports the empirical correlation between the trend-cycle and seasonal-stochastic forecast errors and the ratio between the covariance term and the sum of the component variances in Eq S8-1. The ratio is particularly informative, showing that the covariance term is less than 6% of the combined variances for all forecasting horizons. These results indicate that the covariance has only a negligible effect on the estimated forecast uncertainty and that the independence approximation is unlikely to be the primary source of the observed interval miscalibration. Note that this analysis is not a direct test of independence, but rather an assessment of the practical impact of neglecting the covariance term when estimating forecast uncertainty.

Table S8-1. Correlation between the (log-)trend-cycle and (log-)seasonal-stochastic components forecast errors for TV sales forecasts from 2021 to 2024 using horizons of 1, 4, 13, and 26 weeks. The ratio measures the magnitude of the covariance between components relative to the sum of their variances.

Horizon (Weeks)	Correlation	Ratio
$\tau = 1$	-0.068	0.038
$\tau = 4$	0.109	0.056
$\tau = 13$	0.074	0.048
$\tau = 26$	-0.025	0.021

Continuing the investigation of possible sources of the prediction interval miscalibration, Table S8-2 summarizes the averages of forecast standard deviations for sales $\varepsilon^{(x)}_{n+\tau}$, trend-cycle component $\varepsilon^{(c)}_{n+\tau}$ and seasonal-stochastic component $\varepsilon^{(s)}_{n+\tau}$ using horizons $\tau = 1$, 4, 13 and 26 weeks. Observing that ${\varepsilon^{(x)}_{n+\tau}}^2 = {\varepsilon^{(c)}_{n+\tau}}^2 + {\varepsilon^{(s)}_{n+\tau}}^2$ from Eq (46) of the main text, we also report in the last two columns the average contribution of each component to the total forecast variance. Since $\varepsilon^{(x)}_{n+\tau}$ controls the width of the prediction intervals, decomposing it into its components helps identify which one dominates the forecast uncertainty. While the seasonal-stochastic component contributes most to the uncertainty for short horizons (67.8% for $\tau = 1$), the contribution of the trend-cycle component steadily increases with the forecasting horizon, exceeding the seasonal-stochastic component for $\tau = 26$. Accordingly, the forecast standard deviation of the trend-cycle component increases from 0.069 for $\tau = 1$ to 0.145 for $\tau = 26$, while the forecast standard deviation of the seasonal-stochastic component changes only modestly from 0.122 to 0.140, revealing that the growth in forecast uncertainty at longer horizons is primarily driven by the trend-cycle component. This finding is consistent with the overcoverage observed for long forecasting horizons and suggests that it is mainly associated with an overestimation of the trend-cycle forecast standard deviation. Further improvements in trend-cycle forecasting are thus expected to contribute to the calibration of long-horizon prediction intervals.

Table S8-2. Averages of forecast standard deviations of the (log-)sales $\varepsilon^{(x)}_{n+\tau}$, (log-)trend-cycle component $\varepsilon^{(c)}_{n+\tau}$ and (log-)seasonal-stochastic component $\varepsilon^{(s)}_{n+\tau}$ computed from 2021 to 2024 for TV sales forecasts using horizons of 1, 4, 13 and 26 weeks. Last two columns show the average contributions of the trend-cycle and seasonal-stochastic components to the total forecast variance (standard deviations in parenthesis).

Horizon (Weeks)	$\langle \varepsilon^{(x)}_{n+\tau} \rangle$	$\langle \varepsilon^{(c)}_{n+\tau} \rangle$	$\langle \varepsilon^{(s)}_{n+\tau} \rangle$	$\langle {\varepsilon^{(c)}_{n+\tau}}^2 / {\varepsilon^{(x)}_{n+\tau}}^2 \rangle$	$\langle {\varepsilon^{(s)}_{n+\tau}}^2 / {\varepsilon^{(x)}_{n+\tau}}^2 \rangle$
$\tau = 1$	0.143<br>(0.056)	0.069<br>(0.004)	0.122<br>(0.063)	0.322<br>(0.184)	0.678<br>(0.184)
$\tau = 4$	0.160<br>(0.068)	0.078<br>(0.004)	0.136<br>(0.076)	0.338<br>(0.195)	0.662<br>(0.195)
$\tau = 13$	0.174<br>(0.057)	0.107<br>(0.005)	0.133<br>(0.068)	0.455<br>(0.198)	0.545<br>(0.198)
$\tau = 26$	0.207<br>(0.063)	0.145<br>(0.007)	0.140<br>(0.079)	0.570<br>(0.196)	0.430<br>(0.196)
