# Supplementary material for: Weekly sales forecasting using time series decomposition with irregular seasonality: A case study of Sony TV sales in the United States
Source: PLoS One. 2026 Nov 3;21(11):e0344222. doi: 10.1371/journal.pone.0344222 (PMC13730381; doi:10.1371/journal.pone.0344222)
Supplement: Supplementary file 9 [file pone.0344222.s009.pdf]

Review of the paper:

Weekly sales forecasting using time series decomposition with irregular seasonality: A case study of Sony TV sales in the United States

The authors argue that they present in the paper "…a simple and interpretable forecasting method for weekly sales…". They argue that the main challenge "…is to properly address irregular seasonality arising from the way a year is divided into weeks and from floating holidays and events…". The authors propose the concept of "analogous weeks", which is described in the paper as "… a general framework for grouping weeks that are similar according to some criteria, which encodes seasonality and already accounts for calendar effects.". They also argue that, incorporating the proposed concept into the time series decomposition and forecasting of the case study, they "…achieved yearly average percentage errors of less than 10% for the 1-week-ahead forecast and less than 15% for forecast horizons of up to 26 weeks, accuracy levels not reached by other comparable established seasonal models, namely Prophet and SARIMAX.".

A) Originality of Research

It is my criteria that the level of originality of research is satisfactory, but I have these comments:

The *analogous weeks* concept is the paper's primary intellectual contribution. The authors correctly situate it relative to prior work on historical analogues (used mainly for special events), arguing that their framework generalizes the idea to *all* weeks systematically. This is a defensible and useful extension. The matrix formulation of the decomposition (Eqs. 16–24) is a noteworthy theoretical contribution: it shows that the decomposition reduces to linear transformations involving the matrix W (specifically Wx for the trend-cycle component and (I−W)x for the seasonal-stochastic component) where W depends only on the calendar and outlier structure, not on the data themselves. This has useful implications for multi-series applications.

However, several concerns limit the novelty claim:

1) The decomposition procedure, according with the paper (section "Proposed decomposition") is related to X-11-ARIMA and STL methods. The main contribution of the paper is the replacement of a fixed-period seasonal averaging with analogous-week averaging, which may be an incremental value added to previous methodologies.
2) The paper references Arora and Taylor (references [55, 56] of the paper). The authors should clearly establish what is added to these methods in the proposed analogous weeks methodology.
3) There is no contribution to the forecasting model for the trend-cycle component described on equation 25 as it is a standard OLS regression on the lagged trend level and slope.
4) The authors do not discuss if this concept has been suggested (even by a different name, or in a partial or modified way) in proprietary industry practice, where such calendar-based grouping is common in retail forecasting.

B) Results Not Published Elsewhere:

I found no evidence of prior publication. The methodology appears original to this submission. The dataset is proprietary of Sony Group Corporation.

C) Technical Standards and Methodological Rigor:

The technical contribution of the paper is the analogous weeks concept.

The matrix formulation of the decomposition (in equations 16-24) is a valuable theoretical contribution because it shows that the decompositions reduce to a single linear transformation that depends only on the calendar and outlier structure and not on the data themselves. This formulation may have useful implications for multi-series applications. However, I have several concerns about their methodology.

Major Comments:

1) The comparisons between the proposed method and benchmarks rely entirely on point estimates of accuracy metrics (MAE, RSME, MAPE, WMAPE, and SMAPE Table 1, Fig. 10). Point estimates alone do not tell whether observed differences between models are statistically meaningful or merely due to sampling variability. No statistical tests of forecast accuracy differences are conducted. Even when the differences in some cases seems visually clear, in some cases may not be statistically significant, particularly for SARIMAX at $\tau = 1$ (e.g., proposed method shows 1.194 vs. SARIMAX 1.216, with a standard deviations of 0.350 and 0.396, respectively, the difference is 0.022 which imply that this difference of is approximately 1/16 of one standard deviation). Authors should consider the use of Diebold-Mariano test or the modified Harvey-Leybourne-Newbold version for the pairwise comparisons. In this issue, maybe the authors may wish to consider to acknowledge that for $\tau = 1$ is essentially a tie with SARIMAX, and arguing that the contribution of the paper (at least with for the case presented in the paper) is centered in medium-to-longer horizon stability.
2) The entire empirical evaluation rests on one time series (total weekly Sony TV unit sales in the United States) spanning 574 weeks (2014–2024). Every accuracy number in Table 1, every curve in Figures 7–10, and every coverage figure in Table 2 derives exclusively from this single series. The conclusion that the proposed method "outperformed standard models" is drawn solely from this evidence base. The forecasting literature has established that no method dominates universally across datasets (see, e.g., Makridakis, S. et. al, 1982, 2000, 2020, and Wolpert, D. H., & Macready, W. G. (1997). It is impossible to determine from a single series whether the proposed method's apparent superiority is robust or specific to this dataset's characteristics. The authors should either,

evaluate on additional publicly available retail or weekly sales datasets, or substantially moderate the generalizability of their conclusions.

3) The paper states that two key parameters ($\lambda = \ell = 26$) are selected heuristically, justified by the approximate annual period. No sensitivity analysis is conducted. Sensitivity analysis is required.
4) The paper established that equation (46) assumes independence (between the trend-cycle and seasonal-stochastic forecast errors) to compute the combined standard deviation of the log-sales forecast. This assumption is not tested empirically and may not hold, potentially leading to miscalibrated prediction intervals.
5) The paper models the z-scores z_t (defined in Equation (34)) with an AR(1) process: $z_t = \rho z_{t-1} + \eta \varphi_t$ where ρ is the autoregressive coefficient (estimated as $\hat{\rho}$ in Equation (38)), η is the innovation standard deviation (estimated as $\hat{\eta}$ in Equation (39)), and φ_t is i.i.d. N(0,1). The choice of order one for this autoregressive model is not empirically motivated. No ACF or PACF analysis of the z-scores is provided to justify this order for the Sony TV dataset. Furthermore, the estimated value of $\hat{\rho}$ and its behavior across rolling origins are never reported. This matters for two reasons:

   a) If $\hat{\rho}$ is near zero across most rolling windows, the AR(1) correction contributes negligibly to forecast accuracy, and the added complexity is unnecessary. The simpler forecast $\tilde{s}_{n+\tau} = \bar{s}_{n+\tau}$ would suffice.
   b) If $\hat{\rho}$ is near 1, the stationarity of the z-score process is questionable, which would invalidate the AR(1) model assumptions underlying Equations (35) – (44).

   Additionally, there is a notational "conflict" in the paper that should be resolved: the symbol ρ is used for two entirely different quantities in close proximity:

   a) $\rho^{(\tau)}$ in Equation (25): the standard deviation of the trend-cycle forecast error (a scaling parameter).
   b) ρ in Equations (35) and (38): the AR(1) autoregressive coefficient for the z-score process.

   These are conceptually unrelated quantities, and their shared symbol is a source of genuine confusion, particularly because both appear in the forecasting section. The recommended resolution is to replace $\rho^{(\tau)}$ in Equation (25) with $\sigma^{(\tau)}$, which is consistent with the standard deviation notation used elsewhere in the paper ($\sigma^{(c)}$, $\sigma^{(s)}$, $\sigma^{(x)}$, $\sigma^{(z)}$ in Eqs. (31), (42), (44), (46)).

6) The authors of the paper removed 4 weeks, March to April 2020, because they considered them as outliers. The authors argue that the COVID-19 stimulus payments are treated as irregular events. However, the COVID-19 period involved not only demand shocks but also structural supply disruptions that likely persisted. The boundary between the outlier weeks and the subsequent recovery period is not clearly defined and its impact on the

estimated decomposition is not assessed. Therefore, a robustness check excluding 2020 entirely (or a several months period after April 2020) may strengthen the analysis.

7) The prediction intervals rely on log-normality of sales, however, no diagnostic (e.g., Shapiro-Wilk or Jarque-Bera test on residuals) is provided. The authors acknowledge in the conclusions that large deviations from the (log-)normality assumption could be problematic but offer no evidence on where their dataset stands.
8) For the proposed method, ℓ and λ are fixed at 26 throughout all rolling origins (not optimized per origin). For Prophet Fine-Tuned, a grid search is performed at each origin. For SARIMA/SARIMAX, auto_arima selects parameters at each origin. This creates a comparison asymmetry: the proposed method uses fixed hyper-parameters while competitors use adaptive ones. The paper should either optimize ℓ and λ adaptively (consistent with competitors) or fix the competitors' parameters analogously and discuss what this choice implies for the comparison.

Minor comments:

1) Page 5, item (c): The judgment that "Best Buy Early Black Friday Deals" is similar to "Amazon Prime Day week" is non-obvious and deserves more explicit justification beyond the term "temporal proximity."
2) Page 9, Equation (25): As it was mentioned previously (major comment 5 of this review), the use of the symbol ρ is used for two entirely different quantities:

   a) $\rho^{(\tau)}$ in Equation (25): the standard deviation of the trend-cycle forecast error, a scaling parameter for the error term $\xi_{t+\tau}$.
   b) ρ in Equation (35) and $\hat{\rho}$ in Equation (38): the AR(1) autoregressive coefficient for the z-score process z_t.

   Both quantities appear in the forecasting section in close proximity, and their shared symbol constitutes a genuine notational conflict that will confuse readers. The recommended resolution is to replace $\rho^{(\tau)}$ in Equation (25) with $\sigma^{(\tau)}$, consistent with the standard deviation symbols used throughout the uncertainty quantification section: $\sigma^{(c)}_{n+\tau}$ in Equation (31), $\sigma^{(z)}_{n+\tau}$ in Equation (42), $\sigma^{(s)}_{n+\tau}$ in Equation (44), and $\sigma^{(x)}_{n+\tau}$ in Equation (46).

3) The trend-cycle forecasting regression in Equation (25): $c_{t+\tau} = b^{(\tau)}_0 + b^{(\tau)}_1 \cdot c_{t-\ell} + b^{(\tau)}_2 \cdot \beta_{t-\ell} + \rho^{(\tau)} \cdot \xi_{t+\tau}$ uses $c_{t-\ell}$ and $\beta_{t-\ell}$ (the trend-cycle level and local linear slope both lagged $\ell = 26$ weeks from the current time point t) as regressors. This occurs because the decomposition defines c_t and β_t only within the set $U_\ell = [1 + \ell, n - \ell]$, meaning these quantities are unavailable for the most recent ℓ weeks of the training set due to the symmetric window requirement of the local linear regression in Equation (8). The consequence is that at forecasting origin n, the most recent available trend-cycle information is 26 weeks old.

This is a non-trivial design choice with two implications the paper does not discuss. First, for the 1-week-ahead forecast ($\tau = 1$), the model predicts c_{n+1} using c_{n-26} and β_{n-26}, discarding 26 weeks of the most recent trend information. Second, this creates an asymmetry with the seasonal-stochastic component forecast, which does incorporate the most recent observation x_n through the current z-score $\tilde{z}_n$ in Equation (41). The paper cites Dagum & Bianconcini (reference [59]) on asymmetric window estimation techniques that could in principle make c_n and β_n available, but this technique is not applied. The authors should either justify explicitly why using $c_{n-\ell}$ and $\beta_{n-\ell}$ is preferable to asymmetric boundary estimation or test the alternative and compare accuracy.

4) Table 1 highlights the minimum value for each accuracy metric and each forecast horizon in bold. This visual emphasis implies that the highlighted method is meaningfully superior to its nearest competitor. However, as argued in the major comment 1, no formal significance test is conducted. For $\tau = 1$, the differences between the proposed method and SARIMAX are so small relative to the standard deviations that the highlighted minima are statistically meaningless without supporting test results. The authors should either add significance test results to Table 1 or remove the bold highlighting and replace it with a more cautious presentation.
5) The overcoverage at $\tau = 26$ (empirical coverage 0.923 vs. theoretical 0.85) is notable but receives only one sentence of discussion. Is this due to the trend uncertainty dominating at longer horizons? A decomposition of the interval width into trend and seasonal contributions would be informative.

D) Adequacy and Appropriateness of Conclusions:

The central claim that the proposed method outperforms Prophet and SARIMAX/SARIMA in this case study is supported by the data as presented, particularly at longer horizons. However, several conclusions are stated too broadly:

1) The abstract states accuracy "not reached by other comparable established seasonal models." This is too strong given the single-series evaluation and absence of significance testing.
2) The conclusion that the method "outperformed standard models" should be qualified to reflect the specific dataset, parameter settings, and lack of statistical testing.
3) The discussion of prediction intervals (Table 2) is largely descriptive. The overcoverage at $\tau = 26$ is noted but not explained or corrected for. This suggests the uncertainty model may be misspecified at longer horizons.
4) The conclusion section appropriately identifies limitations (intermittency, structural breaks, log-normality) but these should appear more prominently, as they are non-trivial for many practical demand forecasting applications.

E) Clarity and Writing Quality:

The paper is well-organized and written in fluent English. The logical flow from data description → decomposition → forecasting → empirical evaluation is clear. The use of a diagram (Fig. 3) to illustrate the iterative decomposition algorithm is particularly effective.

F) Ethics, Research Integrity, and Data Availability:

With regards of these topics, I only have these concerns:

1) The data are proprietary (Sony Group Corporation) and actual sales figures have been masked by an undisclosed scaling factor. This is acknowledged by the authors, but it raises concerns: (a) the results cannot be independently replicated; (b) the magnitude of actual errors is unknown to readers; and (c) it is unclear whether all figures and tables were reviewed by Sony Group Corporation for competitive sensitivity, which could introduce selective reporting risk.
2) The paper should include a data availability statement explicitly noting that the data cannot be shared and explaining what can be made available (e.g., code).
3) The analogous week rules (Section "Analogous weeks") contain several subjective decisions (e.g., assignment of Amazon Prime Day 2014, similarity of COVID stimulus to Memorial Day, treatment of holiday overlaps). These are documented in the paper, which is commendable, but no sensitivity analysis tests whether alternative judgments would materially affect results.
4) No code repository is referenced. Given the methodological contribution, sharing the implementation (even on an alternative or synthetic dataset) would substantially increase reproducibility and research integrity.

G) **Adherence to Reporting Guidelines and Community Standards:**

I have no major comments in regard of these topics.

RECOMMENDATION AND REQUIRED REVISIONS:

It is my opinion that the paper makes a genuine, if incremental, methodological contribution and addresses a real practical problem with care, however, it cannot be accepted in its current form. I strongly suggest the author perform, at least, the following revisions:

Required revisions:

1) Conduct formal statistical tests of forecast accuracy differences (Diebold-Mariano or equivalent) for all pairwise comparisons at all horizons. Revise claims of superiority accordingly.
2) Evaluate the method on at least one additional publicly available weekly sales or demand dataset to support generalizability claims. If this is not possible (e.g., due to data

constraints), the scope of conclusions must be radically narrowed to explicitly characterize this as a single-case-study with limited external validity.

3) Provide a sensitivity analysis for parameters ℓ and λ, demonstrating the robustness of results to alternative choices.
4) Provide diagnostics for the log-normality assumption (residual Q-Q plots, formal tests) and for the AR(1) order selection (ACF/PACF of z-scores, estimated $\hat{\rho}$ over rolling origins).
5) Make code publicly available, even if applied to a synthetic or publicly available dataset, to ensure reproducibility.

Recommended revisions:

1) Discuss and analyze the asymmetry in parameter optimization between the proposed method and benchmarks, and assess its impact on the comparison.
2) Add a robustness check for the COVID-19 period (e.g., results excluding 2020) and provide further analysis of the Black Friday overprediction pattern.
3) Unify and clarify notation throughout (especially the forecast horizon symbol and the intercept/slope symbols in the decomposition equations).

References:

[1] Makridakis, S., Andersen, A., Carbone, R., Fildes, R., Hibon, M., Lewandowski, R., Newton, J., Parzen, E., & Winkler, R. (1982). The accuracy of extrapolation (time series) methods: Results of a forecasting competition. Journal of Forecasting, 1(2), 111–153. https://doi.org/10.1002/for.3980010202

[2] Makridakis, S., & Hibon, M. (2000). The M3-competition: Results, conclusions and implications. International Journal of Forecasting, 16(4), 451–476. https://doi.org/10.1016/S0169-2070(00)00057-1

[3] Makridakis, S., Spiliotis, E., & Assimakopoulos, V. (2020). The M4 competition: 100,000 time series and 61 forecasting methods. International Journal of Forecasting, 36(1), 54–74. https://doi.org/10.1016/j.ijforecast.2019.04.014

[4] Wolpert, D. H., & Macready, W. G. (1997). No free lunch theorems for optimization. IEEE Transactions on Evolutionary Computation, 1(1), 67–82. https://doi.org/10.1109/4235.585893
