# Supplementary material for: Weekly sales forecasting using time series decomposition with irregular seasonality: A case study of Sony TV sales in the United States
Source: PLoS One. 2026 Nov 3;21(11):e0344222. doi: 10.1371/journal.pone.0344222 (PMC13730381; doi:10.1371/journal.pone.0344222)
Supplement: Supplementary file 10 [file pone.0344222.s010.pdf]

Manuscript title: Weekly sales forecasting using time series decomposition with irregular seasonality: A case study of Sony TV sales in the United States

Manuscript ID: PONE-D-26-08207

Recommendation: Major revision

This manuscript proposes a time series decomposition method for forecasting weekly sales, using Sony TV sales in the United States (2014–2024) as a case study. The central contribution is the concept of "analogous weeks". This is a rule-based framework for grouping weeks that share similar calendar characteristics (holidays, events, or position within the year), which addresses irregular seasonality arising from variable year lengths and floating holidays. The method decomposes the log-transformed sales into trend-cycle and seasonal-stochastic components, forecasts each independently, and recombines them. The authors find that the proposed approach achieves yearly average percentage errors below 10% for 1-week-ahead forecasts and below 15% for horizons up to 26 weeks, outperforming Prophet and SARIMAX benchmarks across multiple accuracy metrics.

The manuscript addresses a practically relevant problem with a conceptually clear and interpretable solution. The "analogous weeks" framework is a useful contribution that could benefit practitioners seeking transparent forecasting methods. However, the study has notable limitations regarding generalizability (a single product/company/country), reliance on subjective judgment to define analogous weeks, and insufficient statistical testing of performance differences.

Major comments

1. Limited generalizability and external validity

The study uses a single product category (TVs), single company (Sony), and single geographic market (United States). While the authors acknowledge this as a "case study," the claims about the method's superiority lack external validation. It cannot be assessed whether the approach generalizes to other products, markets, or industries with different demand patterns. The authors should explicitly discuss generalizability limitations in the Discussion section, ideally test the method on at least one additional publicly available dataset, or provide guidance on how practitioners should adapt the analogous weeks rules for different contexts.

2. Subjective judgment in "analogous weeks" definition

The rules for defining analogous weeks (Section: Analogous weeks) involve substantial forecaster judgment, particularly for irregular events (e.g., "COVID-19 Economic Impact Payments similar to Memorial Day week"). While the authors state such judgments should be "clearly documented," this subjectivity undermines reproducibility and introduces potential bias. Impact: Different practitioners applying the method may obtain different forecasts depending on their judgment. The authors should provide sensitivity analyses showing how forecast accuracy changes under alternative analogous week assignments. Consider formalizing decision criteria or providing a decision tree to guide practitioners.

3. Parameter selection lacks rigorous justification

The parameters ℓ = 26 and λ = 26 are selected heuristically based on the "yearly periodicity" argument (lines 266–268, 339–341). No cross-validation, grid search, or sensitivity analysis is provided to justify these choices. It is recommended that the authors conduct sensitivity analyses across a range of ℓ and λ values (e.g., 13, 26, 39, 52) and report performance variation. Alternatively, implement a time-series cross-validation approach to select parameters within the training window.

4. Statistical significance of performance differences not assessed

Table 1 reports means and standard deviations of accuracy metrics, but no statistical tests (e.g., Diebold-Mariano test, paired t-tests, or non-parametric alternatives) are conducted to determine whether differences between the proposed method and benchmarks are statistically significant. The authors should apply appropriate statistical tests for comparing forecast accuracy and report p-values or confidence intervals for performance differences.

5. Handling of COVID-19 period raises concerns

The treatment of COVID-19 weeks as outliers (removed from training) and COVID-19 Economic Impact Payments as irregular events (analogous to Memorial Day) involves strong assumptions. The pandemic fundamentally altered consumer behavior in ways that may not match pre-pandemic patterns. Impact: The model's performance during and after the pandemic may be artificially inflated by excluding difficult-to-predict periods. The authors should provide separate accuracy metrics for pre-COVID (2014–2019) and post-COVID (2021–2024) periods. Discuss how the method would perform in real-time during unprecedented events when appropriate analogies are unavailable.

6. Convergence condition verification missing

The authors note (lines 289–290) that convergence requires the spectral radius of $W_A W_L$ to be strictly less than 1, but state that "cases for which the convergence condition does not hold remain to be studied." It is unclear whether convergence was verified for the actual data analyzed. The authors should explicitly report the spectral radius computed for the analyzed data and confirm convergence.

7. Visual presentation and figure quality require substantial improvement

Several figures in the manuscript present formatting and accessibility issues that hinder reader comprehension. First, some figures do not appear to be properly formatted to fit within standard page dimensions, which may result in cropping or reduced legibility in the published version. Second, Figure 2 relies on color coding to distinguish different event types (e.g., regular events, irregular events, weeks removed from training), yet the figure itself lacks a self-contained legend explaining the meaning of each color. This information is provided only in the main text (lines 114–120), requiring readers to cross-reference between the figure and prose. Third, Figure 4 is not comprehensible in its current form (the visual elements, axes, or data representation are unclear, preventing adequate evaluation of the content it is meant to convey).

Minor comments

1. Reference 2 (lines 512–514) appears distorted: “forecasting: Research R, practice. Fildes, Robert…” should be corrected to proper citation format.

2. Line 47: “X-13ARIMA-SEATS” should include a brief explanation or citation upon first mention for readers unfamiliar with seasonal adjustment software.

3. Lines 99–100: The phrase “masked by dividing them by a factor q” would benefit from stating whether q is constant across time or how this masking affects variance properties.

4. Table 1: Consider adding a row showing the percentage improvement of the proposed method over the best competitor for easier interpretation.

5. The prediction interval analysis (Table 2) would benefit from reporting calibration metrics (e.g., PIT histograms) beyond coverage and width.

6. Line 393: “1.440, 1.645 and 1.960 standard deviations” should clarify these correspond to approximately 85%, 90%, and 95% for two-tailed normal intervals.
