# Supplementary material for: Weekly sales forecasting using time series decomposition with irregular seasonality: A case study of Sony TV sales in the United States
Source: PLoS One. 2026 Nov 3;21(11):e0344222. doi: 10.1371/journal.pone.0344222 (PMC13730381; doi:10.1371/journal.pone.0344222)
Supplement: Supplementary file 11 [file pone.0344222.s012.pdf]

Review of:

Weekly sales forecasting using time series decomposition with irregular seasonality: A case study of Sony TV sales in the United States

Assessment

The paper proposes a simple and interpretable forecasting method that involves the concept of "analogous weeeks", and show that the forecasting performance of the method is as good or better than Prophet and SARIMAX. I am not convinced the degree of originality is sufficiently high to merit publication, and the details underpinning the method is incomplete and unconvincing. For example, in the proposed decomposition (pp. 6-9) it is stated that least squares is used to derive the estimates, but the authors are not explicit about the exact definition of the error. This is particularly important in the current context, since estimation is in logs whereas the series to forecast is not. As is well known, an adjustment is needed for correct forecasting in these cases, and this adjustment depends on the distribution of the error, see for example Duan (1983). My recommendation to the Editor(s), therefore, is that the paper is rejected.

Other

1. Page 7, line 259. Make explicit that $l \in \{1, \ldots, n\}$? Also, mention already here that l is a user-specified tuning parameter?

2. Page 7, between equations (6) and (7). Introduce a new set, say, O that contains the outlier weeks? Next, re-define V to $V = U - O$?

3. Page 7. The Iverson bracket notation is not common in time series, so it should be defined. Also, the authors should ask themselves: Is it really needed? Additionally, it appears the authors use $|A|$ to denote the number of elements in a set A. This should also be made explicit.

References

Duan, N. (1983). Smearing Estimate: A Nonparametric Retransformation Method. *Journal of the Americal Statistical Association 78*, pp. 605–610.
