# Supplementary material for: Weekly sales forecasting using time series decomposition with irregular seasonality: A case study of Sony TV sales in the United States
Source: PLoS One. 2026 Nov 3;21(11):e0344222. doi: 10.1371/journal.pone.0344222 (PMC13730381; doi:10.1371/journal.pone.0344222)
Supplement: Supplementary file 12 [file pone.0344222.s013.pdf]

Response to Reviewers

Manuscript ID: PONE-S-26-10788
Title: Weekly sales forecasting using time series decomposition with irregular seasonality: A case study of Sony TV sales in the United States

The authors thank the reviewers for carefully reading our manuscript and for their questions, comments and suggestions that contributed to its improvement. Please find below our point-by-point responses (in blue).

Response to Reviewer #1

The authors argue that they present in the paper "... a simple and interpretable forecasting method for weekly sales...". They argue that the main challenge "... is to properly address irregular seasonality arising from the way a year is divided into weeks and from floating holidays and events...". The authors propose the concept of "analogous weeks", which is described in the paper as "... a general framework for grouping weeks that are similar according to some criteria, which encodes seasonality and already accounts for calendar effects.". They also argue that, incorporating the proposed concept into the time series decomposition and forecasting of the case study, they "... achieved yearly average percentage errors of less than 10% for the 1-week-ahead forecast and less than 15% for forecast horizons of up to 26 weeks, accuracy levels not reached by other comparable established seasonal models, namely Prophet and SARIMAX.".

Our answer: The reviewer provides a fair summary of our work. We thank the reviewer for the honest and detailed assessment of our manuscript.

A) Originality of Research
It is my criteria that the level of originality of research is satisfactory, but I have these comments:
The analogous weeks concept is the paper's primary intellectual contribution. The authors correctly situate it relative to prior work on historical analogues (used mainly for special events), arguing that their framework generalizes the idea to all weeks systematically. This is a defensible and useful extension. The matrix formulation of the decomposition (Eqs. 16-24) is a noteworthy theoretical contribution: it shows that the decomposition reduces to linear transformations involving the matrix W (specifically Wx for the trend-cycle component and (I-W)x for the seasonal-stochastic component) where W depends only on the calendar and outlier structure, not on the data themselves. This has useful implications for multi-series applications.
However, several concerns limit the novelty claim:

Our answer: We appreciate the reviewer's positive evaluation of the originality of our research and for highlighting its main contributions. We address the concerns about novelty below.

1) The decomposition procedure, according with the paper (section "Proposed decomposition") is related to X-11-ARIMA and STL methods. The main contribution of the paper is the replacement of a fixed-period seasonal averaging with analogous-week averaging, which may be an incremental value added to previous methodologies.
Our answer: The reviewer is correct in this observation. Our decomposition procedure follows the general strategy of "iteratively determine both components simultaneously", as found in X-11-ARIMA and STL methods. The contribution of our work does not lie in proposing a fundamentally new decomposition paradigm, but rather in showing how to systematically incorporate the concept of 'analogous weeks' into time series decomposition to address irregular seasonality. Additionally, we demonstrate that our method reduces to linear transformations through the matrix formulation, which "highlights the simplicity of the proposed decomposition".

2) The paper references Arora and Taylor (references [55, 56] of the paper). The authors should clearly establish what is added to these methods in the proposed analogous weeks methodology.
Our answer: We agree that the relationship with references Arora and Taylor was not sufficiently clear. We modified the text to make explicit the need for adaptations and extensions in our weekly sales case: "Building on the general idea of rule-based approaches [56,57], with adaptations and extensions to the weekly sales setting considered here and its specific characteristics, such as years with either 52 or 53 weeks and the presence of irregular events as described in the Data Description section, we construct the following rules to define the set of analogous weeks of a given week" (lines 187-191).

3) There is no contribution to the forecasting model for the trend-cycle component described on equation 25 as it is a standard OLS regression on the lagged trend level and slope.
Our answer: The reviewer is correct. Using a standard OLS regression model was intentional, as one of the objectives of this work is to develop a simple and interpretable forecasting method. Note, however, that the regressors are constructed using the proposed decomposition, which is where our contribution lies. We added to the text: "While linear regression is a standard model, the regressors are constructed from the proposed decomposition" (lines 342-343).

4) The authors do not discuss if this concept has been suggested (even by a different name, or in a partial or modified way) in proprietary industry practice, where such calendar-based grouping is common in retail forecasting.
Our answer: To the best of our knowledge, we are not aware of the concept of analogous weeks in the form proposed in this study being used in industry practice. We acknowledge, however, that proprietary forecasting methods are often not publicly documented. Therefore, we cannot exclude the possibility that related approaches may exist under different names or in modified forms. We added to the manuscript: "Although related concepts may exist in industry practice, the proposed framework provides a systematic and publicly documented approach to define and apply analogous weeks" (lines 85-87).

B) Results Not Published Elsewhere:
I found no evidence of prior publication. The methodology appears original to this submission. The dataset is proprietary of Sony Group Corporation.

Our answer: No comments from our side.

C) Technical Standards and Methodological Rigor:
The technical contribution of the paper is the analogous weeks concept.
The matrix formulation of the decomposition (in equations 16-24) is a valuable theoretical contribution because it shows that the decompositions reduce to a single linear transformation that depends only on the calendar and outlier structure and not on the data themselves. This formulation may have useful implications for multi-series applications. However, I have several concerns about their methodology.
Our answer: We address the concerns about the methodology below.

Major Comments:
1) The comparisons between the proposed method and benchmarks rely entirely on point estimates of accuracy metrics (MAE, RSME, MAPE, WMAPE, and SMAPE Table 1, Fig. 10). Point estimates alone do not tell whether observed differences between models are statistically meaningful or merely due to sampling variability. No statistical tests of forecast accuracy differences are conducted. Even when the differences in some cases seems visually clear, in some cases may not be statistically significant, particularly for SARIMAX at $\tau = 1$ *(e.g., proposed method shows 1.194 vs. SARIMAX 1.216, with a standard deviations of 0.350 and 0.396, respectively, the difference is 0.022 which imply that this difference of is approximately 1/16 of one standard deviation). Authors should consider the use of Diebold-Mariano test or the modified Harvey-Leybourne-Newbold version for the pairwise comparisons. In this issue, maybe the authors may wish to consider to acknowledge that for* $\tau = 1$ *is essentially a tie with SARIMAX, and arguing that the contribution of the paper (at least with for the case presented in the paper) is centered in medium-to-longer horizon stability.*
Our answer: We thank the reviewer for this important comment regarding the statistical significance of the observed differences in accuracy between methods. We agree that this analysis is necessary and, as suggested by the reviewer, have applied the Diebold–Mariano test with the Harvey–Leybourne–Newbold small-sample correction. Results are displayed in Table 2 and discussed in the corresponding section. The statistical analysis supports the main findings from the point accuracy metrics and also provides a more nuanced interpretation of some comparisons. The proposed method produces significantly more accurate forecasts than Prophet with default parameters and SARIMA, while no significant difference from SARIMAX is found for short forecast horizons (which is explicit in the text: "with the proposed method and SARIMAX performing the best for the considered forecast horizon" and "ARIMAX is similar in accuracy to our method for $\tau = 1$"). One additional observation that arose from this analysis is that Prophet with fine-tuned parameters produces forecasts with statistically similar accuracy to the proposed method for intermediate horizons, which we interpret as "suggesting that the effectiveness of the fine-tuning strategy may depend on the forecast horizon". Our revised summary of the findings, incorporating the above observations, reads as: "Overall, these results show that the competitive performance of the proposed method in this case study is not only reflected in lower error metrics but is also statistically supported in most comparisons. The benchmarks Prophet and SARIMAX produce forecasts with similar accuracy to the proposed method under specific conditions: when Prophet parameters are effectively fine-tuned and when SARIMAX is applied to short forecast horizons" (lines 515-520).

2) The entire empirical evaluation rests on one time series (total weekly Sony TV unit sales in the United States) spanning 574 weeks (2014–2024). Every accuracy number in Table 1, every curve in Figures 7–10, and every coverage figure in Table 2 derives exclusively from this single series. The conclusion that the proposed method "outperformed standard models" is drawn solely from this evidence base. The forecasting literature has established that no method dominates universally across datasets (see, e.g., Makridakis, S. et. al, 1982, 2000, 2020, and Wolpert, D. H., & Macready, W. G. (1997). It is impossible to determine from a single series whether the proposed method's apparent superiority is robust or specific to this dataset's characteristics. The authors should either, evaluate on additional publicly available retail or weekly sales datasets, or substantially moderate the generalizability of their conclusions.

Our answer: We are in agreement with the reviewer that the use of a single time series limits the empirical support for any given method. However, our main objective is not to claim universal applicability of the proposed method, but rather to introduce the concept of 'analogous weeks' and to demonstrate, through a detailed case study, how it can be systematically incorporated into time series decomposition and forecasting to address irregular seasonality – while at the same time contributing to the discussion on simplicity and interpretability in forecasting methods.

Although evaluation on additional datasets would provide broader empirical evidence, single-dataset case studies are also reported in the literature when focusing on a specific dataset and/or introducing a generalizable concept that is not tied to a particular method. In our references, we have three examples: reference [34] analyzed SKU (Stock Keeping Unit) data from a 'manufacturing company specializing in household products' to 'show that judgmental adjustments can enhance baseline forecasts during promotions'; reference [46] analyzed Australian domestic tourism demand data to evaluate which hierarchical forecasting method is the most suitable for the specific Australian tourism market; and reference [57] analyzed France electricity demand data in a case study on load forecasting for France by introducing a rule-based methodology to improve forecasts for special days. Our work follows a similar case-study approach: we focus on the previously unpublished dataset of weekly Sony TV sales and introduce the 'analogous weeks' framework to deal with irregular seasonality.

In our conclusion, we acknowledge that the method may require adaptation when the characteristics of the data differ substantially from those of our case study and included the explanation: "However, no forecasting method consistently dominates across all datasets and the most appropriate approach depends on the characteristics of the data and forecasting problem [17,18]" (lines 561-563). We also moderated our wording: "Despite its simplicity, it achieved comparable performance to or outperformed standard models in the task of forecasting weekly TV sales in our case study" (lines 548-550).

3) The paper states that two key parameters ($\lambda = \ell = 26$) are selected heuristically, justified by the approximate annual period. No sensitivity analysis is conducted. Sensitivity analysis is required.

Our answer: We thank the reviewer for this recommendation. To address this concern, we have added "S5 Appendix: Parameter ℓ of time series decomposition" and "S6 Appendix: Autoregressive correction for s_t forecasting", which present sensitivity analyses for the parameters ℓ and λ. The results indicate the robustness of the proposed method across a reasonable range of the parameters and support $\ell = \lambda = 26$ as practical default values.

4) The paper established that equation (46) assumes independence (between the trend-cycle and seasonal-stochastic forecast errors) to compute the combined standard deviation of the log-sales forecast. This assumption is not tested empirically and may not hold, potentially leading to miscalibrated prediction intervals.

Our answer: Following the reviewer's comment, in the added "S7 Appendix: (Log-)Normality assumption" we investigate the practical impact of the independence assumption used in Eq. (46) by analyzing the empirical correlation between the trend-cycle and seasonal-stochastic forecast errors. We show that the covariance term arising from dependence between components represents less than 6% of the combined component variances for all forecasting horizons, indicating that "the independence approximation is unlikely to be the primary source of the observed interval miscalibration".

5) The paper models the z-scores z_t (defined in Equation (34)) with an AR(1) process: z_t = ρ z_{t-1} + η ϕ_t where ρ is the autoregressive coefficient (estimated as $\hat{\rho}$ in Equation (38)), η is the innovation standard deviation (estimated as $\hat{\eta}$ in Equation (39)), and ϕ_t is i.i.d. N(0,1). The choice of order one for this autoregressive model is not empirically motivated. No ACF or PACF analysis of the z-scores is provided to justify this order for the Sony TV dataset. Furthermore, the estimated value of $\hat{\rho}$ and its behavior across rolling origins are never reported. This matters for two reasons:

a) If $\hat{\rho}$ is near zero across most rolling windows, the AR(1) correction contributes negligibly to forecast accuracy, and the added complexity is unnecessary. The simpler forecast $\tilde{s}$_{n+τ} = $\bar{s}$_{n+τ} would suffice.

b) If $\hat{\rho}$ is near 1, the stationarity of the z-score process is questionable, which would invalidate the AR(1) model assumptions underlying Equations (35)-(44).

Our answer: Following the reviewer's recommendation, we have added "S6 Appendix: Autoregressive correction for s_t forecasting", which contains a partial autocorrelation analysis that empirically justifies the choice of order 1 for the autoregressive model. We also report the estimated autoregressive parameter $\hat{\phi}$ and its behavior across rolling origins. The estimates are always positive, rarely close to zero and never approach one, indicating that the autoregressive correction provides a meaningful contribution to forecasting while remaining consistent with the stationarity assumption. Additionally, we show that omitting the autoregressive correction degrades forecasting performance, demonstrating that the effect of serial correlation is not negligible.

Additionally, there is a notational "conflict" in the paper that should be resolved: the symbol ρ is used for two entirely different quantities in close proximity:

a) $\rho\hat{(\tau)}$ in Equation (25): the standard deviation of the trend-cycle forecast error (a scaling parameter).

b) ρ in Equations (35) and (38): the AR(1) autoregressive coefficient for the z-score process.

These are conceptually unrelated quantities, and their shared symbol is a source of genuine confusion, particularly because both appear in the forecasting section. The recommended resolution is to replace $\rho\hat{(\tau)}$ in Equation (25) with $\sigma\hat{(\tau)}$, which is consistent with the standard deviation notation used elsewhere in the paper ($\sigma\hat{(c)}$, $\sigma\hat{(s)}$, $\sigma\hat{(x)}$, $\sigma\hat{(z)}$ in Eqs. (31), (42), (44), (46)).

Our answer: The symbol corresponding to the AR(1) coefficient in Equation (35) is not ρ (rho), but φ, a variant of letter ϕ (phi). We agree that this notation may be confusing

to the reader, so we have replaced it to the standard letter ϕ.

6) The authors of the paper removed 4 weeks, March to April 2020, because they considered them as outliers. The authors argue that the COVID-19 stimulus payments are treated as irregular events. However, the COVID-19 period involved not only demand shocks but also structural supply disruptions that likely persisted. The boundary between the outlier weeks and the subsequent recovery period is not clearly defined and its impact on the estimated decomposition is not assessed. Therefore, a robustness check excluding 2020 entirely (or a several months period after April 2020) may strengthen the analysis.

Our answer: As noted in the manuscript, the outlier weeks were not selected by us but were directly informed by Sony based on known disruptions in production and supply. Nevertheless, we agree that the effects of the COVID-19 pandemic may have extended beyond the initial shock. Following the reviewer's suggestion, in the added "S2 Appendix: COVID-19 outliers", we conducted a robustness check by progressively extending the assumed COVID outlier period beyond the 4 weeks originally informed by Sony. The results show that forecast accuracy is robust to moderate extensions of the COVID outlier period. Overall, the additional analysis suggests that extending the outlier period beyond the initial disruption does not improve forecasting performance, providing empirical support for the adequacy of the original COVID outlier period informed by Sony.

7) The prediction intervals rely on log-normality of sales, however, no diagnostic (e.g., Shapiro-Wilk or Jarque-Bera test on residuals) is provided. The author acknowledge in the conclusions that large deviations from the (log-)normality assumption could be problematic but offer no evidence on where their dataset stands.

Our answer: We thank the reviewer for this important comment. We have added "S7 Appendix: (Log-)Normality assumption" to provide evidence regarding the (log-)normality assumption used to construct the prediction intervals. In particular, we employ normal Q-Q plots of the residual sto visually assess the adequacy of the normal approximation. The plots indicate that the normal approximation is reasonable for horizons $\tau = 1$, 4, and 13, while moderate deviations are observed for $\tau = 26$. These deviations are consistent with the overcoverage observed for longer-horizon forecasts. Additional analysis in the same Appendix shows that this behavior appears to be mainly associated with an overestimation of the trend-cycle component uncertainty.

8) For the proposed method, ℓ and λ are fixed at 26 throughout all rolling origins (not optimized per origin). For Prophet Fine-Tuned, a grid search is performed at each origin. For SARIMA/SARIMAX, auto_arima selects parameters at each origin. This creates a comparison asymmetry: the proposed method uses fixed hyper-parameters while competitors use adaptive ones. The paper should either optimize ℓ and λ adaptively (consistent with competitors) or fix the competitors' parameters analogously and discuss what this choice implies for the comparison.

Our answer: The reviewer raises an interesting point and, indeed, our decision to separate Prophet into two categories was made in order to have a transparent comparison between the methods: in Prophet Default, hyper-parameters are fixed as in our proposed method, and in Prophet Fine-Tuned, we allow parameter optimization using past data. For SARIMA/SARIMAX, we also fix the method specification choices: "we set the seasonal frequency as 52 weeks, treat irregular events and outliers as missing values and use the presence of holidays and regular events as exogenous variables in SARIMAX (other

than that, we use the default parameters)". What is adaptive in this case is the selection of the SARIMA/SARIMAX model orders, which follows the standard practice for these models, where order identification is considered part of the modeling process. We have modified the text to explain this aspect: "method 'auto_arima' for automatic selection of the model orders (autoregressive, differencing and moving-average orders, as well as seasonal orders when applicable)" (lines 449-451) and "Since there are no default values for the SARIMA and SARIMAX model orders, the orders are selected and the models are refitted at each forecast origin" (line 464-465).
We also remind the reviewer about the sensitivity analyses in the added "S5 Appendix: Parameter ℓ of time series decomposition" and "S6 Appendix: Autoregressive correction for s_t forecasting", showing that variations in ℓ and λ (within reasonable ranges) have small impact on forecasting accuracy.

Minor comments:
1) Page 5, item (c): The judgment that "Best Buy Early Black Friday Deals" is similar to "Amazon Prime Day week" is non-obvious and deserves more explicit justification beyond the term "temporal proximity."
Our answer: We have added the following paragraph to further explain the suggested rules: "Rules for ordinary weeks, holidays and regular events are straightforward due to their yearly recurrence. For irregular events, the assignment of analogous weeks requires forecaster judgment. Similarity in event characteristics should be preferred when available (e.g., Amazon Prime Day 2 similar to Amazon Prime Day). When such information is not available or is ambiguous, temporal proximity should be used as a secondary criterion (e.g., COVID-19 Economic Impact Payments assumed similar to Memorial Day week because it is the nearest regular holiday with increase in sales and Best Buy Early Black Friday Deals assumed similar to Amazon Prime Day because it occurs in the week after Amazon Prime Day 2)" (line 205-213).

2) Page 9, Equation (25): As it was mentioned previously (major comment 5 of this review), the use of the symbol ρ is used for two entirely different quantities:
a) $\rho\hat{(}\tau)$ in Equation (25): the standard deviation of the trend-cycle forecast error, a scaling parameter for the error term ξ_{t+τ}.
b) ρ in Equation (35) and $\hat{\rho}$ in Equation (38): the AR(1) autoregressive coefficient for the z-score process z_t.
Both quantities appear in the forecasting section in close proximity, and their shared symbol constitutes a genuine notational conflict that will confuse readers. The recommended resolution is to replace $\rho\hat{(}\tau)$ in Equation (25) with $\sigma\hat{(}\tau)$, consistent with the standard deviation symbols used throughout the uncertainty quantification section: $\sigma\hat{(}c)\{n + \tau\}$ in Equation (31), $\sigma\hat{(}z\{n + \tau\}$ in Equation (42), $\sigma\hat{(}s)\{n + \tau\}$ in Equation (44), and $\sigma\hat{(}x)\{n + \tau\}$ in Equation (46).
Our answer: Answered above (comment 5 in Major Comments).

3) The trend-cycle forecasting regression in Equation (25): c_{$t + \tau$} = $b\hat{(}\tau)$_0 + $b\hat{(}\tau)$_1 · c_{$t - \ell$} + $b\hat{(}\tau)$_2 · β_{$t - \ell$} + $\rho\hat{(}\tau)$ · ξ_{$t + \tau$} uses c_{$t - \ell$} and β_{$t - \ell$} (the trend-cycle level and local linear slope both lagged ℓ = 26 weeks from the current time point t) as regressors. This occurs because the decomposition defines c_t and β_t only within the set U_ℓ= [1 + ℓ, n - ℓ], meaning these quantities are unavailable for the most recent ℓ weeks of the training set due to the symmetric window requirement of the local

linear regression in Equation (8). The consequence is that at forecasting origin n, the most recent available trend-cycle information is 26 weeks old.
This is a non-trivial design choice with two implications the paper does not discuss. First, for the 1-week-ahead forecast (τ = 1), the model predicts c_{n + 1} using c_{n - 26} and β_{n - 26}, discarding 26 weeks of the most recent trend information. Second, this creates an asymmetry with the seasonal-stochastic component forecast, which does incorporate the most recent observation x_n through the current z-score $\tilde{z}$_n in Equation (41). The paper cites Dagum & Bianconcini (reference [59]) on asymmetric window estimation techniques that could in principle make c_n and β_n available, but this technique is not applied. The authors should either justify explicitly why using c_{n - ℓ} and β_{n - ℓ} is preferable to asymmetric boundary estimation or test the alternative and compare accuracy.
Our answer: The reviewer is correct when observing that "at forecasting origin n, the most recent available trend-cycle information is 26 weeks old". We note, however, that this is not a particular aspect of our method, but also occurs in seasonal adjustment methods that use moving windows to perform time series decomposition. In fact, the techniques for handling asymmetric windows discussed in our reference [Dagum 2016] rely on forecasting assumptions regarding the future evolution of the trend-cycle in order to obtain endpoint estimates and can be interpreted as applying a symmetric filter to a series extended by implicit forecasts of the trend-cycle (see in [Dagum 2016], Chapter 7: "the assumption made is that the most recent values of the series (...) follow a constant linear trend", and in our newly added reference [Quenneville 2003]: "the X-11 trend-cycle asymmetric averages can now be expressed as the application of the symmetric Henderson average on the available series values extended with these implicit forecasts"). Doing that is justified when the seasonal adjustment is the primary objective and a trend-cycle estimate is required at every time point, including near the end of the time series. In contrast, our goal is to generate forecasts beyond the last available observation and we therefore group all operations involving forecasting in the Time series forecasting section. Consequently, the trend-cycle estimates used as inputs are restricted to those obtained from fully observed symmetric windows, preventing information derived from model-based forecasts from entering the decomposition stage before the forecasting procedure itself. We have added a note to the text: "techniques for seasonal adjustment in asymmetric windows exist [59], but they rely on forecasting assumptions regarding the future evolution of the trend-cycle component in order to obtain endpoint estimates [60] – we choose to keep the decomposition and forecasting tasks separate" (lines 297-301).
Regarding the first implication, the 26 most recent weeks are not discarded, they are included in the computation of the last available value c_{n - 26} and slope β_{n - 26} of the trend-cycle component, which are subsequently used for the prediction, as the reviewer pointed out. Consequently, the asymmetry mentioned in the second implication is only apparent: both the trend-cycle and seasonal-stochastic forecasts ultimately incorporate information from the most recent observations x_{n - 26}, ..., x_{n}.

4) Table 1 highlights the minimum value for each accuracy metric and each forecast horizon in bold. This visual emphasis implies that the highlighted method is meaningfully superior to its nearest competitor. However, as argued in the major comment 1, no formal significance test is conducted. For τ = 1, the differences between the proposed method and SARIMAX are so small relative to the standard deviations that the highlighted minima are statistically meaningless without supporting test results. The authors should either add significance test results to Table 1 or remove the bold highlighting and replace it with

a more cautious presentation.
Our answer: We agree that the bold highlighting may be misleading and have removed it.

5) The overcoverage at $\tau = 26$ (empirical coverage 0.923 vs. theoretical 0.85) is notable but receives only one sentence of discussion. Is this due to the trend uncertainty dominating at longer horizons? A decomposition of the interval width into trend and seasonal contributions would be informative.
Our answer: The intuition of the reviewer is supported by our additional analysis. In the added "S7 Appendix: (Log-)Normality assumption", we show that the contribution of the trend-cycle component to the total uncertainty steadily increases with the forecasting horizon and becomes dominant at longer horizons, while the uncertainty due to the seasonal-stochastic component increases only slightly. We write: "This finding is consistent with the overcoverage observed for long forecasting horizons and suggests that it is mainly associated with an overestimation of the trend-cycle forecast standard deviation. Further improvements in trend-cycle forecasting are thus expected to contribute to the calibration of long-horizon prediction intervals".

D) Adequacy and Appropriateness of Conclusions:
The central claim that the proposed method outperforms Prophet and SARIMAX/SARIMA in this case study is supported by the data as presented, particularly at longer horizons. However, several conclusions are stated too broadly:
Our answer: We agree that some conclusions could be stated more carefully. We address the concern below.

1) The abstract states accuracy "not reached by other comparable established seasonal models." This is too strong given the single-series evaluation and absence of significance testing.
Our answer: We softened the statement in the abstract: "accuracy levels comparable to or better than those of other established seasonal models, namely Prophet and SARIMAX, particularly at longer forecast horizons".

2) The conclusion that the method "outperformed standard models" should be qualified to reflect the specific dataset, parameter settings, and lack of statistical testing.
Our answer: Answered above (comment 2 in Major Comments).

3) The discussion of prediction intervals (Table 2) is largely descriptive. The overcoverage at $\tau = 26$ is noted but not explained or corrected for. This suggests the uncertainty model may be misspecified at longer horizons.
Our answer: Answered above (comment 5 in Minor Comments).

4) The conclusion section appropriately identifies limitations (intermittency, structural breaks, log-normality) but these should appear more prominently, as they are non-trivial for many practical demand forecasting applications.
Our answer: Answered above (comment 2 in Major Comments).

E) Clarity and Writing Quality:
The paper is well-organized and written in fluent English. The logical flow from data

description → decomposition → forecasting → empirical evaluation is clear. The use of a diagram (Fig. 3) to illustrate the iterative decomposition algorithm is particularly effective.

Our answer: We thank the reviewer for the positive comments on the organization and clarity of the manuscript. We are pleased that the logical flow and the diagram were found effective.

F) Ethics, Research Integrity, and Data Availability:
With regards of these topics, I only have these concerns:
1) The data are proprietary (Sony Group Corporation) and actual sales figures have been masked by an undisclosed scaling factor. This is acknowledged by the authors, but it raises concerns: (a) the results cannot be independently replicated; (b) the magnitude of actual errors is unknown to readers; and (c) it is unclear whether all figures and tables were reviewed by Sony Group Corporation for competitive sensitivity, which could introduce selective reporting risk.

Our answer: During the submission process, the Data Availability Statement and the Financial Disclosure – which address the reviewer's concerns – are submitted separately from the main manuscript and the reviewer may have overlooked them. Their current wording is reproduced below:

Data Availability Statement: "The masked data (see Data description section) and Python code to replicate the results of the proposed forecasting method are available at https://github.com/arthur-matsuo/tv-week-forecast."

Financial Disclosure: "This work was supported by Sony Group Corporation. Aside from providing data and suggesting a non-black box forecasting method, the funder had no further influence over the study design, data analysis, decision to publish, or preparation of the manuscript."

Regarding the data masking, the added "S1 Appendix: Data masking" further discusses the effect of such masking and provides evidence that it does not alter the results or conclusions of the analysis.

2) The paper should include a data availability statement explicitly noting that the data cannot be shared and explaining what can be made available (e.g., code).

Our answer: Answered above (comment 1 in Ethics, Research Integrity, and Data Availability).

3) The analogous week rules (Section "Analogous weeks") contain several subjective decisions (e.g., assignment of Amazon Prime Day 2014, similarity of COVID stimulus to Memorial Day, treatment of holiday overlaps). These are documented in the paper, which is commendable, but no sensitivity analysis tests whether alternative judgments would materially affect results.

Our answer: As suggested, we have included "S3 Appendix: Analogous weeks of irregular events" and "S4 Appendix: Holiday/event overlap", where we test alternative judgments and show that, while errors are the lowest for the choices presented in the main text, accuracy is not substantially affected under 'reasonable' alternative settings (e.g., excluding clearly dissimilar assignments such as treating Black Friday as a similar event).

4) No code repository is referenced. Given the methodological contribution, sharing the

implementation (even on an alternative or synthetic dataset) would substantially increase reproducibility and research integrity.
Our answer: Answered above (comment 1 in Ethics, Research Integrity, and Data Availability).

G) Adherence to Reporting Guidelines and Community Standards:
I have no major comments in regard of these topics.
Our answer: No comments from our side.

RECOMMENDATION AND REQUIRED REVISIONS:
It is my opinion that the paper makes a genuine, if incremental, methodological contribution and addresses a real practical problem with care, however, it cannot be accepted in its current form. I strongly suggest the author perform, at least, the following revisions:
Our answer: We thank the reviewer for recognizing the methodological contribution and practical relevance of our work. We have carefully revised the manuscript following the reviewer's comments, most of which have already been addressed above.

Required revisions:
1) Conduct formal statistical tests of forecast accuracy differences (Diebold-Mariano or equivalent) for all pairwise comparisons at all horizons. Revise claims of superiority accordingly.
Our answer: Answered above (comment 1 in Major Comments).

2) Evaluate the method on at least one additional publicly available weekly sales or demand dataset to support generalizability claims. If this is not possible (e.g., due to data constraints), the scope of conclusions must be radically narrowed to explicitly characterize this as a single-case-study with limited external validity.
Our answer: Answered above (comment 2 in Major Comments).

3) Provide a sensitivity analysis for parameters ℓ and λ, demonstrating the robustness of results to alternative choices.
Our answer: Answered above (comment 3 in Major Comments).

4) Provide diagnostics for the log-normality assumption (residual Q-Q plots, formal tests) and for the AR(1) order selection (ACF/PACF of z-scores, estimated $\hat{\rho}$ over rolling origins).
Our answer: Answered above (comment 5 and 7 in Major Comments).

5) Make code publicly available, even if applied to a synthetic or publicly available dataset, to ensure reproducibility.
Our answer: Answered above (comment 1 in Ethics, Research Integrity, and Data Availability).

Recommended revisions:
1) Discuss and analyze the asymmetry in parameter optimization between the proposed method and benchmarks, and assess its impact on the comparison.
Our answer: Answered above (comment 8 in Major Comments).

2) Add a robustness check for the COVID-19 period (e.g., results excluding 2020) and provide further analysis of the Black Friday overprediction pattern.
Our answer: Partially answered above (comment 6 in Major Comments). Regarding the Black Friday overprediction pattern, as noted in the manuscript, "with the available data, it is not clear yet if the overprediction is a systematic error of the proposed method or due to the small number of instances". Given that the dataset contains 11 Black Friday weeks, only 4 of which appear in the test period, we believe that additional data would be necessary to draw conclusions. To better quantify this limitation, we added the statement "(11 Black Friday weeks in total, 4 in the test period)" to the manuscript (line 428).

3) Unify and clarify notation throughout (especially the forecast horizon symbol and the intercept/slope symbols in the decomposition equations).
Our answer: Answered above (comment 5 in Major Comments).

References:
[1] Makridakis, S., Andersen, A., Carbone, R., Fildes, R., Hibon, M., Lewandowski, R., Newton, J., Parzen, E., & Winkler, R. (1982). The accuracy of extrapolation (time series) methods: Results of a forecasting competition. Journal of Forecasting, 1(2), 111-153. https://doi.org/10.1002/for.3980010202
[2] Makridakis, S., & Hibon, M. (2000). The M3-competition: Results, conclusions and implications. International Journal of Forecasting, 16(4), 451-476. https://doi.org/10.1016/S0169-2070(00)00057-1
[3] Makridakis, S., Spiliotis, E., & Assimakopoulos, V. (2020). The M4 competition: 100,000 time series and 61 forecasting methods. International Journal of Forecasting, 36(1), 54-74. https://doi.org/10.1016/j.ijforecast.2019.04.014
[4] Wolpert, D. H., & Macready, W. G. (1997). No free lunch theorems for optimization. IEEE Transactions on Evolutionary Computation, 1(1), 67-82. https://doi.org/10.1109/4235.585893
Our answer: We thank the reviewer for the references. We have added reference [1] to the Conclusion when commenting that "no method dominates universally across datasets".

Response to Reviewer #2

This manuscript proposes a time series decomposition method for forecasting weekly sales, using Sony TV sales in the United States (2014-2024) as a case study. The central contribution is the concept of "analogous weeks". This is a rule-based framework for grouping weeks that share similar calendar characteristics (holidays, events, or position within the year), which addresses irregular seasonality arising from variable year lengths and floating holidays. The method decomposes the log transformed sales into trend-cycle and seasonal-stochastic components, forecasts each independently, and recombines them. The authors find that the proposed approach achieves yearly average percentage errors below 10% for 1-week-ahead forecasts and below 15% for horizons up to 26 weeks, outperforming Prophet and SARIMAX benchmarks across multiple accuracy metrics.
The manuscript addresses a practically relevant problem with a conceptually clear and interpretable solution. The "analogous weeks" framework is a useful contribution that could benefit practitioners seeking transparent forecasting methods. However, the study has no-

table limitations regarding generalizability (a single product/company/country), reliance on subjective judgment to define analogous weeks, and insufficient statistical testing of performance differences.

Our answer: The reviewer accurately summarizes the content of our manuscript and we appreciate the recognition of the practical contribution of our work. We also appreciate the constructive comments regarding the study's limitations and address them point-by-point below.

Major comments

1. Limited generalizability and external validity

The study uses a single product category (TVs), single company (Sony), and single geographic market (United States). While the authors acknowledge this as a "case study," the claims about the method's superiority lack external validation. It cannot be assessed whether the approach generalizes to other products, markets, or industries with different demand patterns. The authors should explicitly discuss generalizability limitations in the Discussion section, ideally test the method on at least one additional publicly available dataset, or provide guidance on how practitioners should adapt the analogous weeks rules for different contexts.

Our answer: We agree with the reviewer that the evaluation based on a single product category, company and geographic market limits the empirical support for assessing the general applicability of the proposed method. However, our main objective is not to claim universal applicability of the proposed method, but rather to introduce the concept of 'analogous weeks' and to demonstrate, through a detailed case study, how it can be systematically incorporated into time series decomposition and forecasting to address irregular seasonality – while at the same time contributing to the discussion on simplicity and interpretability in forecasting methods.

Although evaluation on additional datasets would provide broader empirical evidence, the present study follows a case-study approach focused on introducing a methodological concept. Similar approaches using a single dataset have also been reported in the literature; for example, reference [34] analyzed SKU (Stock Keeping Unit) data from a 'manufacturing company specializing in household products' to 'show that judgmental adjustments can enhance baseline forecasts during promotions'; reference [46] analyzed Australian domestic tourism demand data to evaluate which hierarchical forecasting method is the most suitable for the specific Australian tourism market; and reference [57] analyzed France electricity demand data in a case study on load forecasting for France by introducing a rule-based methodology to improve forecasts for special days.

In our conclusion, we explicitly acknowledge that the method may require adaptation when the characteristics of the data differ substantially from those of our case study and added the statement: "However, no forecasting method consistently dominates across all datasets and the most appropriate approach depends on the characteristics of the data and forecasting problem [17,18]" (lines 561-563). Also, we comment on how the rules can be adapted, giving an example: "Naturally, this notion can be extended to other time units, but the rules to define the analogies need to be carefully defined in order to reflect the observed behavior in the time series. For example, if working with 'analogous days', weekly seasonality may play a significant role and it is then important to take the day of the week in consideration, e.g, the set of analogous days of a given Monday should only contain Mondays" (lines 555-559).

2. Subjective judgment in "analogous weeks" definition
The rules for defining analogous weeks (Section: Analogous weeks) involve substantial forecaster judgment, particularly for irregular events (e.g., "COVID-19 Economic Impact Payments similar to Memorial Day week"). While the authors state such judgments should be "clearly documented," this subjectivity undermines reproducibility and introduces potential bias. Impact: Different practitioners applying the method may obtain different forecasts depending on their judgment. The authors should provide sensitivity analyses showing how forecast accuracy changes under alternative analogous week assignments. Consider formalizing decision criteria or providing a decision tree to guide practitioners.
Our answer: The reviewer raises an important point and we agree that defining analogous weeks for irregular events relies on subjective judgment. Following the reviewer's suggestion, we added "S3 Appendix: Analogous weeks of irregular events", where we test alternative analogous week assignments for irregular events and show that, while errors are the lowest for the choices presented in the main text, accuracy is not substantially affected under reasonable settings (e.g., excluding clearly dissimilar assignments such as treating Black Friday as a similar event). Additionally, we included the following paragraph to further explain the suggested rules: "Rules for ordinary weeks, holidays and regular events are straightforward due to their yearly recurrence. For irregular events, the assignment of analogous weeks requires forecaster judgment. Similarity in event characteristics should be preferred when available (e.g., Amazon Prime Day 2 similar to Amazon Prime Day). When such information is not available or is ambiguous, temporal proximity should be used as a secondary criterion (e.g., COVID-19 Economic Impact Payments assumed similar to Memorial Day week because it is the nearest regular holiday with increase in sales and Best Buy Early Black Friday Deals assumed similar to Amazon Prime Day because it occurs in the week after Amazon Prime Day 2)" (lines 205-213).

3. Parameter selection lacks rigorous justification
The parameters $\ell = 26$ and $\lambda = 26$ are selected heuristically based on the "yearly periodicity" argument (lines 266–268, 339–341). No cross-validation, grid search, or sensitivity analysis is provided to justify these choices. It is recommended that the authors conduct sensitivity analyses across a range of ℓ and λ values (e.g., 13, 26, 39, 52) and report performance variation. Alternatively, implement a time-series cross-validation approach to select parameters within the training window.
Our answer: Following the reviewer's recommendation, we have added "S5 Appendix: Parameter ℓ of time series decomposition" and "S6 Appendix: Autoregressive correction for s_t forecasting", which contain sensitivity analyses for the parameters ℓ and λ. The results indicate that the proposed method is robust across a reasonable range of parameters and support $\ell = \lambda = 26$ as practical default values.

4. Statistical significance of performance differences not assessed
Table 1 reports means and standard deviations of accuracy metrics, but no statistical tests (e.g., Diebold-Mariano test, paired t-tests, or non-parametric alternatives) are conducted to determine whether differences between the proposed method and benchmarks are statistically significant. The authors should apply appropriate statistical tests for comparing forecast accuracy and report p-values or confidence intervals for performance differences.
Our answer: We follow the reviewer's suggestion and conduct the Diebold–Mariano test. The results, including the corresponding test statistics and p-values, are now reported in Table 2 and discussed in the revised manuscript. The statistical analysis supports the

main findings from the accuracy metrics and also provides a more nuanced interpretation of some comparisons. In the revised discussion, we clarify that the proposed method demonstrates competitive and statistically supported performance in most comparisons, while acknowledging that benchmark methods can achieve similar accuracy under specific conditions: "Overall, these results show that the competitive performance of the proposed method in this case study is not only reflected in lower error metrics but is also statistically supported in most comparisons. The benchmarks Prophet and SARIMAX produce forecasts with similar accuracy to the proposed method under specific conditions: when Prophet parameters are effectively fine-tuned and when SARIMAX is applied to short forecast horizons" (lines 515-520).

5. Handling of COVID-19 period raises concerns
The treatment of COVID-19 weeks as outliers (removed from training) and COVID-19 Economic Impact Payments as irregular events (analogous to Memorial Day) involves strong assumptions. The pandemic fundamentally altered consumer behavior in ways that may not match pre-pandemic patterns. Impact: The model's performance during and after the pandemic may be artificially inflated by excluding difficult-to-predict periods. The authors should provide separate accuracy metrics for pre-COVID (2014–2019) and post-COVID (2021–2024) periods. Discuss how the method would perform in real-time during unprecedented events when appropriate analogies are unavailable.
Our answer: We thank the reviewer for this comment. As noted in the manuscript, the outlier weeks were not selected by us but were directly informed by Sony based on known disruptions in production and supply. Although the COVID-19 outbreak period is not included in our test set (which begins in 2021) – which may indeed inflate the model's performance – all forecasting methods considered are evaluated using identical training and test sets, ensuring a fair comparison. Regarding the suggestion to separate accuracy metrics for the pre-COVID (2014–2019) and post-COVID (2021–2024) periods, such comparison is unfortunately not feasible because there is not enough data for the decomposition and forecasting in the pre-COVID period. This is the reason why our test period begins in 2021. The reviewer may find informative the added "S2 Appendix: COVID-19 outliers", which shows that the method is robust to moderate extensions in the COVID outlier period.
Under the definition presented in the manuscript, "outliers are only identified after they happened". Therefore, in a real-time application, an unexpected event such as the outbreak of the COVID-19 pandemic would initially be treated as an ordinary observation and forecasting accuracy would likely deteriorate until the event could be identified as an outlier and incorporated into the model. This limitation is not specific to the proposed method but is inherent to any forecasting approach that does not have prior knowledge of unprecedented events.

6. Convergence condition verification missing
The authors note (lines 289–290) that convergence requires the spectral radius of WAWL to be strictly less than 1, but state that "cases for which the convergence condition does not hold remain to be studied." It is unclear whether convergence was verified for the actual data analyzed. The authors should explicitly report the spectral radius computed for the analyzed data and confirm convergence.
Our answer: Indeed, this information was previously omitted. We confirm that convergence was checked for all decompositions performed in this study and added to the text

"the spectral radius of the corresponding $\boldsymbol{W_A W_L}$ was 0.019; the largest spectral radius across all decompositions performed in this study was 0.027" (lines 325-326).

7. Visual presentation and figure quality require substantial improvement
Several figures in the manuscript present formatting and accessibility issues that hinder reader comprehension. First, some figures do not appear to be properly formatted to fit within standard page dimensions, which may result in cropping or reduced legibility in the published version. Second, Figure 2 relies on color coding to distinguish different event types (e.g., regular events, irregular events, weeks removed from training), yet the figure itself lacks a self-contained legend explaining the meaning of each color. This information is provided only in the main text (lines 114–120), requiring readers to cross-reference between the figure and prose. Third, Figure 4 is not comprehensible in its current form (the visual elements, axes, or data representation are unclear, preventing adequate evaluation of the content it is meant to convey).
Our answer: We thank the reviewer for the comments regarding the figures. We first note that, during the submission process, figures were requested as separate files rather than embedded in the manuscript. As a result, each figure is displayed on a separate page, which affects its apparent size and layout. In the published version, the figures will be properly integrated into the text. Regarding Figure 2, we agree that a self-contained legend improves readability and have modified the figure accordingly. Concerning Figure 4, the figure is intended to visualize the structure of matrix $\boldsymbol{W}$ as a mathematical object, with colors representing the matrix coefficients. We have modified its caption for clarity: "Matrix $\boldsymbol{W}$ to obtain the logarithm of the trend-cycle component of the time series of weekly TV sales from 2014 to 2024 (574 weeks). The matrix has dimension 574×574 and colors represent the values of its coefficients."

Minor comments
1. Reference 2 (lines 512–514) appears distorted: "forecasting: Research R, practice. Fildes, Robert..." should be corrected to proper citation format.
Our answer: Corrected.

2. Line 47: "X-13ARIMA-SEATS" should include a brief explanation or citation upon first mention for readers unfamiliar with seasonal adjustment software.
Our answer: We cite [26] and [27] after first mentioning X-13ARIMA-SEATS. These references direct readers to the United States Census Bureau webpage on X-13ARIMA-SEATS, where detailed information can be found. We also added that X-13ARIMA-SEATS is a "seasonal adjustment" software to better characterize it.

3. Lines 99–100: The phrase "masked by dividing them by a factor q" would benefit from stating whether q is constant across time or how this masking affects variance properties.
Our answer: We modified the text to "masked by dividing them by a constant factor q" to clarify that the masking factor does not vary over time. In the added "S1 Appendix: Data masking", we further discuss the effect of this masking and provide evidence that it does not alter the results or conclusions of the analysis.

4. Table 1: Consider adding a row showing the percentage improvement of the proposed method over the best competitor for easier interpretation.
Our answer: We thank the reviewer for this suggestion. While we agree that a summary

measure could be informative, Table 1 already provides all absolute results required to compute the percentage improvement. To avoid redundancy and preserve readability of the table, we opted not to include an additional row.

5. The prediction interval analysis (Table 2) would benefit from reporting calibration metrics (e.g., PIT histograms) beyond coverage and width.
Our answer: We further discuss the calibration of prediction intervals in the added "S7 Appendix: (Log-)Normality assumption", which includes additional analyses based on the distribution of forecast errors.

6. Line 393: "1.440, 1.645 and 1.960 standard deviations" should clarify these correspond to approximately 85%, 90%, and 95% for two-tailed normal intervals.
Our answer: We modified the text to "respectively corresponding to theoretical coverages of approximately 85%, 90% and 95% under the assumed normal model (two-sided intervals)".
