# Supplementary material for: Weekly sales forecasting using time series decomposition with irregular seasonality: A case study of Sony TV sales in the United States
Source: PLoS One. 2026 Nov 3;21(11):e0344222. doi: 10.1371/journal.pone.0344222 (PMC13730381; doi:10.1371/journal.pone.0344222)
Supplement: Peer review history

# Peer review: Decision Letter 0

**DOI:** 10.1371/journal.pone.0344222.r001

**Authors:** Szabolcs Blazsek

26 May 2026

Dear Dr. Yamashita Rios de Sousa,

Thank you for submitting your manuscript to PLOS ONE. After careful consideration, we feel that it has merit but does not fully meet PLOS ONE’s publication criteria as it currently stands. Therefore, we invite you to submit a revised version of the manuscript that addresses the points raised during the review process.

Please submit your revised manuscript by Jul 10 2026 11:59PM. If you will need more time than this to complete your revisions, please reply to this message or contact the journal office at plosone@plos.org. When you're ready to submit your revision, log on to https://www.editorialmanager.com/pone/ and select the 'Submissions Needing Revision' folder to locate your manuscript file.

- A letter that responds to each point raised by the academic editor and reviewer(s). You should upload this letter as a separate file labeled 'Response to Reviewers'.
- A marked-up copy of your manuscript that highlights changes made to the original version. You should upload this as a separate file labeled 'Revised Manuscript with Track Changes'.
- An unmarked version of your revised paper without tracked changes. You should upload this as a separate file labeled 'Manuscript'.

If you would like to make changes to your financial disclosure, please include your updated statement in your cover letter. Guidelines for resubmitting your figure files are available below the reviewer comments at the end of this letter.

If applicable, we recommend that you deposit your laboratory protocols in protocols.io to enhance the reproducibility of your results. Protocols.io assigns your protocol its own identifier (DOI) so that it can be cited independently in the future. For instructions see: https://journals.plos.org/plosone/s/submission-guidelines#loc-laboratory-protocols. Additionally, PLOS ONE offers an option for publishing peer-reviewed Lab Protocol articles, which describe protocols hosted on protocols.io. Read more information on sharing protocols at https://plos.org/protocols?utm_medium=editorial-email&utm_source=authorletters&utm_campaign=protocols.

As the corresponding author, your ORCID iD is verified in the submission system and will appear in the published article. PLOS supports the use of ORCID, and we encourage all coauthors to register for an ORCID iD and use it as well. Please encourage your coauthors to verify their ORCID iD within the submission system before final acceptance, as unverified ORCID iDs will not appear in the published article. *Only*  the individual author can complete the verification step; PLOS staff *cannot*  verify ORCID iDs on behalf of authors.

We look forward to receiving your revised manuscript.

Kind regards,

Szabolcs Blazsek

Academic Editor

PLOS One

Journal Requirements:

When submitting your revision, we need you to address these additional requirements.

1. Please ensure that your manuscript meets PLOS ONE's style requirements, including those for file naming. The PLOS ONE style templates can be found at

https://journals.plos.org/plosone/s/file?id=wjVg/PLOSOne_formatting_sample_main_body.pdf and

https://journals.plos.org/plosone/s/file?id=ba62/PLOSOne_formatting_sample_title_authors_affiliations.pdf

2. In your Methods section, please include additional information about your dataset and ensure that you have included a statement specifying whether the collection and analysis method complied with the terms and conditions for the source of the data.

3.  Thank you for stating the following in the Financial Disclosure section: [This work was supported by Sony Group Corporation. Aside from providing data and suggesting a non-black box forecasting method, the funder had no further influence over the development of the research.].

We note that you received funding from a commercial source: [Sony Group Corporation]

Please provide an amended Competing Interests Statement that explicitly states this commercial funder, along with any other relevant declarations relating to employment, consultancy, patents, products in development, marketed products, etc.

Within this Competing Interests Statement, please confirm that this does not alter your adherence to all PLOS ONE policies on sharing data and materials by including the following statement: ""This does not alter our adherence to PLOS ONE policies on sharing data and materials.” (as detailed online in our guide for authors http://journals.plos.org/plosone/s/competing-interests).  If there are restrictions on sharing of data and/or materials, please state these. Please note that we cannot proceed with consideration of your article until this information has been declared.

Please include your amended Competing Interests Statement within your cover letter. We will change the online submission form on your behalf.

4. If the reviewer comments include a recommendation to cite specific previously published works, please review and evaluate these publications to determine whether they are relevant and should be cited. There is no requirement to cite these works unless the editor has indicated otherwise.

Reviewers' comments:

Reviewer's Responses to Questions

**Comments to the Author**

1. Is the manuscript technically sound, and do the data support the conclusions?

Reviewer #1: Partly

Reviewer #2: Yes

**********

2. Has the statistical analysis been performed appropriately and rigorously?

Reviewer #1: No

Reviewer #2: Yes

**********

3. Have the authors made all data underlying the findings in their manuscript fully available?

The PLOS Data policy

Reviewer #1: No

Reviewer #2: Yes

**********

4. Is the manuscript presented in an intelligible fashion and written in standard English?

Reviewer #1: Yes

Reviewer #2: Yes

**********

**Reviewer #1: Review of the paper (this review was also uploaded as an attachment):**

Weekly sales forecasting using time series decomposition with irregular seasonality: A

case study of Sony TV sales in the United States

The authors argue that they present in the paper “…a simple and interpretable forecasting method for weekly sales…”. They argue that the main challenge “…is to properly address irregular seasonality arising from the way a year is divided into weeks and from floating holidays and events…”. The authors propose the concept of “analogous weeks”, which is described in the paper as “… a general framework for grouping weeks that are similar according to some criteria, which encodes seasonality and already accounts for calendar effects.”. They also argue that, incorporating the proposed concept into the time series decomposition and forecasting of the case study, they “…achieved yearly average percentage errors of less than 10% for the 1-week-ahead forecast and less than 15% for forecast horizons of up to 26 weeks, accuracy levels not reached by other comparable established seasonal models, namely Prophet and SARIMAX.”.

A) Originality of Research

It is my criteria that the level of originality of research is satisfactory, but I have these comments:

The analogous weeks concept is the paper's primary intellectual contribution. The authors correctly situate it relative to prior work on historical analogues (used mainly for special events), arguing that their framework generalizes the idea to all weeks systematically. This is a defensible and useful extension. The matrix formulation of the decomposition (Eqs. 16–24) is a noteworthy theoretical contribution: it shows that the decomposition reduces to linear transformations involving the matrix W (specifically Wx for the trend-cycle component and (I−W)x for the seasonal-stochastic component) where W depends only on the calendar and outlier structure, not on the data themselves. This has useful implications for multi-series applications.

However, several concerns limit the novelty claim:

1) The decomposition procedure, according with the paper (section “Proposed decomposition”) is related to X-11-ARIMA and STL methods. The main contribution of the paper is the replacement of a fixed-period seasonal averaging with analogous-week averaging, which may be an incremental value added to previous methodologies.

2) The paper references Arora and Taylor (references [55, 56] of the paper). The authors should clearly establish what is added to these methods in the proposed analogous weeks methodology.

3) There is no contribution to the forecasting model for the trend-cycle component described on equation 25 as it is a standard OLS regression on the lagged trend level and slope.

4) The authors do not discuss if this concept has been suggested (even by a different name, or in a partial or modified way) in proprietary industry practice, where such calendar-based grouping is common in retail forecasting.

B) Results Not Published Elsewhere:

I found no evidence of prior publication. The methodology appears original to this submission. The dataset is proprietary of Sony Group Corporation.

C) Technical Standards and Methodological Rigor:

The technical contribution of the paper is the analogous weeks concept.

The matrix formulation of the decomposition (in equations 16-24) is a valuable theoretical contribution because it shows that the decompositions reduce to a single linear transformation that depends only on the calendar and outlier structure and not on the data themselves. This formulation may have useful implications for multi-series applications. However, I have several concerns about their methodology.

Major Comments:

1) The comparisons between the proposed method and benchmarks rely entirely on point estimates of accuracy metrics (MAE, RSME, MAPE, WMAPE, and SMAPE Table 1, Fig. 10). Point estimates alone do not tell whether observed differences between models are statistically meaningful or merely due to sampling variability. No statistical tests of forecast accuracy differences are conducted. Even when the differences in some cases seems visually clear, in some cases may not be statistically significant, particularly for SARIMAX at τ = 1 (e.g., proposed method shows 1.194 vs. SARIMAX 1.216, with a standard deviations of 0.350 and 0.396, respectively, the difference is 0.022 which imply that this difference of is approximately 1/16 of one standard deviation). Authors should consider the use of Diebold-Mariano test or the modified Harvey-Leybourne-Newbold version for the pairwise comparisons. In this issue, maybe the authors may wish to consider to acknowledge that for τ = 1 is essentially a tie with SARIMAX, and arguing that the contribution of the paper (at least with for the case presented in the paper) is centered in medium-to-longer horizon stability.

2) The entire empirical evaluation rests on one time series (total weekly Sony TV unit sales in the United States) spanning 574 weeks (2014–2024). Every accuracy number in Table 1, every curve in Figures 7–10, and every coverage figure in Table 2 derives exclusively from this single series. The conclusion that the proposed method "outperformed standard models" is drawn solely from this evidence base. The forecasting literature has established that no method dominates universally across datasets (see, e.g., Makridakis, S. et. al, 1982, 2000, 2020, and Wolpert, D. H., & Macready, W. G. (1997). It is impossible to determine from a single series whether the proposed method's apparent superiority is robust or specific to this dataset's characteristics. The authors should either, evaluate on additional publicly available retail or weekly sales datasets, or substantially moderate the generalizability of their conclusions.

3) The paper states that two key parameters (λ = ℓ = 26) are selected heuristically, justified by the approximate annual period. No sensitivity analysis is conducted. Sensitivity analysis is required.

4) The paper established that equation (46) assumes independence (between the trend-cycle and seasonal-stochastic forecast errors) to compute the combined standard deviation of the log-sales forecast. This assumption is not tested empirically and may not hold, potentially leading to miscalibrated prediction intervals.

5) The paper models the z-scores z_t (defined in Equation (34)) with an AR(1) process: z_t = ρz_{t-1} + η ϕ_t where ρ is the autoregressive coefficient (estimated as ρ̂ in Equation (38)), η is the innovation standard deviation (estimated as η̂ in Equation (39)), and ϕ_t is i.i.d. N(0,1). The choice of order one for this autoregressive model is not empirically motivated. No ACF or PACF analysis of the z-scores is provided to justify this order for the Sony TV dataset. Furthermore, the estimated value of ρ̂ and its behavior across rolling origins are never reported. This matters for two reasons:

a) If ρ̂ is near zero across most rolling windows, the AR(1) correction contributes negligibly to forecast accuracy, and the added complexity is unnecessary. The simpler forecast s̃_{n+τ} = s̄_{n+τ} would suffice.

b) If ρ̂ is near 1, the stationarity of the z-score process is questionable, which would invalidate the AR(1) model assumptions underlying Equations (35) – (44).

Additionally, there is a notational “conflict” in the paper that should be resolved: the symbol ρ is used for two entirely different quantities in close proximity:

a) ρ^(τ) in Equation (25): the standard deviation of the trend-cycle forecast error (a scaling parameter).

b) ρ in Equations (35) and (38): the AR(1) autoregressive coefficient for the z-score process.

These are conceptually unrelated quantities, and their shared symbol is a source of genuine confusion, particularly because both appear in the forecasting section. The recommended resolution is to replace ρ^(τ) in Equation (25) with σ^(τ), which is consistent with the standard deviation notation used elsewhere in the paper (σ^(c), σ^(s), σ^(x), σ^(z) in Eqs. (31), (42), (44), (46)).

6) The authors of the paper removed 4 weeks, March to April 2020, because they considered them as outliers. The authors argue that the COVID-19 stimulus payments are treated as irregular events. However, the COVID-19 period involved not only demand shocks but also structural supply disruptions that likely persisted. The boundary between the outlier weeks and the subsequent recovery period is not clearly defined and its impact on the estimated decomposition is not assessed. Therefore, a robustness check excluding 2020 entirely (or a several months period after April 2020) may strengthen the analysis.

7) The prediction intervals rely on log-normality of sales, however, no diagnostic (e.g., Shapiro-Wilk or Jarque-Bera test on residuals) is provided. The authors acknowledge in the conclusions that large deviations from the (log-)normality assumption could be problematic but offer no evidence on where their dataset stands.

8) For the proposed method, ℓ and λ are fixed at 26 throughout all rolling origins (not optimized per origin). For Prophet Fine-Tuned, a grid search is performed at each origin. For SARIMA/SARIMAX, auto_arima selects parameters at each origin. This creates a comparison asymmetry: the proposed method uses fixed hyper-parameters while competitors use adaptive ones. The paper should either optimize ℓ and λ adaptively (consistent with competitors) or fix the competitors' parameters analogously and discuss what this choice implies for the comparison.

Minor comments:

1) Page 5, item (c): The judgment that "Best Buy Early Black Friday Deals" is similar to "Amazon Prime Day week" is non-obvious and deserves more explicit justification beyond the term "temporal proximity."

2) Page 9, Equation (25): As it was mentioned previously (major comment 5 of this review), the use of the symbol ρ is used for two entirely different quantities:

a) ρ^(τ) in Equation (25): the standard deviation of the trend-cycle forecast error, a scaling parameter for the error term ξ_{t+τ}.

b) ρ in Equation (35) and ρ̂ in Equation (38): the AR(1) autoregressive coefficient for the z-score process z_t.

Both quantities appear in the forecasting section in close proximity, and their shared symbol constitutes a genuine notational conflict that will confuse readers. The recommended resolution is to replace ρ^(τ) in Equation (25) with σ^(τ), consistent with the standard deviation symbols used throughout the uncertainty quantification section: σ^(c){n + τ} in Equation (31), σ^(z){n + τ} in Equation (42), σ^(s){n + τ} in Equation (44), and σ^(x){n + τ} in Equation (46).

3) The trend-cycle forecasting regression in Equation (25): c_{t + τ} = b^(τ)_0 + b^(τ)_1 · c_{t − ℓ} + b^(τ)_2 · β_{t − ℓ} + ρ^(τ) · ξ_{t + τ} uses c_{t − ℓ} and β_{t − ℓ} (the trend-cycle level and local linear slope both lagged ℓ = 26 weeks from the current time point t) as regressors. This occurs because the decomposition defines c_t and β_t only within the set U_ℓ= [1 + ℓ, n − ℓ], meaning these quantities are unavailable for the most recent ℓ weeks of the training set due to the symmetric window requirement of the local linear regression in Equation (8). The consequence is that at forecasting origin n, the most recent available trend-cycle information is 26 weeks old.

This is a non-trivial design choice with two implications the paper does not discuss. First, for the 1-week-ahead forecast (τ = 1), the model predicts c_{n + 1} using c_{n − 26} and β_{n − 26}, discarding 26 weeks of the most recent trend information. Second, this creates an asymmetry with the seasonal-stochastic component forecast, which does incorporate the most recent observation x_n through the current z-score z̃_n in Equation (41). The paper cites Dagum & Bianconcini (reference [59]) on asymmetric window estimation techniques that could in principle make c_n and β_n available, but this technique is not applied. The authors should either justify explicitly why using c_{n − ℓ} and β_{n − ℓ} is preferable to asymmetric boundary estimation or test the alternative and compare accuracy.

4) Table 1 highlights the minimum value for each accuracy metric and each forecast horizon in bold. This visual emphasis implies that the highlighted method is meaningfully superior to its nearest competitor. However, as argued in the major comment 1, no formal significance test is conducted. For τ = 1, the differences between the proposed method and SARIMAX are so small relative to the standard deviations that the highlighted minima are statistically meaningless without supporting test results. The authors should either add significance test results to Table 1 or remove the bold highlighting and replace it with a more cautious presentation.

5) The overcoverage at τ = 26 (empirical coverage 0.923 vs. theoretical 0.85) is notable but receives only one sentence of discussion. Is this due to the trend uncertainty dominating at longer horizons? A decomposition of the interval width into trend and seasonal contributions would be informative.

D) Adequacy and Appropriateness of Conclusions:

The central claim that the proposed method outperforms Prophet and SARIMAX/SARIMA in this case study is supported by the data as presented, particularly at longer horizons. However, several conclusions are stated too broadly:

1) The abstract states accuracy "not reached by other comparable established seasonal models." This is too strong given the single-series evaluation and absence of significance testing.

2) The conclusion that the method "outperformed standard models" should be qualified to reflect the specific dataset, parameter settings, and lack of statistical testing.

3) The discussion of prediction intervals (Table 2) is largely descriptive. The overcoverage at τ = 26 is noted but not explained or corrected for. This suggests the uncertainty model may be misspecified at longer horizons.

4) The conclusion section appropriately identifies limitations (intermittency, structural breaks, log-normality) but these should appear more prominently, as they are non-trivial for many practical demand forecasting applications.

E) Clarity and Writing Quality:

The paper is well-organized and written in fluent English. The logical flow from data description → decomposition → forecasting → empirical evaluation is clear. The use of a diagram (Fig. 3) to illustrate the iterative decomposition algorithm is particularly effective.

F) Ethics, Research Integrity, and Data Availability:

With regards of these topics, I only have these concerns:

1) The data are proprietary (Sony Group Corporation) and actual sales figures have been masked by an undisclosed scaling factor. This is acknowledged by the authors, but it raises concerns: (a) the results cannot be independently replicated; (b) the magnitude of actual errors is unknown to readers; and (c) it is unclear whether all figures and tables were reviewed by Sony Group Corporation for competitive sensitivity, which could introduce selective reporting risk.

2) The paper should include a data availability statement explicitly noting that the data cannot be shared and explaining what can be made available (e.g., code).

3) The analogous week rules (Section "Analogous weeks") contain several subjective decisions (e.g., assignment of Amazon Prime Day 2014, similarity of COVID stimulus to Memorial Day, treatment of holiday overlaps). These are documented in the paper, which is commendable, but no sensitivity analysis tests whether alternative judgments would materially affect results.

4) No code repository is referenced. Given the methodological contribution, sharing the implementation (even on an alternative or synthetic dataset) would substantially increase reproducibility and research integrity.

G) Adherence to Reporting Guidelines and Community Standards:

I have no major comments in regard of these topics.

RECOMMENDATION AND REQUIRED REVISIONS:

It is my opinion that the paper makes a genuine, if incremental, methodological contribution and addresses a real practical problem with care, however, it cannot be accepted in its current form. I strongly suggest the author perform, at least, the following revisions:

Required revisions:

1) Conduct formal statistical tests of forecast accuracy differences (Diebold-Mariano or equivalent) for all pairwise comparisons at all horizons. Revise claims of superiority accordingly.

2) Evaluate the method on at least one additional publicly available weekly sales or demand dataset to support generalizability claims. If this is not possible (e.g., due to data constraints), the scope of conclusions must be radically narrowed to explicitly characterize this as a single-case-study with limited external validity.

3) Provide a sensitivity analysis for parameters ℓ and λ, demonstrating the robustness of results to alternative choices.

4) Provide diagnostics for the log-normality assumption (residual Q-Q plots, formal tests) and for the AR(1) order selection (ACF/PACF of z-scores, estimated ρ̂ over rolling origins).

5) Make code publicly available, even if applied to a synthetic or publicly available dataset, to ensure reproducibility.

Recommended revisions:

1) Discuss and analyze the asymmetry in parameter optimization between the proposed method and benchmarks, and assess its impact on the comparison.

2) Add a robustness check for the COVID-19 period (e.g., results excluding 2020) and provide further analysis of the Black Friday overprediction pattern.

3) Unify and clarify notation throughout (especially the forecast horizon symbol and the intercept/slope symbols in the decomposition equations).

References:

[1] Makridakis, S., Andersen, A., Carbone, R., Fildes, R., Hibon, M., Lewandowski, R., Newton, J., Parzen, E., & Winkler, R. (1982). The accuracy of extrapolation (time series) methods: Results of a forecasting competition. Journal of Forecasting, 1(2), 111–153. https://doi.org/10.1002/for.3980010202

[2] Makridakis, S., & Hibon, M. (2000). The M3-competition: Results, conclusions and implications. International Journal of Forecasting, 16(4), 451–476. https://doi.org/10.1016/S0169-2070(00)00057-1

[3] Makridakis, S., Spiliotis, E., & Assimakopoulos, V. (2020). The M4 competition: 100,000 time series and 61 forecasting methods. International Journal of Forecasting, 36(1), 54–74. https://doi.org/10.1016/j.ijforecast.2019.04.014

[4] Wolpert, D. H., & Macready, W. G. (1997). No free lunch theorems for optimization. IEEE Transactions on Evolutionary Computation, 1(1), 67–82. https://doi.org/10.1109/4235.585893

**Reviewer #2** : The manuscript addresses a practically relevant problem with a conceptually clear and interpretable solution. The “analogous weeks” framework is a useful contribution that could benefit practitioners seeking transparent forecasting methods. However, the study has notable limitations regarding generalizability (a single product/company/country), reliance on subjective judgment to define analogous weeks, and insufficient statistical testing of performance differences. **Please see attached document.**

**********

what does this mean?). If published, this will include your full peer review and any attached files.

If you choose “no”, your identity will remain anonymous but your review may still be made public.

**Do you want your identity to be public for this peer review?**  For information about this choice, including consent withdrawal, please see our Privacy Policy

Reviewer #1: No

Reviewer #2: No

**********

[NOTE: If reviewer comments were submitted as an attachment file, they will be attached to this email and accessible via the submission site. Please log into your account, locate the manuscript record, and check for the action link "View Attachments". If this link does not appear, there are no attachment files.]

To ensure your figures meet our technical requirements, please review our figure guidelines: https://journals.plos.org/plosone/s/figures

You may also use PLOS’s free figure tool, NAAS, to help you prepare publication quality figures: https://journals.plos.org/plosone/s/figures#loc-tools-for-figure-preparation.

NAAS will assess whether your figures meet our technical requirements by comparing each figure against our figure specifications.

Attachment Submitted filename: Review of forecasting using time series decomposition.pdf

Attachment Submitted filename: PONE-D-26-08207.pdf

# Author comment: Author response to Decision Letter 1

**DOI:** 10.1371/journal.pone.0344222.r002

22 Jul 2026

Response to reviewers can be found in the file 'response_letter.pdf'.

Regarding the Journal Requirements:

1. About our dataset, we have added to our Data description section: “The data was provided by Sony Group Corporation under an agreement permitting its use for research purposes. In compliance with the terms and conditions established by the data provider, note that the actual sales numbers were masked by dividing them by a constant factor q; we use the masked data throughout this study”.

2. Amended Financial Disclosure:

“This work was supported by Sony Group Corporation. Aside from providing data and suggesting a non-black box forecasting method, the funder had no further influence over the study design, data analysis, decision to publish, or preparation of the manuscript.”

3. Amended Competing Interests Statement:

“Sony Group Corporation provided funding and data for this work. The authors declare no additional competing interests, including employment, consultancy, patents, products in development, or marketed products. This does not alter our adherence to PLOS ONE policies on sharing data and materials.”

4. Amended Data Availability Statement (wording only; available data remains unchanged):

“The masked data (see Data description section) and Python code to replicate the results of the proposed forecasting method are available at https://github.com/arthur-matsuo/tv-week-forecast.”

Attachment Submitted filename: response_letter.pdf

# Peer review: Decision Letter 1

**DOI:** 10.1371/journal.pone.0344222.r003

**Authors:** Szabolcs Blazsek

7 Sep 2026

Dear Dr. Yamashita Rios de Sousa,

Thank you for submitting your manuscript to PLOS One. After careful consideration, we feel that it has merit but does not fully meet PLOS One’s publication criteria as it currently stands. Therefore, we invite you to submit a revised version of the manuscript that addresses the points raised during the review process.

Please submit your revised manuscript by Oct 22 2026 11:59PM. If you will need more time than this to complete your revisions, please reply to this message or contact the journal office at plosone@plos.org. When you're ready to submit your revision, log on to https://www.editorialmanager.com/pone/ and select the 'Submissions Needing Revision' folder to locate your manuscript file.

- A letter that responds to each point raised by the academic editor and reviewer(s). You should upload this letter as a separate file labeled 'Response to Reviewers'.
- A marked-up copy of your manuscript that highlights changes made to the original version. You should upload this as a separate file labeled 'Revised Manuscript with Track Changes'.
- An unmarked version of your revised paper without tracked changes. You should upload this as a separate file labeled 'Manuscript'.

If you would like to make changes to your financial disclosure, please include your updated statement in your cover letter. Guidelines for resubmitting your figure files are available below the reviewer comments at the end of this letter.

If applicable, we recommend that you deposit your laboratory protocols in protocols.io to enhance the reproducibility of your results. Protocols.io assigns your protocol its own identifier (DOI) so that it can be cited independently in the future. For instructions see: https://journals.plos.org/plosone/s/submission-guidelines#loc-laboratory-protocols. Additionally, PLOS One offers an option for publishing peer-reviewed Lab Protocol articles, which describe protocols hosted on protocols.io. Read more information on sharing protocols at https://plos.org/protocols?utm_medium=editorial-email&utm_source=authorletters&utm_campaign=protocols.

As the corresponding author, your ORCID iD is verified in the submission system and will appear in the published article. PLOS supports the use of ORCID, and we encourage all coauthors to register for an ORCID iD and use it as well. Please encourage your coauthors to verify their ORCID iD within the submission system before final acceptance, as unverified ORCID iDs will not appear in the published article. *Only*  the individual author can complete the verification step; PLOS staff *cannot*  verify ORCID iDs on behalf of authors.

We look forward to receiving your revised manuscript.

Kind regards,

Szabolcs Blazsek

Academic Editor

PLOS One

Journal Requirements:

1. If the reviewer comments include a recommendation to cite specific previously published works, please review and evaluate these publications to determine whether they are relevant and should be cited. There is no requirement to cite these works unless the editor has indicated otherwise.

Additional Editor Comments:

Dear authors,

One reviewer recommends a major revision, while the other recommends rejecting the paper.

Both reviewers provided several constructive comments on the current version of the paper.

I believe the authors can use those comments to enhance the work and resubmit it to PLOS ONE.

Please see the comments of both reviewers and address them.

After the major revision, the authors may resubmit the paper.

[Note: HTML markup is below. Please do not edit.]

Reviewer's Responses to Questions

**Comments to the Author**

Reviewer #3: (No Response)

Reviewer #4: (No Response)

**********

2. Is the manuscript technically sound, and do the data support the conclusions?

Reviewer #3: Yes

Reviewer #4: No

**********

3. Has the statistical analysis been performed appropriately and rigorously?

Reviewer #3: Yes

Reviewer #4: No

**********

4. Have the authors made all data underlying the findings in their manuscript fully available?

The PLOS Data policy

Reviewer #3: Yes

Reviewer #4: (No Response)

**********

5. Is the manuscript presented in an intelligible fashion and written in standard English?

Reviewer #3: Yes

Reviewer #4: (No Response)

**********

Reviewer #3:

The paper proposes a simple way to forecast weekly TV sales for Sony in the US. Instead of comparing each week only to the same week last year, it groups weeks by similarity (‘analogous weeks’): weeks with the same holiday or event are compared to each other, even if their calendar week number differs. The sales series is split into a trend and a seasonal/random part, each part is forecast on its own, and the two are added back together. Tested against Prophet and SARIMAX, the method performs as well or better, especially for longer forecast horizons.

The idea is useful and the writing is clear, but several points still need attention:

1. All results come from a single product (TVs), single company (Sony), single country (US). This makes it hard to know if the method works well in general or only for this particular case. I propose to test the method on at least one more, ideally public, dataset, or state more clearly that the conclusions apply only to this case.

2. The paper never shows how much of the improvement comes specifically from grouping weeks by ‘analogy’ rather than from the rest of the method (trend fitting, AR(1) correction, etc.). The authors could run the same method but with plain fixed-week grouping instead of analogous weeks, and compare, to prove that the main idea itself is what helps.

3. For new or unusual events (e.g. COVID stimulus payments), the paper simply says by example which past week they resemble, without a clear general rule a practitioner could reuse for a brand-new event. One short, general rule, would help. For example, same event first, then a documented similar event, then simply the closest in time.

4. Outlier weeks are removed based on private information from Sony that readers cannot verify. Confirm in the text that this list was fixed before the results were calculated, so it was not adjusted afterward to improve accuracy.

5. Sales are divided by a hidden scaling factor, so absolute error numbers (MAE, RMSE) are not really comparable outside this paper; only percentage-based errors (MAPE, WMAPE, SMAPE) are. State this clearly in the main text, not only in the appendix.

6. Key supporting results — how sensitive the method is to its two parameters, and why long-horizon (26-week) intervals turn out wider than needed — are discussed only in supplementary appendices, even though they affect the main conclusions. A short summary of both in the main text would be good.

7. The proposed method always uses the same fixed hyperparameters, while Prophet and SARIMAX are re-tuned for every single forecast. This may make the proposed method look more stable and faster than it would be under equal conditions. I would recommend to mention this difference honestly when claiming speed and stability advantages.

8. The significance tests (Table 2) show that for several forecast horizons, the proposed method is not significantly better than SARIMAX or fine-tuned Prophet. State plainly how many comparisons were actually significant, instead of a general ‘comparable to or better than’ statement.

9. Black Friday forecasts have large errors, likely because there are only 11 such weeks in the whole dataset. A short practical note on how to handle these low-data, high-variance weeks in real use would help, for example by using wider intervals.

This is a clear, well-motivated paper with a useful practical idea. The main things still missing are: proof that the analogous-weeks idea itself (not just the surrounding method) drives the improvement, testing beyond a single dataset, and moving some important checks from the appendices into the main text. I recommend another round of revision focused on these points.

Reviewer #4: (No Response)

**********

what does this mean?). If published, this will include your full peer review and any attached files.

If you choose “no”, your identity will remain anonymous but your review may still be made public.

**Do you want your identity to be public for this peer review?**  For information about this choice, including consent withdrawal, please see our Privacy Policy

Reviewer #3: No

Reviewer #4: No

**********

[NOTE: If reviewer comments were submitted as an attachment file, they will be attached to this email and accessible via the submission site. Please log into your account, locate the manuscript record, and check for the action link "View Attachments". If this link does not appear, there are no attachment files.]

To ensure your figures meet our technical requirements, please review our figure guidelines: https://journals.plos.org/plosone/s/figures

You may also use PLOS’s free figure tool, NAAS, to help you prepare publication quality figures: https://journals.plos.org/plosone/s/figures#loc-tools-for-figure-preparation.

NAAS will assess whether your figures meet our technical requirements by comparing each figure against our figure specifications.

Attachment Submitted filename: round01.pdf

# Author comment: Author response to Decision Letter 2

**DOI:** 10.1371/journal.pone.0344222.r004

13 Oct 2026

Please find our responses to reviewers in the file 'response_letter.pdf'.

# Editor report: Decision Letter 2

**DOI:** 10.1371/journal.pone.0344222.r005

**Authors:** Szabolcs Blazsek

14 Oct 2026

Weekly sales forecasting using time series decomposition with irregular seasonality: A case study of Sony TV sales in the United States

PONE-D-26-08207R2

Dear Dr. Yamashita Rios de Sousa,

We’re pleased to inform you that your manuscript has been judged scientifically suitable for publication and will be formally accepted for publication once it meets all outstanding technical requirements.

Within one week, you’ll receive an e-mail detailing the required amendments. When these have been addressed, you’ll receive a formal acceptance letter and your manuscript will be scheduled for publication.

An invoice will be generated when your article is formally accepted. Please note, if your institution has a publishing partnership with PLOS and your article meets the relevant criteria, all or part of your publication costs will be covered. Please make sure your user information is up-to-date by logging into Editorial Manager at Editorial Manager® and clicking the ‘Update My Information' link at the top of the page. For questions related to billing, please contact billing support.

If your institution or institutions have a press office, please notify them about your upcoming paper to help maximize its impact. If they’ll be preparing press materials, please inform our press team as soon as possible -- no later than 48 hours after receiving the formal acceptance. Your manuscript will remain under strict press embargo until 2 pm Eastern Time on the date of publication. For more information, please contact onepress@plos.org.

Kind regards,

Szabolcs Blazsek

Academic Editor

PLOS One

# Editor report: Acceptance letter

**DOI:** 10.1371/journal.pone.0344222.r006

**Authors:** Szabolcs Blazsek

PONE-D-26-08207R2

PLOS One

Dear Dr. Yamashita Rios de Sousa,

I'm pleased to inform you that your manuscript has been deemed suitable for publication in PLOS One. Congratulations! Your manuscript is now being handed over to our production team.

At this stage, our production department will prepare your paper for publication. This includes ensuring the following:

* All references, tables, and figures are properly cited

* All relevant supporting information is included in the manuscript submission,

* There are no issues that prevent the paper from being properly typeset

You will receive further instructions from the production team, including instructions on how to review your proof when it is ready. Please keep in mind that we are working through a large volume of accepted articles, so please give us a few days to review your paper and let you know the next and final steps.

Lastly, if your institution or institutions have a press office, please let them know about your upcoming paper now to help maximize its impact. If they'll be preparing press materials, please inform our press team within the next 48 hours. Your manuscript will remain under strict press embargo until 2 pm Eastern Time on the date of publication. For more information, please contact onepress@plos.org.

You will receive an invoice from PLOS for your publication fee after your manuscript has reached the completed accept phase. If you receive an email requesting payment before acceptance or for any other service, this may be a phishing scheme. Learn how to identify phishing emails and protect your accounts at https://explore.plos.org/phishing.

If we can help with anything else, please email us at customercare@plos.org.

Thank you for submitting your work to PLOS One and supporting open access.

Kind regards,

PLOS One Editorial Office Staff

on behalf of

Dr. Szabolcs Blazsek

Academic Editor

PLOS One
